# Distinguishing between different percolation regimes in noisy dynamic networks with an application to epileptic seizures

**DOI:** 10.1371/journal.pcbi.1011188

**Published:** 2023-06-16

**Authors:** Xiaojing Zhu, Heather Shappell, Mark A. Kramer, Catherine J. Chu, Eric D. Kolaczyk

**Affiliations:** 1 Department of Mathematics and Statistics, Boston University, Boston, Massachusetts, United States of America; 2 Department of Biostatistics and Data Science, Wake Forest School of Medicine, Winston-Salem, North Carolina, United States of America; 3 Massachusetts General Hospital, Harvard Medical School, Boston, Massachusetts, United States of America; 4 Department of Mathematics and Statistics, McGill University, Montreal, Quebec, Canada; Scuola Internazionale Superiore di Studi Avanzati, ITALY

## Abstract

In clinical neuroscience, epileptic seizures have been associated with the sudden emergence of coupled activity across the brain. The resulting functional networks—in which edges indicate strong enough coupling between brain regions—are consistent with the notion of percolation, which is a phenomenon in complex networks corresponding to the sudden emergence of a giant connected component. Traditionally, work has concentrated on noise-free percolation with a monotonic process of network growth, but real-world networks are more complex. We develop a class of random graph hidden Markov models (RG-HMMs) for characterizing percolation regimes in noisy, dynamically evolving networks in the presence of edge birth and edge death. This class is used to understand the type of phase transitions undergone in a seizure, and in particular, distinguishing between different percolation regimes in epileptic seizures. We develop a hypothesis testing framework for inferring putative percolation mechanisms. As a necessary precursor, we present an EM algorithm for estimating parameters from a sequence of noisy networks only observed at a longitudinal subsampling of time points. Our results suggest that different types of percolation can occur in human seizures. The type inferred may suggest tailored treatment strategies and provide new insights into the fundamental science of epilepsy.

## Author summary

Prior work has shown that an explosive density increase (i.e., more edges) in the brain functional connectivity networks in epilepsy patients during seizure onset aligns with the notion of percolation—a phenomenon in complex networks corresponding to the sudden emergence of a giant connected component. Our work delves deeper to provide statistical methods to uncover the underlying network evolution behavior behind the density increase. We aim to answer the question: How can we distinguish between different percolation regimes in practice? We develop a class of random graph hidden Markov models (RG-HMMs) and the necessary inferential methodologies applicable to real-world context, for characterizing percolation regimes in dynamically evolving networks in the presence of edge birth, death and noise. We present an EM algorithm with particle filtering and data augmentation for estimating parameters, and a hypothesis testing framework using Bayes factor for inferring between the Erdos-Renyi (a classical type) and the product-rule (an explosive type) percolation regimes. Our application to real seizure data suggests that different types of percolation can occur at different stages of human seizures depending on the clinical seizure types.

## Introduction

Epilepsy is a common neurological syndrome, affecting over 70 million people worldwide, and a major burden with respect to quality of life, morbidity, and risk of premature mortality [1]. It is well known now that synchronization of neurons (and on the macro-scale—groups of neurons that make up larger brain regions) play a pivotal role in maintaining normal brain function. The abnormal synchronization of neurons, however, is a defining characteristic of some neurological disorders, such as epilepsy.

In recent years, it has become increasingly clear that epilepsy and seizures result not only from isolated brain areas, but from networks of interacting brain regions [2]. More specifically, epileptic seizures have been associated with the sudden emergence of coupled/synchronized activity across the brain [3–9]. This synchronized activity may be defined via functional brain networks, where the nodes of the network represent distinct brain regions, and the edges between the nodes indicate strong coupling of brain activity [10]. Moreover, the emergence of coupled activity (or increased network edges), aligns with the notion of *percolation*—the sudden emergence of a giant connected component (GCC) in a network [11, Chapter 3].

In percolation theory, the behavior of the GCC in a network is studied as a function of its evolution over time. Two popular models of percolation are the Erdos-Renyi (ER) model [12] and the Achlioptas’ process [13]. They are both random graph models assuming single-edge change over time, but they differ in the choice of which edge changes. The choice of edge addition in the ER model is uniform over all non-edges, while that in the Achlioptas’ process model is based on a ‘product rule’ (PR), which slows down the growth of the GCC by favoring the creation of edges between smaller connected components. Percolation in the ER model is considered a classical archetype, while that in the PR process is of a more rapid form. As such, these two percolation models represent prototypical extremes.

**Fig 1:**
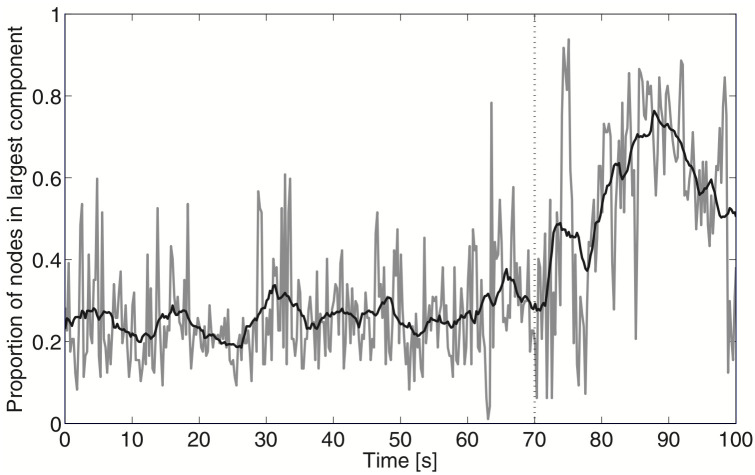
Proportion of nodes in the largest component as a function of time for a functional connectivity network deduced from the electrocorticogram of a single patient with epilepsy during a seizure. The black trace is a smoothed version of this process. Dotted vertical line indicates seizure onset. Source: [14].

Fig 1 depicts an example of functional brain network behavior during seizure onset. As can be seen in the figure, the size of the largest connected component of the brain network increases dramatically following the clinically determined onset of the epileptic seizure. The behavior of the curve in this figure is qualitatively similar to that of a percolation curve, in which a network is undergoing a transition from a large collection of small networks to a single large connected network.

Increased interest in network percolation has recently been fueled by its relevance to epileptic seizures [14–17]. While prior work has shown an explosive density increase (i.e. more edges) in functional connectivity networks in epilepsy patients during seizure onset, aligning with the notion of percolation, our work delves deeper to provide methods to uncover the underlying network evolution behavior behind the density increase. We aim to answer the question: How can we distinguish between different percolation regimes in practice? Understanding such phenomena, corresponding with the transition between normal brain function, seizure propagation, and seizure termination, may be critical for both the fundamental science of epilepsy and developing improved strategies for treatment.

**Fig 2:**
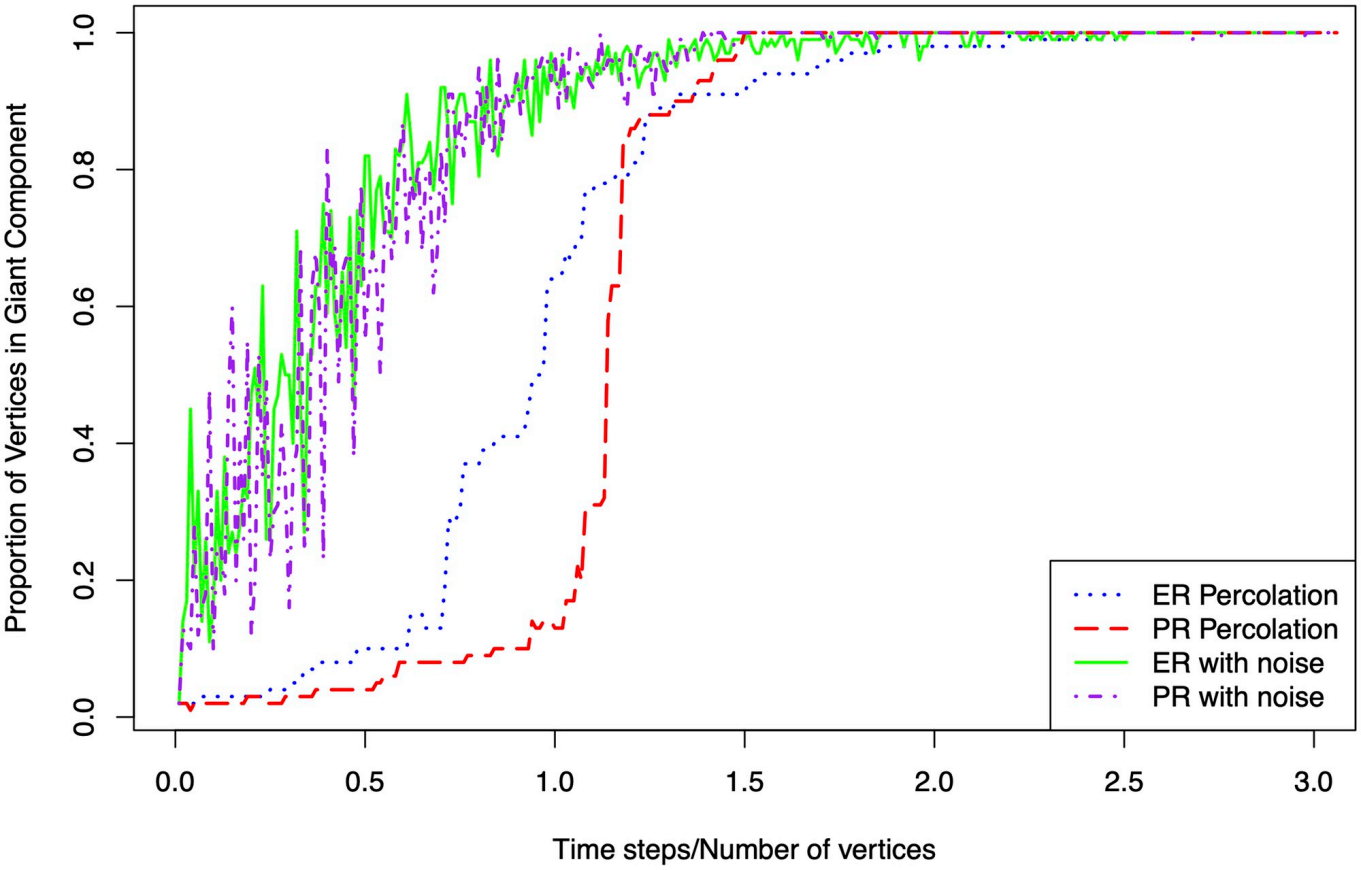
Proportion of nodes in the largest component as a function of scaled time for ER and PR birth and death process with and without noise on network with 100 vertices. Observe that the processes are virtually indistinguishable with noise.

In this paper, we propose a framework to distinguish between different types of phase transitions undergone in a seizure, and in particular, to distinguish between different percolation regimes. Traditionally, work in this area has concentrated on noise-free percolation with a monotonic process of network growth, but real-world networks are more complex. It is more realistic to consider both edge creation and dissolution in network evolution. Furthermore, we should expect observed networks to be contaminated by noise, such as measurement error. The presence of edge death and noise significantly confounds the distinction between percolation regimes and makes the two percolation models indistinguishable using heuristic statistics, e.g. the size of the largest component, as shown in Fig 2. Therefore, a framework for the statistical testing of competing hypotheses of percolation regimes, under these conditions, is needed.

We develop a class of random graph hidden Markov models (RG-HMMs) and the necessary inferential methodologies, for characterizing percolation regimes in noisy, dynamically evolving networks in the presence of edge birth and edge death. Our model class builds on the framework proposed (with only preliminary inferential machinary) by [14], where the nonstationary process characterized by birth and death of edges was modeled in a hidden/latent layer in discrete time, assuming the true underlying networks evolve by a single edge change per time step. Such models cannot be directly used in application since it is nearly impossible to observe every single edge change on real-world data. We extend the model to the continuous-time setting where the process may stay in different states for differing (continuous) amounts of time, and assume that there are many non-observed single edge changes occurring between the consecutive observation times. This is critical for making the framework applicable to epilepsy, as well as other real-world contexts, in which, even if observed at regular intervals, a dynamically evolving network can almost never practically be observed at the resolution of changes in individual edge status.

**Fig 3:**
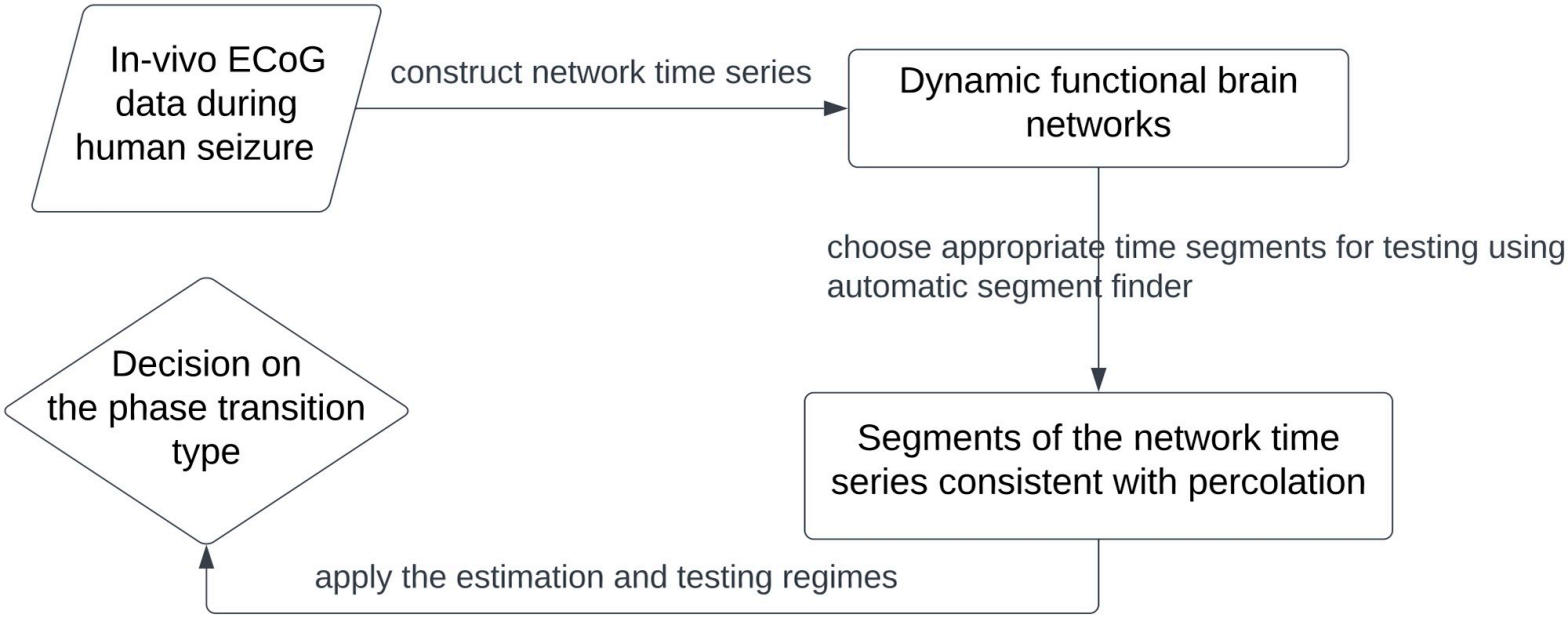
Schematic of the pipeline for inferring percolation types in epileptic seizures. To infer the transition types of a seizure, we first construct a time series of functional connectivity networks from the ECoG data during the seizure. Then we identify a few segments of the network time series consistent with percolation using an automatic procedure we developed. Finally for each segment we apply the statistical testing procedure to infer the percolation regime.

On top of the novel model class, we develop a testing approach using Bayes factor to distinguishing between the Erdos-Renyi (a classical type) and the product-rule (an explosive type) percolation regimes in epileptic seizures. Fig 3 shows a schematic diagram of the overall pipeline we use for inferring the type of phase transition undergone in epileptic seizures. This pipeline involves firstly constructing time series of functional connectivity networks from the electrocorticography (ECoG) data, then identifying segments consistent with percolation for testing, and finally applying the testing approach we developed to each segment to infer the percolation regime.

This paper is organized as follows. In the ‘Materials and Methods’ section we provide an overview of the epileptic seizure data used in this study, model definitions for continuous-time percolation models and our HMM set-up. We also outline a statistical testing framework for competing percolation regimes using Bayes factor, which involves an expectation-maximization (EM) algorithm for obtaining maximum likelihood (ML) estimates of the model parameters, and a forward algorithm to approximate the probabilities of the observed networks. In the ‘Results’ section we report simulation results for our estimation and testing algorithms. We also present application results for the proposed framework to real epileptic seizure data. Finally, we conclude the study with a discussion.

## Materials and methods

In this section, we describe data and provide model definitions for continuous-time percolation models and our HMM set-up. An expectation-maximization (EM) algorithm for obtaining maximum likelihood (ML) estimates of the model parameters are described. We also outline a statistical testing framework for distinguishing between two competing percolation regimes.

### Data

**Fig 4:**
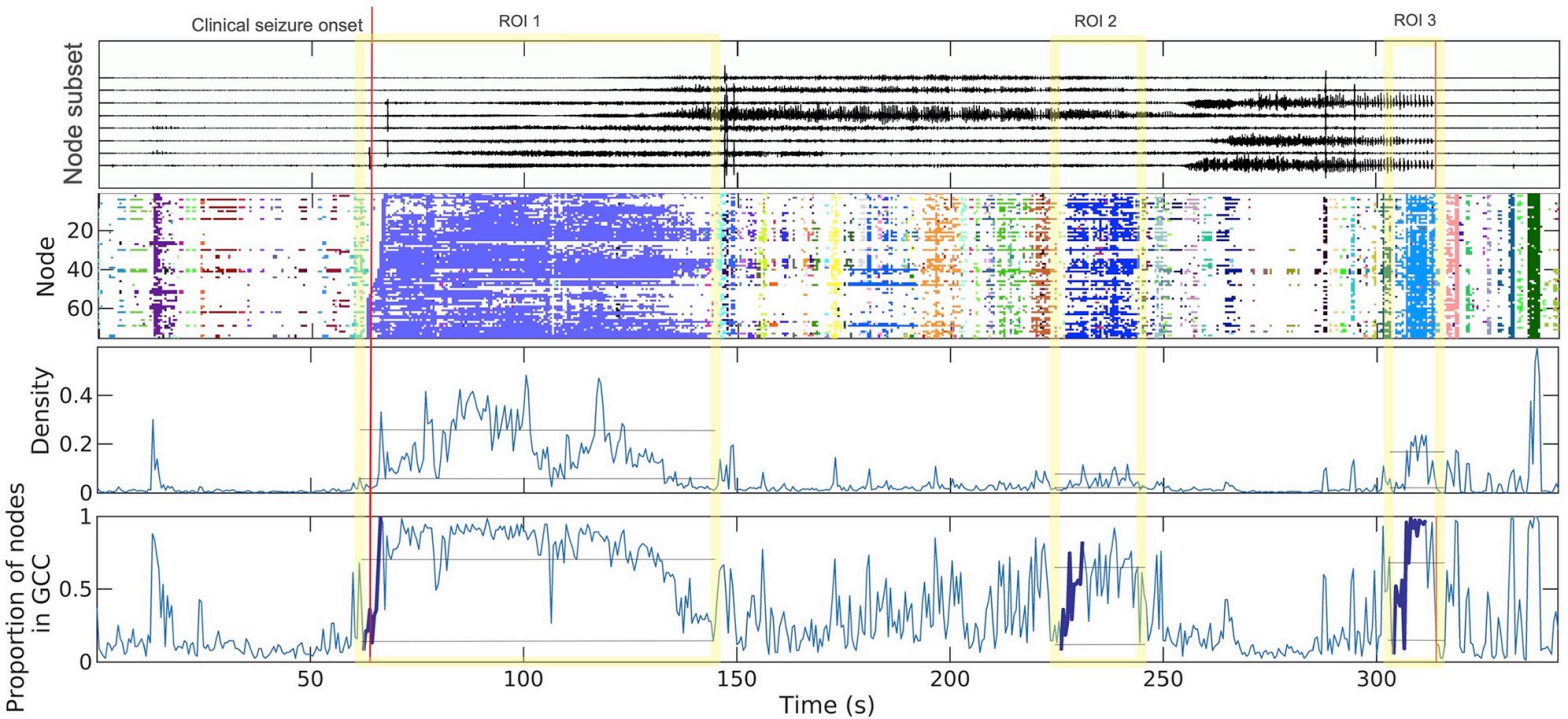
Seizure data (both voltage time series and constructed dynamic networks) visualization for one seizure. Vertical red lines represent clinically determined seizure onset and termination times. *Top*: Voltage time series recorded at eight electrodes on left brain hemisphere. *Second row*: Community membership (indicated colors) for each node over time; the three large dynamic communities in shades of blue manifest three ROIs. *Third row*: Network density over time. *Bottom*: Proportion of nodes in the GCC over time; the bold curves in ROIs are chosen by automatic segment finder for testing. The horizontal lines in gray represent the first and third quartiles of density/GCC within each ROI.

The electrocorticography data consist of invasive brain voltage recordings for 3 seizures from a patient with epilepsy. The entire array of electrodes consisted of 106 electrodes on the left hemisphere and 70 electrodes on the right hemisphere, with 1024 Hz sampling rate. The top row in Fig 4 illustrates an example of seizure voltage dynamics where the voltage time series recorded at 8 electrodes on the left brain hemisphere for one seizure are plotted.

For each seizure, we construct a time series of functional connectivity networks based on the voltage recordings in the following manner. We first band-pass filter the data between 4 and 50 Hz, and compute a bipolar reference by subtracting the activity of neighboring electrodes. Then we divide the re-referenced data into 1 s windows and 0.5 s overlap beginning 120 s before seizure onset and ending 30 s after seizure termination: the seizure onset and termination times were clinically determined. Finally, we construct a functional network for each 1 s window by identifying significant cross-correlations between each pair of electrodes and controlling for multiple comparisons using the FDR (*q* = 0.5). Details of this functional network construction procedure can be found in [18] and [8].

Among the 3 seizures, two were clinically determined to be focal seizures with secondary generalization, and one was clinically determined to be a focal seizure without secondary generalization (i.e., the seizure activity remained local). We focus our analysis on the left hemisphere because each seizure begins in this hemisphere and we expect percolation to occur near the location of seizure onset as this region recruits other brain areas into the seizure dynamics. To that end, we construct functional networks using the 75 channels on the left hemisphere involved in seizure onset or propagation.

We plot the size of the GCC over time, and edge density over time, for the resulting network time series from the first seizure in the bottom two rows of Fig 4. Plots for the second and third seizures are provided in section 7 of S1 Text.

### Model definition

We first introduce two continuous-time percolation models allowing for both birth and death of edges: an Erdos-Renyi (ER) process and a product rule (PR) process, which build off of the work of [14] on discrete-time percolation processes. Both models represent continuous-time network evolution as the result of many one-edge changes over time. We then overlay the hidden Markov model framework on top of the percolation models to capture the presence of noise in the observations, which gives us the random graph hidden Markov model (RG-HMM). Throughout the paper, we use capitals to denote random variables. Additionally, we only consider networks with a fixed vertex set.

#### Continuous-time percolation models

The ***continuous-time birth and death Erdos-Renyi (ER) process*** is a network-valued bivariate continuous-time Markov chain {*W*(*t*), *G*(*t*), *t* ≥ 0} taking on values in a finite set X. *G*(*t*) is an undirected-graph variable with *N* nodes. The state space of this network-valued variable is the space of all simple graphs on *N* nodes, which is of cardinality $2^{\left(\begin{array}{c} N\\ 2 \end{array}\right)}$. *W*(*t*) is a binary variable with state space {0, 1}, indicating a single edge is either added (*W*(*t*) = 1) or deleted (*W*(*t*) = 0) to produce *G*(*t*) at the most recent transition time. This process has the following properties:

(i) at each transition time *τ*_1_ with state {*W*(*τ*_1_) = *w*, *G*(*τ*_1_) = *g*}, the amount of time it spends in that state before making a transition into a different state is exponentially distributed with rate parameter *γ* > 0;
(ii) for two consecutive transition times *τ*_1_ < *τ*_2_, the network *G*(*τ*_2_) differs from *G*(*τ*_1_) by a single edge, and the edge is chosen to be added or deleted uniformly at random on non-edge set or edge set of *G*(*τ*_1_), the choice of which depends on the binary variable *W*(*τ*_2_). The transition matrix for the binary variable, when *G*(*τ*_1_) is neither empty nor complete, is shown in Table 1. *p* and *q* are respectively birth and death rates, and are not required to sum to 1. When *G*(*τ*_1_) is a complete graph, *W*(*τ*_2_) = 0 with probability 1 (i.e. the growth of edges are not possible since all possible edges exist). When *G*(*τ*_1_) is an empty graph, *W*(*τ*_2_) = 1 with probability 1 (i.e. the death of edges are not possible since none exist).

**Table 1:** Transition probability matrix for *W*(*t*) at two consecutive transition time (*τ*_1_, *τ*_2_), *τ*_1_ < *τ*_2_, when *G*(*τ*_1_) is neither empty nor complete.

	*W*(*τ*_2_) = 0	*W*(*τ*_2_) = 1
*W*(*τ*_1_) = 0	1 − *p*	*p*
*W*(*τ*_1_) = 1	*q*	1 − *q*

The ***Continuous-time birth and death product rule (PR) process*** is analogous to the aforementioned birth and death ER process, except for the choice of which edge is added or deleted at each transition time. In the case of the ER model, such choice is uniform over current non-edge set or edge set. However, in the case of the PR model, the choice depends on the modular structure of the current network.

Let *E*_*t*_ denote the edge set of current network *G*(*t*), and $E_{t}^{C}$ the non-edge set. For the upcoming transition time *τ*, if *W*(*τ*) = 1, the choice of which edge to add is done in the following manner.

(i) Uniformly choose two candidate vertex pairs among all edges in $E_{t}^{C}$ (i.e. among all non-edges), and denote the two vertex pairs by *e*_1_ = (*v*_11_, *v*_12_) and *e*_2_ = (*v*_21_, *v*_22_).
(ii) Evaluate the size of the connected components to which *v*_11_, *v*_12_, *v*_21_, *v*_22_ belong, and denote them by *C*_11_, *C*_12_, *C*_21_, *C*_22_, respectively.
(iii) Apply the following product rule in [13]: If |*C*_11_||*C*_12_| < |*C*_21_||*C*_22_|, then add edge *e*_1_, otherwise, add edge *e*_2_.

The death of the an edge is handled in an analogous manner. If *W*(*τ*) = 0, we uniformly choose two candidate vertex pairs among all edges in *E*_*t*_, and similarly evaluate the size of the connected components to which *v*_11_, *v*_12_, *v*_21_, *v*_22_ would belong if their edges were absent. If |*C*_11_||*C*_12_| < |*C*_21_||*C*_22_|, then delete edge *e*_2_. Otherwise, delete edge *e*_1_. Note that the Achlioptas product rule slows down the growth of the GCC by favoring the creation of edges between small connected components.

#### Random Graph Hidden Markov Model (RG-HMM)

We now consider observing the continuous-time network evolution at discrete time points with errors. Assume that we have *M* repeated network-valued observations $g_{1:M}^{⋆}=[g^{⋆}(t_{1}),g^{⋆}\left(t_{2}\right),\cdots ,g^{⋆}\left(t_{M})\right]$, where *t*_1_ < *t*_2_ < … < *t*_*M*_ are *M* observation time points. Let $G_{1:M}^{⋆}=\left[G^{⋆}\left(t_{1}\right),\cdots ,G^{⋆}\left(t_{M}\right)\right]$ denote the random vector of observed networks, of which $g_{1:M}^{⋆}$ is a realization. We represent a true/hidden network variable underlying the observed network at a particular observation time by *G*(*t*_*m*_), and the hidden binary variable at the observation time by *W*(*t*_*m*_). We assume that

(i) the latent networks evolve according to a percolation model. Then the true/hidden variables at discrete observation times {*W*(*t*_*m*_), *G*(*t*_*m*_), *m* = 1, 2, …, *M*} are embedded in the continuous-time Markov chain (ER/PR process), thus constituting a discrete-time Markov chain,
(ii) there is a time-independent error process, which corrupts the observing process by type-I error rate and type-II error rate, denoted respectively by *α* and *β*. Specifically, for any *α*, *β* ∈ [0, 1], we have
  $$\begin{array}{c} \begin{array}{c} P(G^{⋆}\left(e\right)=0|G\left(e\right)=0)=1-\alpha ,\;P\left(G^{⋆}\left(e\right)=1|G\left(e\right)=0\right)=\alpha \\ P(G^{⋆}\left(e\right)=1|G\left(e\right)=1)=1-\beta ,\;P\left(G^{⋆}\left(e\right)=0|G\left(e\right)=1\right)=\beta , \end{array} \end{array}$$ (1)
  where *G*^⋆^(*e*) = 1 or 0 represent a specific edge is present or not in observed variable, and *G*(*e*) = 1 or 0 represent a specific edge is present or not in hidden variable,
(iii) the first observation *G*^⋆^(*t*_1_) is error-free.

**Fig 5:**
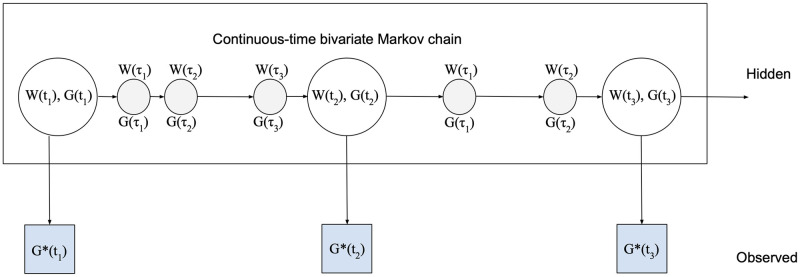
RG-HMM set-up. The unobserved hidden variables evolve according to a continuous-time Markov process, and we observe networks at discrete observation times with error. The unobserved small changes occurring between the consecutive observation times are represented in gray.

The last assumption is for convenience and standard. Combining the network-valued latent Markov chain with the error process, we obtain a random graph hidden Markov model (RG-HMM). The schematic representation is shown in Fig 5.

### Hypothesis testing of putative percolation regimes

Let Γ denote the parameters *p*, *q*, *γ*, *α*, *β* in the percolation models. We now present a hypothesis testing framework using Bayes factors for distinguishing between two percolation models, ER and PR. The testing problem can be formulated as a test of separate families of hypotheses, i.e.

$$\begin{array}{c} H_{ER}\;:\;g^{⋆}∼f\left(g^{⋆},\Gamma_{ER}\right)\;\text{vs}\;H_{PR}\;:\;g^{⋆}∼g\left(g^{⋆},\Gamma_{PR}\right), \end{array}$$ (2)

where ***g***^⋆^ = [*g*^⋆^(*t*_1_), *g*^⋆^(*t*_2_), …, *g*^⋆^(*t*_*M*_)] is the observed sequence of networks, and *f*(⋅, **Γ**_*ER*_) and *g*(⋅, **Γ**_*PR*_) are the probability functions of ***G***^⋆^ under the ER process with parameters **Γ**_*ER*_ and PR process with parameters **Γ**_*PR*_. *H*_*ER*_ and *H*_*PR*_ are the hypotheses that the observed sequences ***g***^⋆^ is from the ER process and PR process, respectively.

#### Bayes factor

Following [19] on tests of separate families of hypotheses, we adopt a Bayesian approach to this testing problem. The posterior odds for *H*_*ER*_ vs *H*_*PR*_ are, by Bayes’s theorem,

$$\begin{array}{c} \frac{pr(H_{ER}|g^{⋆})}{pr(H_{PR}|g^{⋆})}=\frac{{\tilde{\omega }}_{f}\int f\left(g^{⋆},\Gamma_{ER}\right)p_{f}\left(\Gamma_{ER}\right)d\Gamma_{ER}}{{\tilde{\omega }}_{g}\int g\left(g^{⋆},\Gamma_{PR}\right)p_{g}\left(\Gamma_{PR}\right)d\Gamma_{PR}}, \end{array}$$ (3)

where ${\tilde{\omega }}_{f}$ and ${\tilde{\omega }}_{g}$ are the prior probabilities of *H*_*ER*_ and *H*_*PR*_ being true, respectively. *p*_*f*_(**Γ**_*ER*_) is the prior p.d.f of **Γ**_*ER*_ under *H*_*ER*_, and *p*_*g*_(**Γ**_*PR*_) is the prior p.d.f of **Γ**_*PR*_ under *H*_*PR*_.

The Bayes factor (*BF*) is the posterior odds for *H*_*ER*_ vs *H*_*PR*_ when the prior probabilities of the two hypotheses are equal, i.e. when ${\tilde{\omega }}_{f}={\tilde{\omega }}_{g}=0.5$. Exact analytic calculation of the Bayes factor for the testing problem in (2) is not tractable, so we resort to numerical methods. [20] proposed two large-sample approximations for Bayes factors—Laplace’s Method and the Schwarz Criterion. The approximation from Laplace’s method requires specifying the prior distributions *p*_*f*_(**Γ**_*ER*_) and *p*_*g*_(**Γ**_*PR*_) on parameters of each model. However, the choice of priors is not trivial for a percolation model. Therefore, we choose to use the approximation from the Schwarz criterion which has the benefit of not requiring priors. Also note that because the dimensions of **Γ**_*ER*_ and **Γ**_*PR*_ are the same, the logarithm of the Bayes factor for our testing problem can be approximated by the log-likelihood difference, i.e.

$$\begin{array}{c} \text{log}\;BF\approx \text{log}\;f\left(g^{⋆},{\hat{\Gamma }}_{ER}\right)-\text{log}\;g\left(g^{⋆},{\hat{\Gamma }}_{PR}\right), \end{array}$$ (4)

where ${\hat{\Gamma }}_{ER}$ and ${\hat{\Gamma }}_{PR}$ are the MLEs under *H*_*ER*_ and *H*_*PR*_, respectively.

Two subroutines are needed for computing the Bayes factor: 1) one is the ML estimation of the parameters under the two percolation models to compute ${\hat{\Gamma }}_{ER}$ and ${\hat{\Gamma }}_{PR}$; 2) the other is the computation of the marginal probabilities of the observed networks, $\text{log}\;f(g^{⋆},{\hat{\Gamma }}_{ER})$ and $\text{log}\;g(g^{⋆},{\hat{\Gamma }}_{PR})$.

In the following two sections, we provide high-level summaries of the two subroutines respectively and defer the details to section 1 and 2 of S1 Text.

#### MLE for RG-HMM

Although our ultimate goal is testing for two competing hypotheses of percolation regimes, learning the RG-HMM from a sequence of noisy networks observed only at a longitudinal subsampling of times is a necessary precursor and of independent interest.

Throughout this section, we assume *α* < 0.5 and *β* < 0.5. Under such an assumption, the parameters in the model are identifiable. (A proof is provided in section 4 of S1 Text.) We present an Expectation-Maximization (EM) algorithm for estimating the parameters in an RG-HMM (i.e. *p*, *q*, *γ*, *α*, *β*) with a given sequence of noisy networks observed only from a longitudinal subsampling of time points, i.e. $g_{1:M}^{⋆}$. We assume that the network in the hidden layer changes faster than the observing rate so that there are likely many non-observed small changes occurring between the consecutive observation times, as is typically the case for functional connectivity networks used in the study of epilepsy.

Estimation is done conditional on the latent state *W*(*t*_1_) = 1, *G*(*t*_1_) = *g*^⋆^(*t*_1_) at the first observation time *t*_1_. As in [21], this has the advantage that no initial distribution assumption is needed for the latent Markov chain, and the estimated parameters refer exclusively to the dynamics of the network.

The algorithm consists of an E-step, wherein we calculate the expected value of the complete data log-likelihood $l\left(\alpha ,\beta ,\gamma ,p,q\right)=\text{log}\;f\left(W_{2:M},G_{2:M},g_{2:M}^{⋆}\right)$, with respect to the distribution of unknown latent variables ***W***_2:*M*_, ***G***_2:*M*_, given the observed networks $g_{1:M}^{⋆}$ and the current parameter estimates. We then maximize the expected log-likelihood in the M-step.

The E-step is non-trivial in that there are two levels of unknown latent variables in our model: we do not know the true networks and binary variables *W*(*t*_*m*_), *G*(*t*_*m*_) at the observation times, nor do we know the intermediate path that connects one true state *W*(*t*_*m*−1_), *G*(*t*_*m*−1_), to the next *W*(*t*_*m*_), *G*(*t*_*m*_). EM is standard for parameter estimation in general HMMs. However, we face some unique challenges: (1) direct calculation of the expectation is computationally infeasible due to the enormously large state space of size $2^{\left(\begin{array}{c} n\\ 2 \end{array}\right)}$; (2) the observed-data likelihood is difficult to calculate due to the unobserved changes occurring between consecutive observation times. Particle filtering and augmented data sampling using MCMC are used to tackle these challenges. Details on the ML estimation regime are provided in section 1 of S1 Text.

#### Marginal probability of observations

The computation of the probabilities of the observed networks under the two percolation models, $\text{log}\;f(g^{⋆},{\hat{\Gamma }}_{ER})$ and $\text{log}\;g(g^{⋆},{\hat{\Gamma }}_{PR})$, is the key ingredient in (4) for the test. We now present a forward algorithm with particle filtering to approximate such quantities in our RG-HMM. Let ***X***_*m*_ denote the pair (*W*(*t*_*m*_), *G*(*t*_*m*_)). Similar to the ML estimation, testing is also done conditional on the latent state *W*(*t*_1_) = 1, *G*(*t*_1_) = *g*^⋆^(*t*_1_) at the first observation time *t*_1_. Let ***X***_*m*_ denote the pair (*W*(*t*_*m*_), *G*(*t*_*m*_)). Define $Z_{m}\;≔\;P(G_{2:m}^{⋆}=g_{2:m}^{⋆}|X_{1}=(1,g^{⋆}\left(t_{1}\right)))$, *m* = 2, ⋯ *M*, and *Z*_1_ ≔ 1. Let $q_{m}\left(x\right)=P\left(G_{m}^{⋆}=g_{m}^{⋆}|X_{m}=x\right)$, $\eta_{m}\left(x\right)=P\left(X_{m}=x\right|G_{2:m-1}^{⋆}=g_{2:m-1}^{⋆},X_{1}=\left(1,g^{⋆}\left(t_{1}\right)\right)$, *m* = 2, ⋯ *M*. Under such notations, *Z*_*M*_ is the marginal probability of observations of our interest. With the recurrence relation (the derivation is provided in section 2.1 of S1 Text),

$$\begin{array}{c} Z_{m}=Z_{m-1}\sum_{x}q_{m}\left(x\right)\eta_{m}\left(x\right)=Z_{m-1}E\left(q_{m}\left(X\right)\right),X∼\eta_{m}\left(\cdot \right) \end{array}$$ (5)

the probabilities *Z*_*M*_ can be approximated recursively by replacing each *η*_*m*_(⋅) in (5) with $\eta_{m}^{B}\left(\cdot \right)$, which is the particle approximation of the filtering distribution *η*_*m*_(⋅). A forward algorithm with particle filtering is provided in section 2.2 of S1 Text to compute $Z_{M}^{B}$, i.e. the particle approximation of the marginal probability of observations. Details on the marginal probability approximation regime are provided in section 2 of S1 Text.

## Results

We first evaluate the performance of the proposed estimation and testing approaches using simulated data. We then apply the approaches to the real human epileptic seizure data to infer the type of phase transitions undergone in each seizure. Both results on the synthetic data and the real data are provided in the following two sections.

### Simulation results

In this section, we conduct simulations to evaluate the empirical performance of the proposed estimators and test. Since the performance of the test is driven by the performance of the ML estimation, we present simulation results for both estimation and testing.

#### Simulation results for estimation

We first investigate the finite sample performance of the estimation procedure for networks with *N* = 20 nodes simulated at *M* = 50 observation time points, referred to as ${\left\{t_{m}\right\}}_{m=1}^{50}$, from the ER/PR percolation process with parameter setting *p* = 0.7, *q* = 0.3, *γ* = 2 and error rates *α* = 0.03, *β* = 0.01. We set the observation rate, denoted by *κ*, to be 0.6, i.e. we set *t*_*m*_ = *m*/0.6. The observation rate is set to be smaller than the actual rate of change to mimic the scenario in application. For each ER and PR process, we generate 100 network time series with length 50 and obtain ML estimates of the model parameters from each data set. In the EM algorithm, we set *B* = 50, 000 and initialize all parameters as 0.5. The running time of one iteration on a high performance Linux computing cluster using 4 cores is approximately 10 minutes for the ER process and 20 minutes for the PR process under this setting. The mean and standard deviation of parameter estimates based on 100 replications for both the ER process and PR process are reported in Table 2. In Table 2, we are seeing bias in the estimates, especially for type-I and type-II error rates, which is not unexpected given that we are approximating the distribution on a very large dimensional state space with only *B* = 50, 000 particles. In principle, the larger *B* is, the more accurate estimates will be. However, it comes with the trade-off between computational time and accuracy.

**Table 2:** Mean and standard deviation of parameter estimates based on 100 simulations from ER/PR process with *B* = 50000, *N* = 20, *M* = 50.

	Birth Rate *p*	Death Rate *q*	Transition Rate *γ*	Type-I Error *α*	Type-II Error *β*
Truth	0.70	0.30	2	0.03	0.01
ER-HMM	0.680 (0.041)	0.299 (0.063)	1.746 (0.126)	0.112 (0.021)	0.037 (0.013)
PR-HMM	0.684 (0.035)	0.289 (0.057)	1.770 (0.113)	0.118 (0.019)	0.035 (0.013)

**Fig 6:**
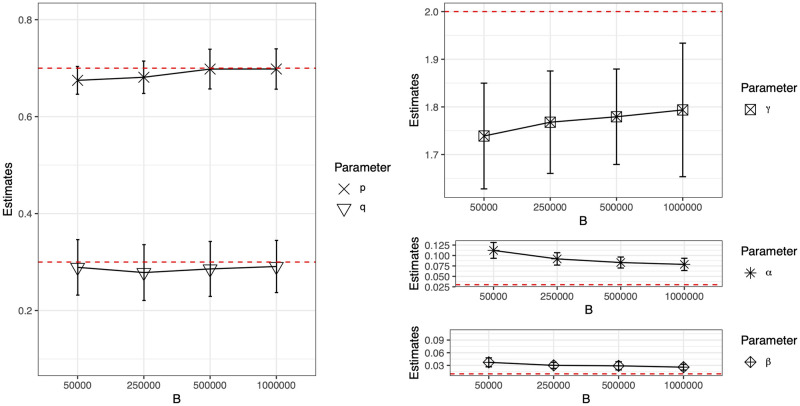
Mean and standard deviation of parameter estimates based on 20 simulations from the ER process with varying *B*, *N* = 20, *M* = 50.

**Fig 7:**
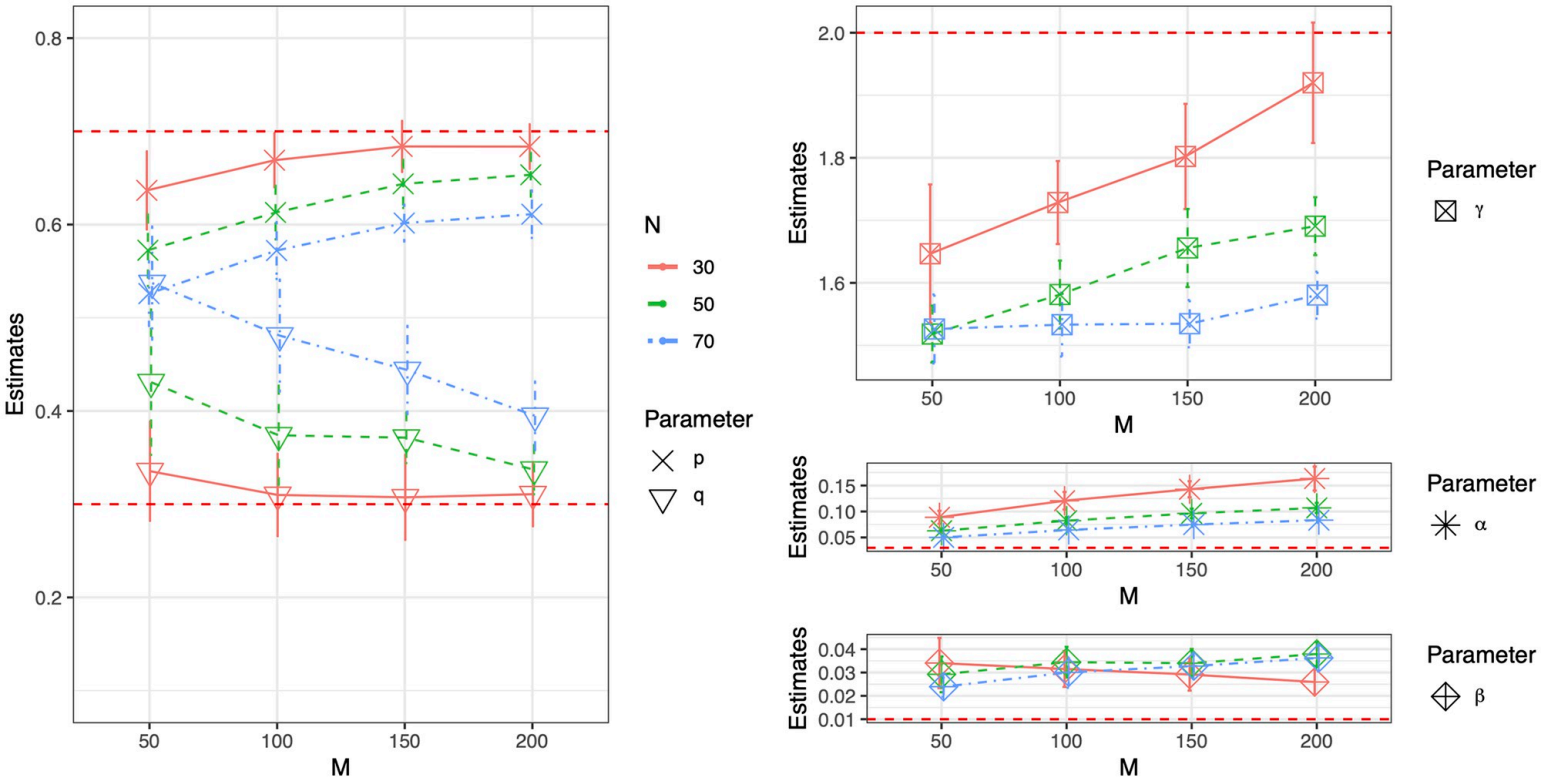
Mean and standard deviation of parameter estimates based on 20 simulations from ER process with *B* = 50000, (*N*, *M*) ∈ {30, 50, 70} × {50, 100, 150, 200}.

We then perform a small simulation study for the ER process, as a very limited exploration of the performance of MLEs with respect to *B* (the number of particles sampled at each observation moment), *M* (the number of observation time points), and *N* (network size), under the same model parameter setting as in the previous simulation study. Fig 6 shows the mean and standard deviation of parameter estimates from 20 simulated network sequences with *N* = 20 and *M* = 50 using an increasing number of particles. Fig 7 shows the mean and standard deviation of parameter estimates based on 20 simulations from an ER process with *B* = 50, 000 and (*N*, *M*) ∈ {30, 50, 70} × {50, 100, 150, 200}. Numerical results are provided in section 6.1 of S1 Text.

The main empirical findings are the following. From Fig 6, we can see that increasing *B* could reduce bias in the estimates for type-I and type-II error rates, but such improvement is diminished as *B* increases. Nevertheless, we have theoretically proved (see section 5 of S1 Text) that the MLEs in our proposed RG-HMM model are asymptotically unbiased with respect to *M*. Thus, in theory, the estimates will become unbiased when *M* is sufficient large. In Fig 7, the performance of type-I error rate estimation improves as the size of the network increases, but it does not improve as the length of the network time series (i.e. *M*) increases, using a fixed number of particles. It is not surprising that as the number of observed networks is increasing in *M*, more particles are required to approximate the parameters, in order to reveal the true asymptotic behavior as *M* increases. The type-II error rate estimate is not as sensitive to either *M* or *N*, but rather to *B*. The performance of parameter estimates of *p*, *q* and *γ* are improved as *M* increases. Additionally, for estimating these parameters from larger networks, the algorithm requires larger *M*, i.e. longer network time series with more observation time points, compared to that needed from smaller networks, in order to achieve similar performance. In summary, we can get more accurate estimates for error rates with larger *N* and *B*, with the caveat that more particles are needed for larger *M*. For the estimates of *p*, *q* and *γ*, improvement is seen as *M* and *B* increase, with the caveat that larger *M* is needed for larger *N*.

#### Simulation results for testing

To assess the effectiveness of our testing framework using Bayes factor for discriminating between two percolation models, *ER* and *PR*, we choose the rate of detection as the performance metric. For each simulated network sequence, we first compute its Bayes factor. We next identify it to be an ER sequence if the Bayes factor is greater than 1, otherwise we identify it as a PR sequence. Hence, the rate of detection for ER(PR) process is the percentage of ER(PR) sequences that are correctly identified as such among all simulated ER(PR) sequences.

**Fig 8:**
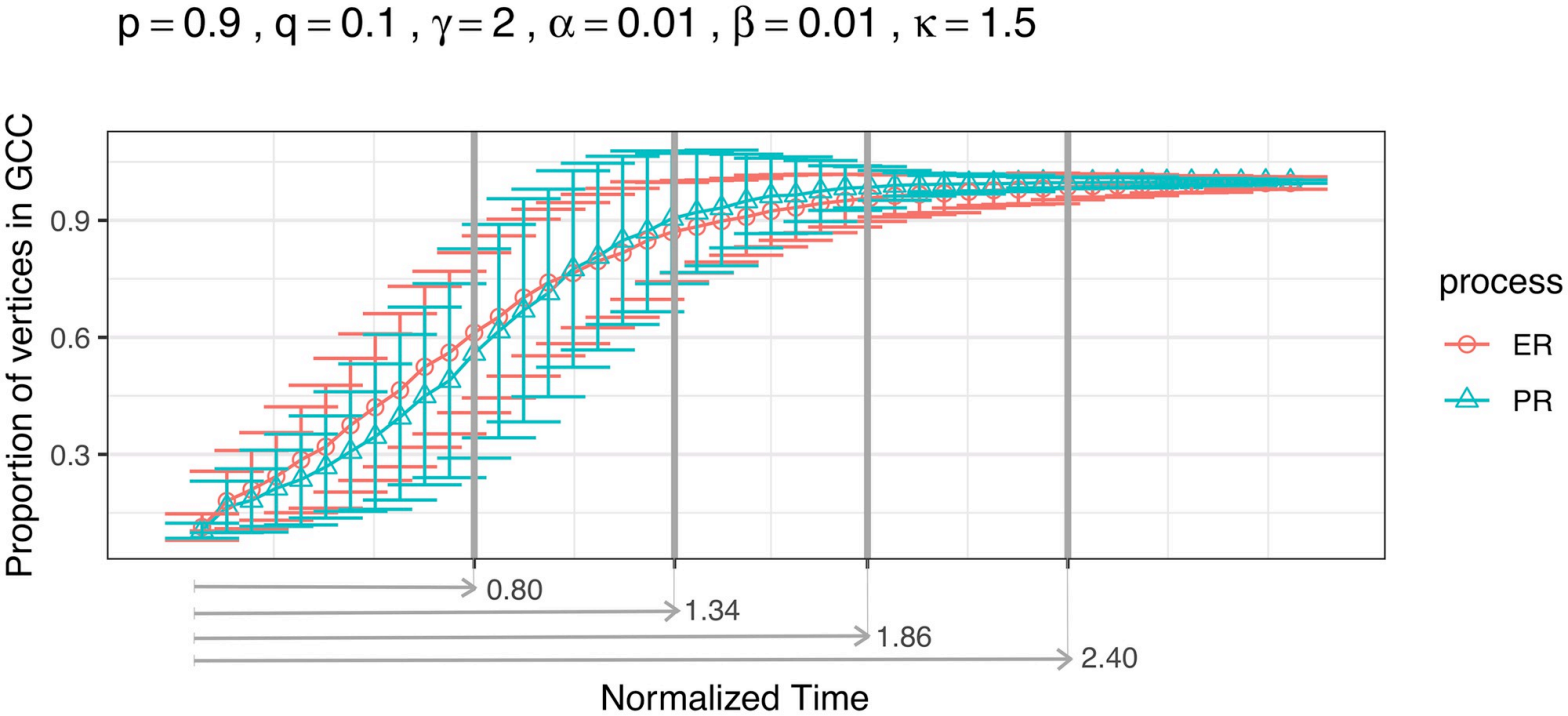
Four levels of progression ($t_{M}^{\prime }$: 0.8, 1.34, 1.86, 2.4) in percolation curve. $t_{M}^{\prime }$ is the normalized observation duration time, which represents how much of the percolation curve we take for testing.

A simulation study is designed to evaluate the effect of *B* ∈ {50, 000; 250, 000; 500, 000}, *N* ∈ {10, 20, 30}, and $t_{M}^{\prime }=\left\{0.8,1.34,1.86,2.4\right\}$ on the rate of detection for ER and PR using Bayes factor, where $t_{M}^{\prime }$ is the normalized observation duration time, which represents how much of the percolation curve we take for testing. Specifically, we define normalized observation time points as $t_{m}^{\prime }\;≔\;\frac{\gamma t_{m}}{N}$. Since observations are made with regular intervals at rate *κ*, i.e. *t*_*m*_ = *m*/*κ* for *m* = 1, ⋯, *M*, we have $t_{m}^{\prime }=\frac{m}{N(\kappa /\gamma )}$. Fig 8 illustrates the four levels of progression for the percolation curve we are considering for testing via arrowed lines. Each level of $t_{M}^{\prime }$ corresponds to a curve segment taken from the beginning of the curve to the gray line placed at the corresponding scaled time.

**Fig 9:**
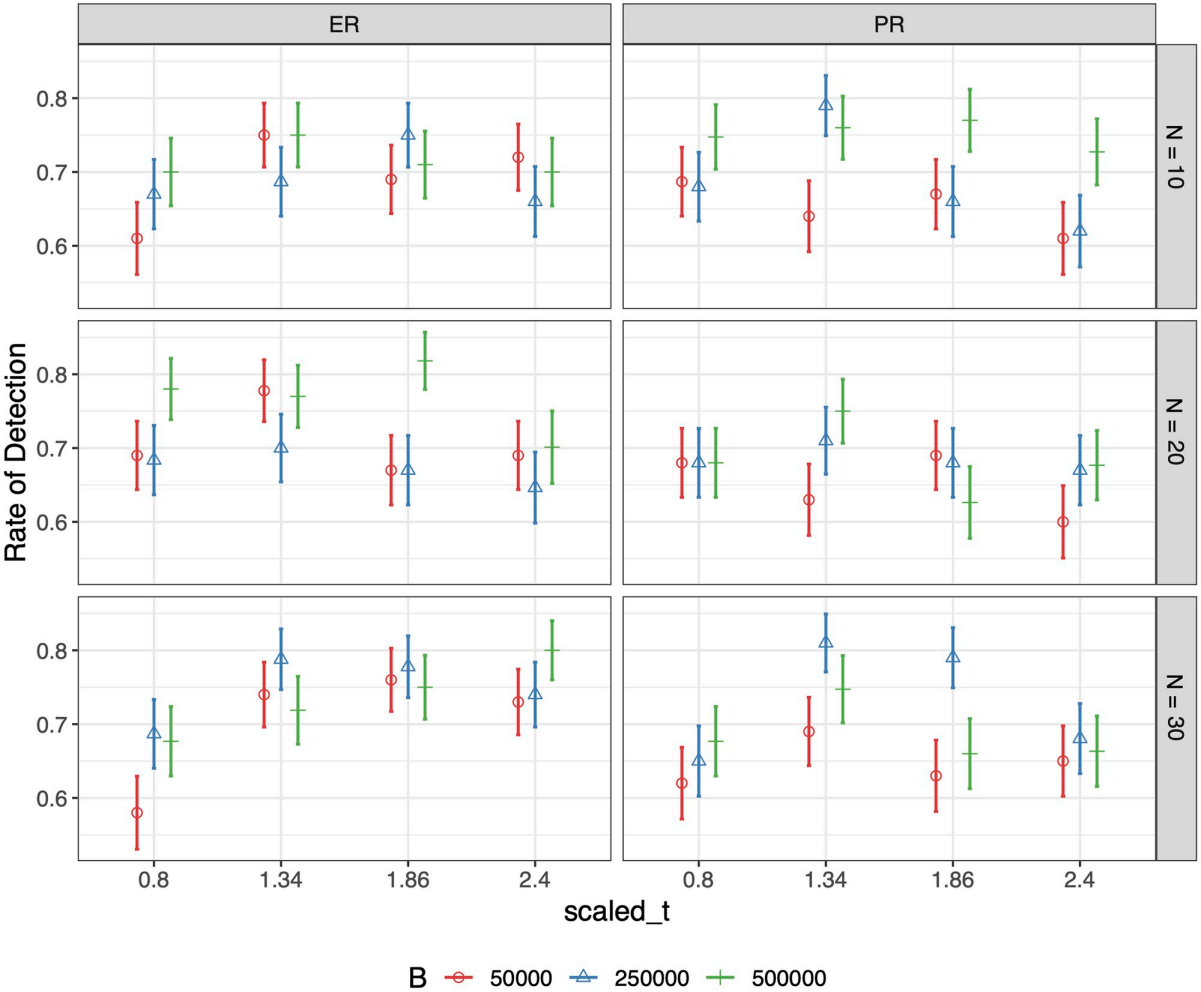
Results for the first simulation study. Rates of detection for ER and PR based on 100 replicates. N refers to the number of nodes in the networks.

**Fig 10:**
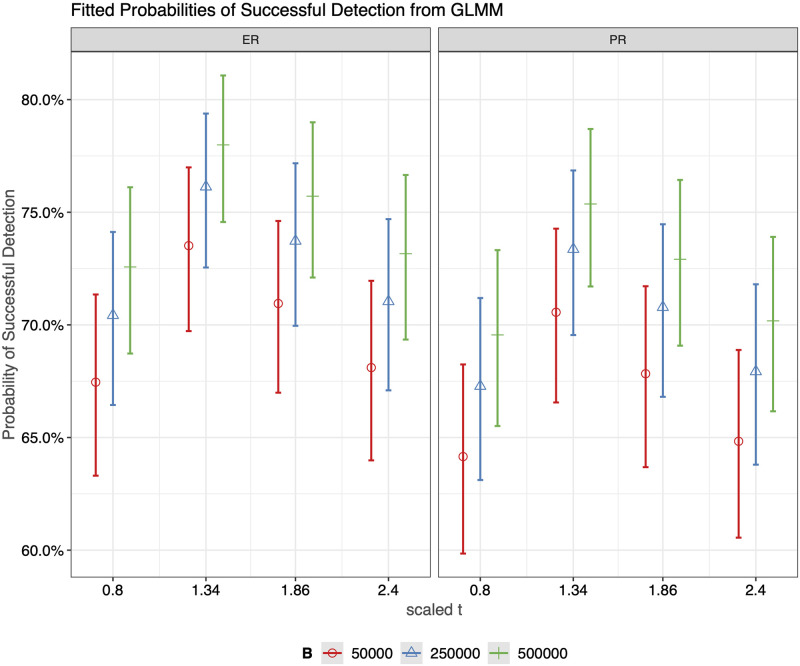
Fitted probability of successful detection from GLMM in the first simulation study.

**Fig 11:**
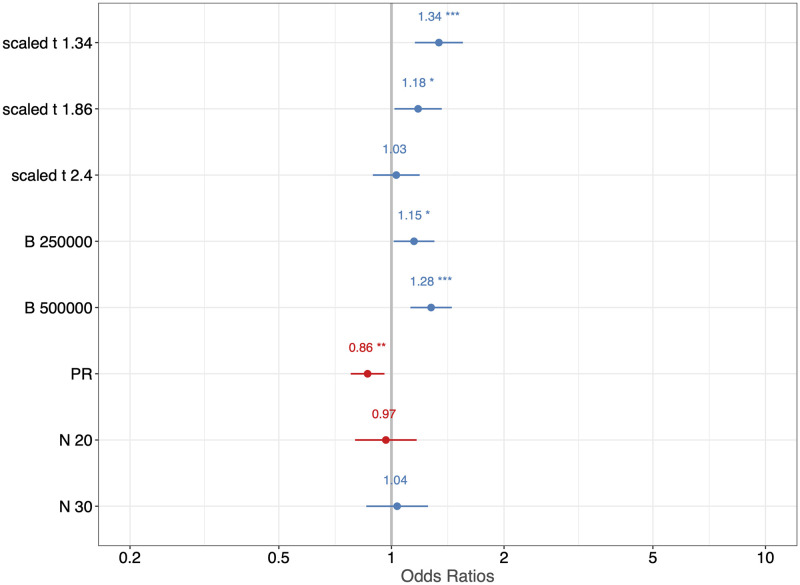
Estimates of the coefficients from GLMM in the first simulation study.

For each combination of settings, we generate 100 ER sequences and 100 PR sequences with *p* = 0.9, *q* = 0.1, *γ* = 2, *α* = 0.01, and *β* = 0.01. We set the rate of observation, *κ*, to be 1.5 and conduct hypothesis testing on each of the simulated network sequences. Rates of detection for ER and PR, based on 100 replicates, along with estimated standard errors, are shown in Fig 9. We then fit a generalized linear model with mixed effects on the testing results from the simulation study in order to analyze the relative contribution of variation in the rate of detection for ER/PR due to the change of values in *B*, *N* and $t_{M}^{\prime }$. We set the seed used for generating the networks to be a random effect, since there might be some dependencies between the network sequences simulated using the same seeds. Additionally, those simulated from different seeds may be of a different level of difficulty to distinguish. The fitted probability of successful detection, as well as the estimates of the coefficients for the GLMM is shown in Figs 10 and 11, respectively. The ANOVA table associated with the fitted model is provided in Table 3. The results show that the rate of detection is significantly affected by *B* (number of particles), $t_{M}^{\prime }$ (how much percolation curve is observed for testing), but not *N*. And it’s slightly easier for ER sequences to be identified as such than for PR sequences. Also, our testing algorithm works best when taking the percolation curve up to $t_{M}^{\prime }=1.34$ for testing, where the GCC starts to form into a single giant cluster incorporating most of the nodes. Having either too little or too much of the curve will impair the accuracy.

**Table 3:** ANOVA table for the first simulation study.

	Chisq	Df	Pr(>Chisq)
N	0.52	2	0.7728
scaled_t	18.88	3	0.0003
process	7.68	1	0.0056
B	14.29	2	0.0008

We also conduct a second simulation study to evaluate the effect of observation rate *κ* on the rate of detection for ER and PR using Bayes factor, which shows that rate of detection significantly increases by increasing *κ* from 0.5 to 1, but stops to significantly increase as *κ* further increases. Details of this simulation study is provided in section 6.2 of S1 Text.

In conclusion, our proposed testing framework can effectively distinguish between different percolation models using an observed sequence of noisy networks under proper settings. The size of the network does not affect the ability to discern between the two percolation regimes and such ability increases as *B* (the number of particles) and *κ* (the observation rate) increases. This coincides with our intuition that a better particle approximation for the latent distribution, as well as a more informed signal, would help in distinguishing between percolation models.

### Results for in vivo data during human seizure

In this section, we show application results of our methodology to functional dynamic networks constructed using electrocorticography data recorded from a human subject during three seizures.

#### Automatic segment finder

Before we apply our estimation and testing framework to the constructed networks, we need first choose segments of the network time series consistent with percolation. Previous work [8, 9] suggests sudden increases in the size of connected functional network communities occur at multiple times during a seizure (e.g., near seizure onset and termination), making nontrivial the choice of an appropriate time segment from the overall non-stationary signal. We developed a simple, automatic procedure for this purpose, consisting of roughly the following steps: 1) Select intervals of time that only contain network evolution in the regions of interest (ROIs) corresponding to periods of seizure evolution on clinical review of the data, as determined by a board-certified epileptologist (CJC) from the time intervals in which large dynamic communities appear (for details of the dynamic community detection procedure on network time series see [9]). 2) Identify segments in the ROIs where both the size of GCC and density ramp up over time and use them for testing. The details of this procedure are provided in section 3 of S1 Text. Applying this procedure to the first seizure, we identify three segments, as shown in the bottom row of Fig 4 (bold curves). Results for the other two seizures are shown in section 7 of S1 Text.

#### Estimation and statistical testing

Having selected time segments of dynamic functional network evolution during the seizures, we now apply the statistical testing procedure to infer the percolation regime—ER or PR—for each segment. We take each chosen segment from the automatic segment finder as input for the statistical testing procedure under our RG-HMM framework, with *B* = 500, 000. For each seizure, we report the mean and standard deviation of parameter estimates under the two percolation models, the likelihood evaluated at those estimates, and the estimated Bayes factors in log scale. The results for one of the chosen segments in seizure 1 are given in Table 4; see section 7 of S1 Text for results from other seizures.

**Table 4:** Mean and standard deviation of parameter estimates for ER/PR process, log-likelihood estimates and Bayes factor (log scale) estimate based off of 10 trials for seizure 1 (first bold segment) on the left brain hemisphere.

	$\hat{p}$	$\hat{q}$	$\hat{\gamma }$	$\hat{\alpha }$	$\hat{\beta }$	*loglik*
ER	0.404 (0.112)	0.248 (0.075)	10.075 (1.423)	0.068 (0.0005)	0.435 (0.035)	-5636.822 (5.942)
PR	0.468 (0.156)	0.163 (0.072)	9.819 (1.512)	0.068 (0.0004)	0.464 (0.032)	-5647.120 (6.850)
log(BF)	10.297 (9.468))					

The Bayes factors in log scale are positive for all segments from seizure 1 (10.3, 31.7 and 62.0, respectively); all segments from seizure 2 (51.0, 105.8 and 7.1, respectively); and 3 of 4 segments from seizure 3 (10.9, 61.5, 15.1 and −5.0, respectively). We conclude that Erdos-Renyi (ER) percolation is better supported by the data for the entire course of seizure 1 and seizure 2, as well as the early ictal stages of seizure 3, while product-rule (PR) percolation is better supported for the late ictal stage of seizure 3.

These preliminary results suggest that both types of percolation can occur in the dynamic functional networks of human seizures. For this patient, though each seizure begins in the same focal location with the same recruitment patterns of type ER, the recruitment patterns differ preceding seizure termination, which are of type ER for seizure 1,2 and type PR for seizure 3. Clinically, seizure 3 differs from the other two seizures; seizure 3 remains spatially focal, while seizures 1 and 2 continue to propagate and only terminate after recruiting the entire brain. These preliminary results suggest a different network dynamic for the two clinical seizure types. Whether local PR percolation (here, in the left hemisphere) correlates with focal seizure termination and prevents generalization (here, to the right hemisphere) requires further investigation. We note that identification of percolation type may also suggest different treatment strategies. For example, to prevent PR percolation, the growing subnetworks that emerge could be separately targeted and prevented from joining. Alternatively, to prevent ER percolation, effective treatment may require preventing the expansion of the emerging single connected component.

In need of understanding how well the model fits the data, we conduct a goodness-of-fit assessment of the ER/PR percolation model on the seizure data through a Monte Carlo approach. This approach involves generating networks from the fitted model and comparing with the observed networks on several network summary statistics, which is heavily used for assessing goodness-of-fit for network models, especially exponential random graph models (ERGMs) [22]. Based on this goodness-of-fit assessment, we found that, overall, the ER/PR percolation model fits the seizure data decently well. Details of this goodness-of-fit test are provided in section 9 of S1 Text.

It is also of interest to assess the robustness of our results to the network construction and the segment identification. We thus conduct a robustness check on our results to shed some light on how our findings may vary with the threshold used in defining network edges. We construct a new set of network time series data using a different threshold value and rerun the testing procedure on the resulting networks for each of the three seizures. From this robustness check of limited scope, we observe that our findings on percolation regimes for seizures 1 and 2 are quite robust to the network constructions. However, it seems that for seizure 3, the finding on the evidence of PR percolation at the late ictal stage is sensitive to the network construction, caused by the ROI and segment identification being sensitive to the network construction in this seizure. Details on this robustness check are provided in section 8 of S1 Text.

## Discussion

We develop a class of random graph hidden Markov models (RG-HMMs) for characterizing percolation regimes in noisy, dynamically evolving networks in the presence of edge birth and edge death, under which exponential waiting times are used to model the time between the addition or removal of an edge. We present an EM algorithm with a particle filtering and sample path simulation scheme for estimating model parameters from a sequence of noisy networks observed only at a longitudinal subsampling of time points (i.e., not at every time step of edge change). We also provide a framework for statistical testing of competing hypotheses of percolation regimes, which involves calculation of the likelihoods evaluated at optimal estimates under each hypothesis, given the observed networks. We now provide discussions on the simulation and application results respectively, along with some directions in which our work can be extended, as well as challenges and opportunities for future work.

### Discussion on simulation results

In the simulation results, it may sound counter-intuitive that the ability to discern between the two percolation regimes is not monotonically increasing as a longer duration of the percolation curve is taken for testing considering there is more information. It is actually not surprising as the signal distinguishing ER and PR mostly lies in the GCC growing stage where small connected components start to form before joining into a single giant cluster, which is also when the Achlioptas product rule can be effectively applied to the edge addition/deletion process. Once all nodes become connected, each node will belong to the same connected component, the product rule will no longer affect edge addition/deletion, and edges will be added and deleted randomly in the PR process just like in the ER process. Although taking more of the percolation curve seems to bear more information, it actually dilutes the most effective signal in the GCC growing stage and deteriorates the ability to discern.

From our numerical experience, bias in MLE seems not to compromise the ability to discern between competing models. It is quite important to note that although a longer observational sequence is needed for accurate estimation of parameters, the ability to discern does not necessarily increase by observing more of the percolation curve. Instead, it can be increased by improving the signal-to-noise ratio through more frequent observations on network changes. In terms of physiological time series in potential applications, this means obtaining high-resolution network data through either construction or observation with a high sampling rate—the feasibility of which may vary with construction methods and/or measurement modality.

### Discussion on application results

#### Toward results interpretation

Given that recent work in network science, as it pertains to epilepsy, has shown an explosive density increase in functional connectivity networks in epilepsy patients during seizure onset, our work has the potential to be quite impactful for clinical neuroscience. To demonstrate the application to epileptic seizures, we apply our framework to assess, at a minimum, whether ER or PR percolation is better supported by the data for these particular seizures. We note that epilepsy is a complex and still incompletely understood disease. While it would be naive to expect that support for one type of percolation regime over another gleaned from our functional connectivity networks would offer significant insights into the etiology of this disease, we nevertheless are optimistic that results obtained through the application proposed here will be sufficiently suggestive as to inspire additional constructive thinking about the period of seizure onset, which may lead to improvements in treatment and disease management.

However, there are limitations when interpreting a single test result. Our simulation result, under the favorable scenario where the largest number of particles and the optimal amount of the curve are taken for testing, shows that the successful detection rate for ER ranges from roughly 75% to 81%, and for PR from 72% to 78%. Given this, all testings on percolation regimes are subject to false positive/negative results. Although the value of Bayes factor itself is a confidence measure on how certain the data supports one model better than the other, a goodness-of-fit test for the ER/PR percolation model on the seizure data, as illustrated in section 9 of S1 Text, can be used as another way to check the credibility of the inferred percolation process. Nevertheless, we must interpret each single testing result cautiously given the uncertainties.

One may apply the testing framework to multiple seizure instances on a single subject (if the data is available) to improve upon estimation of parameters and percolation type detection rate. Within a patient, seizures often (but not always) display consistency (e.g., in clinical manifestations, in electrophysiological features, in spatiotemporal dynamics, etc.). We would, therefore, expect to find similarities across a patient. During invasive monitoring for epilepsy surgery, multiple seizures often are recorded. Finding all PR or all ER across a patient’s observed seizures (within a particular seizure type—e.g., with/without secondary generalization) would add confidence that the estimated regime is correct.

Performing a meta-analysis would be another way to boost confidence in the interpretation of the results. For example, we may apply our testing framework to seizure data from a group of patients and perform a meta-analysis to aggregate and contrast the findings from single seizures to identify patterns and interesting relationships that may come to light by comparing the test results against patient phenotype information. This phenotype information may include medical classification of severity of the disease, putative locations of seizure emergence and other clinically relevant measurements. Such meta-analysis may help the current limitation in interpreting the single testing result since the uncertainties in the findings from each single testing result will be regarded as noise, and the overall effect will be revealed from the statistical analysis.

#### Toward goodness-of-fit of ER/PR percolation model

Despite the goodness-of-fit test for the ER/PR percolation model on the seizure data illustrated in section 9 of S1 Text, where we find that overall the model fits the data decently well, an interesting question is whether there is a better model out there that fits the data better.

There is almost certainly a better model that exists, of course, but our goal was not to test every percolation and biophysical model. Instead, we have focused on developing the first version of a fairly generalizable paradigm for testing different percolation mechanisms, corresponding to the sudden emergence of functional connectivity during human seizures.

In the proposed model, we consider two canonical random graph models i.e., Erdos-Renyi and product-rule, representing two prototypical extremes of percolation. Notably, however, there is a wide spectrum of other random graph models, displaying a variety of percolation behaviors (e.g., [23], Fig 1). Our work may be extended to these other percolation regimes, given that our proposed RG-HMM is a flexible framework which may subsume a variety of random graph models. More generally, it would be of interest to extend our methods to fitting increasingly more sophisticated models, particularly mechanistic models informed by biophysical understanding in neuroscience. However, we suspect if they are not sufficiently close to percolation models, then entirely new modeling approaches may be needed.

#### Toward challenges and opportunities

Although we discovered from the simulation study that the ability to discern between the two percolation regimes maximizes when taking a certain duration of percolation curve for testing, we found it challenging to apply this learning to the real-world seizure data. The real seizure data contains a non-stationary signal in the sense that multiple percolation processes with various edge changing rates and durations may happen in one seizure at different places. Extracting a stationary signal to test for each possible percolation process in one seizure is already a challenging task. Identifying the optimal length of duration for testing is even harder and sometimes not feasible when the percolation process ends before the single giant connected component appears. Of course, visual inspection may be one way to go, but it is subjective and not efficient when testing for multiple seizures. So the practice we adopt in this paper is an automatic approach to finding segments for testing, which applies the same criteria across all seizure data. Improving this segment finding strategy to optimize the testing performance for each segment would be of interest for future research.

Lastly, although we have chosen to focus our attention on epileptic seizures, our class of models and our framework for parameter estimation and testing have the potential to be used outside of the realm of seizure data to better understand the emergence of organized structure in many other dynamic systems. With percolation theory omnipresent across a vast range of applications such as social networks, traffic networks, infectious disease networks, amino acid networks, and even understanding the spread of forest fires, computer viruses, etc. ([24–27]), our hope is that our methods may be used to better understand network dynamics across a wide range of fields.

## Supporting information

S1 Text Supporting information for “Distinguishing between different percolation regimes in noisy dynamic networks with an application to epileptic seizures”. (PDF)
