# Supplementary material for: Distinguishing between different percolation regimes in noisy dynamic networks with an application to epileptic seizures
Source: PLoS Comput Biol. 2023 Jun 16;19(6):e1011188. doi: 10.1371/journal.pcbi.1011188 (PMC10310035; doi:10.1371/journal.pcbi.1011188)
Supplement: S1 Text — (PDF) [file pcbi.1011188.s001.pdf]

Supporting Information for "Distinguishing between different percolation regimes in noisy dynamic networks with an application to epileptic seizures"

Xiaojing Zhu[1], Heather Shappell[1], Mark A. Kramer,
Catherine J. Chu, Eric D. Kolaczyk*
kolaczyk@bu.edu

Contents

1 Details on ML Estimation Regime for RG-HMM **2**
1.1 Complete-data Log-likelihood 3
1.2 EM for Incomplete-data 3
1.3 Particle Filtering 4
1.4 Simulating the Sample Path 6
1.5 Putting it Together - the EM Algorithm 7

2 Details on Computing Marginal Probability of Observations **9**
2.1 Derivation of Recurrence Relation in Equ (5) in the Main Text 9
2.2 A Forward Algorithm with Particle Filtering 9

3 Details on Automatic Segment Finder **10**

4 Model Identifiability for RG-HMM **11**

5 Asymptotic Properties of MLE for RG-HMM **13**
5.1 Asymptotic normality 13
5.2 Proof of Theorem 5.1 13

6 Supplementary Simulation Results **17**
6.1 Numerical Simulation Results for Estimation 17
6.2 Testing Results 18

7 Supplementary Application Results **19**

8 Robustness of Results to Network Construction and Segment Identification **22**

9 Goodness of Fit for RG-HMM **26**

Appendices **29**

*To whom correspondence should be addressed.
[1]These authors contributed equally to this work.

A PMF of Feasible Sample Path 30

B Transition Probability for Percolation Models 31

C Proof of Missing Information Principle 32

This supplement contains nine sections. Section 1 and Section 2 provide details for the two subroutines mentioned in the main text for computing the Bayes factor. Section 3 provides details on the automatic segment finder. Section 4 shows a proof for the model identifiability. Section 5 provides the asymptotic normality result for the MLE in our proposed RG-HMM as M (the number of observation time points) goes to infinity. Section 6 and 7 provide supplementary simulation and application results, respectively. Section 8 provides a robustness check on the testing results to network construction and segment identification. Section 9 presents a goodness-of-fit assessment of the ER/PR percolation model to the seizure data through a graphical test.

1 Details on ML Estimation Regime for RG-HMM

Although our ultimate goal is testing for two competing hypotheses of percolation regimes, learning the RG-HMM from a sequence of noisy networks observed only at a longitudinal subsampling of times is a necessary precursor and of independent interest.

Throughout this section, we assume $\alpha < 0.5$ and $\beta < 0.5$. Under such an assumption, the parameters in the model are identifiable. (A proof is provided in section 4.) We present an Expectation-Maximization (EM) algorithm for estimating the parameters in an RG-HMM (i.e. $p, q, \gamma, \alpha, \beta$) with a given sequence of noisy networks observed only from a longitudinal subsampling of time points, i.e. $\boldsymbol{g}^{\star}_{1:M}$. We assume that the network in the hidden layer changes faster than the observing rate so that there are likely many non-observed small changes occurring between the consecutive observation times, as is typically the case for functional connectivity networks used in the study of epilepsy.

Estimation is done conditional on the latent state $W(t_1) = 1, G(t_1) = g^{\star}(t_1)$ at the first observation time t_1. As in [1], this has the advantage that no initial distribution assumption is needed for the latent Markov chain, and the estimated parameters refer exclusively to the dynamics of the network.

The algorithm consists of an E-step, wherein we calculate the expected value of the complete data log-likelihood $l(\alpha, \beta, \gamma, p, q) = \log f(\boldsymbol{W}_{2:M}, \boldsymbol{G}_{2:M}, \boldsymbol{g}^{\star}_{2:M})$, with respect to the distribution of unknown latent variables $\boldsymbol{W}_{2:M}, \boldsymbol{G}_{2:M}$, given the observed networks $\boldsymbol{g}^{\star}_{1:M}$ and the current parameter estimates. We then maximize the expected log-likelihood in the M-step.

The E-step is non-trivial in that there are two levels of unknown latent variables in our model: we do not know the true networks and binary variables $W(t_m), G(t_m)$ at the observation times, nor do we know the intermediate path that connects one true state $W(t_{m-1}), G(t_{m-1})$, to the next $W(t_m), G(t_m)$. EM is standard for parameter estimation in general HMMs. However, we face some unique challenges: (1) direct calculation of the expectation is computationally infeasible due to the enormously large state space of size $2^{\binom{N}{2}}$; (2) the observed-data likelihood is difficult to calculate due to the unobserved changes occurring between consecutive observation times. Particle filtering and augmented data sampling using MCMC are used to tackle these challenges.

1.1 Complete-data Log-likelihood

For the complete data scenario, we assume we have samples $\boldsymbol{w}_{2:M}, \boldsymbol{g}_{2:M}$ for the hidden layer. Then the complete data log-likelihood for the RG-HMM, conditional on $W(t_1) = 1, G(t_1) = g^\star(t_1)$, can be written as

$$\begin{aligned} l^\star(\alpha, \beta, p, q, \gamma) &= \log f(\boldsymbol{w}_{2:M}, \boldsymbol{g}_{2:M}, \boldsymbol{g}^\star_{2:M}) \\ &= \sum_{m=2}^{M} \log f_{\alpha,\beta}(g^\star_m | g_m) + \sum_{m=2}^{M} \log f_{p,q,\gamma}(w_m, g_m | w_{m-1}, g_{m-1}), \end{aligned} \tag{1.1}$$

where g_m, $g^\star_m$ is the abbreviation for $g(t_m)$ and $g^\star(t_m)$, respectively, α is the type I error rate, β is the type II error rate, p and q are birth and death rate, and γ is the rate parameter for the continuous-time percolation model in which the hidden chain in RG-HMM was embedded.

The first term in (1.1) is based on the conditional distribution of the observed network given the truth, which takes the form

$$f_{\alpha,\beta}(g^\star_m | g_m) = \alpha^{c_m}(1-\alpha)^{d_m}\beta^{b_m}(1-\beta)^{a_m}, \tag{1.2}$$

where a_m, b_m, c_m, d_m are counts corresponding to type I and type II errors in the error process from g_m to $g^\star_m$ at observation time t_m. Details are shown in Table 1.1.

Table 1.1: **Counts corresponding to type I and type II errors**

True/Observed	E	E^c
E	a	b
E^c	c	d

The second term in (1.1) is the log-likelihood of the embedded discrete-time Markov chain in the hidden layer, which cannot be calculated in closed form due to the non-observed small changes occurring between consecutive observation times in the hidden layer. Therefore, we consider the log-likelihood for augmented data in order to obtain a more easily computed likelihood. The details are as follows.

Augmented data. The data augmentation in the hidden layer can be done for each period (t_{m-1}, t_m), $m = 2, ..., M$. Consider period t_1 to t_2. We assume there are R_1 transition time points between t_1 and t_2, denoted as $\tau_1, ..., \tau_{R_1}$, which are ordered increasingly. Setting $\tau_0 = t_1$, we have $t_1 = \tau_0 < \tau_1 < \tau_2, ..., < \tau_{R_1} \le t_2$. The sample path from $G(t_1)$ to $G(t_2)$ is characterized by

$$\boldsymbol{S}_1 = (G(\tau_1), G(\tau_2), ..., G(\tau_{R_1})), \quad \boldsymbol{V}_1 = (W(\tau_1), W(\tau_2), ..., W(\tau_{R_1})), \tag{1.3}$$

which specify the sequence of unobserved change that brings the network from $G(t_1), W(t_1)$ to $G(t_2), W(t_2)$. Thus, the feasible sample path $\boldsymbol{S}_1$, $\boldsymbol{V}_1$ from $G(t_1)$ to $G(t_2)$ should satisfy two conditions: 1) $G(\tau_j), G(\tau_{j+1})$ differ by only one edge for $j = 1, ..., R_1 - 1$; and 2) $G(\tau_{R_1}) = G(t_2)$, $W(\tau_{R_1}) = W(t_2)$. The details of the probability mass function of a sample path $\boldsymbol{S}_1, \boldsymbol{V}_1$ conditional on $G(t_1), W(t_1)$ can be found in Appendix A.

1.2 EM for Incomplete-data

The goal is to find $\arg\max_\Gamma f(\boldsymbol{g}^\star_{2:M} | G_1 = g^\star_1, W_1 = w_1)$, where $\Gamma = (p, q, \gamma, \alpha, \beta)$.

E-step The expected value of the complete data log-likelihood with respect to the true networks and true binary variables $\boldsymbol{G}_{2:M}, \boldsymbol{W}_{2:M}$, given the observed networks $\boldsymbol{g}^\star_{1:M}$ and current parameter estimates $\Gamma^{(k-1)}$ is

$$Q(\Gamma; \Gamma^{(k-1)}) = E\left[\log f(\boldsymbol{g}^\star_{2:M} | \boldsymbol{G}_{2:M}) \middle| \boldsymbol{g}^\star_{1:M}, \Gamma^{(k-1)}\right] + E\left[\log f(\boldsymbol{W}_{2:M}, \boldsymbol{G}_{2:M}) \middle| \boldsymbol{g}^\star_{1:M}, \Gamma^{(k-1)}\right]. \tag{1.4}$$

M-step The first term in (1.4) can be easily maximized since it has a closed-form log-likelihood in (1.2), which yields the following:

$$\hat{\alpha} = \frac{E[C|\boldsymbol{g}^\star_{1:M}, \Gamma^{(k-1)}]}{E[C+D|\boldsymbol{g}^\star_{1:M}, \Gamma^{(k-1)}]}, \quad \hat{\beta} = \frac{E[B|\boldsymbol{g}^\star_{1:M}, \Gamma^{(k-1)}]}{E[A+B|\boldsymbol{g}^\star_{1:M}, \Gamma^{(k-1)}]}. \tag{1.5}$$

The formula for $\hat{\alpha}$ is the expected number of false edges in the observed network divided by the expected number of non-edges in the true network, given the observed networks $\boldsymbol{g}^\star_{1:M}$ and current parameter estimates $\Gamma^{(k-1)}$. The formula for $\hat{\beta}$ is the expected number of false non-edges in the observed network divided by the expected number of edges in the true network, given the observed networks $\boldsymbol{g}^\star_{1:M}$ and current parameter estimates $\Gamma^{(k-1)}$.

The second term in (1.4) can be maximized by setting the score function to be zero,

$$E\left[S(\boldsymbol{\theta}; \boldsymbol{W}_{2:M}, \boldsymbol{G}_{2:M})\middle|\boldsymbol{g}^\star_{1:M}, \Gamma^{(k-1)}\right] = 0, \tag{1.6}$$

where $\boldsymbol{\theta} = [p, q, \gamma]$, $S(\boldsymbol{\theta}; \boldsymbol{w}_{2:M}, \boldsymbol{g}_{2:M}) = \frac{\partial}{\partial \boldsymbol{\theta}} \log f(\boldsymbol{w}_{2:M}, \boldsymbol{g}_{2:M})$ is the partial data score function, which cannot be calculated in closed form. Note that the score function for the augmented data is in closed form and by the Missing Information Principle used in [1], we have that

$$S(\boldsymbol{\theta}; \boldsymbol{w}_{2:M}, \boldsymbol{g}_{2:M}) = E\big[S(\boldsymbol{\theta}; \boldsymbol{w}_{2:M}, \boldsymbol{g}_{2:M}, \boldsymbol{S}, \boldsymbol{V})\big|\boldsymbol{W}_{2:M} = \boldsymbol{w}_{2:M}, \boldsymbol{G}_{2:M} = \boldsymbol{g}_{2:M}\big]. \tag{1.7}$$

In other words, the partial data score function can be calculated by taking the expectation of the total data score function. The proof is provided in Appendix C. Therefore, solving (1.6) yields the following closed-form solution for γ, p and q,

$$\hat{\gamma} = \frac{1}{t_M - t_1} E\left[E\left[\sum_{m=2}^{M} R_{m-1}\middle|W_{2:M} = \boldsymbol{w}_{2:M}, \boldsymbol{G}_{2:M} = \boldsymbol{g}_{2:M}\right]\middle|\boldsymbol{g}^\star_{1:M}, \Gamma^{(k-1)}\right] \tag{1.8}$$

$$\hat{p} = \frac{E\left[E\left[\sum_{m=2}^{M}\sum_{r=1}^{R_{m-1}} I\{W(\tau^{m-1}_{r-1}) = 0, W(\tau^{m-1}_{r}) = 1\}|W_{2:M} = \boldsymbol{w}_{2:M}, \boldsymbol{G}_{2:M} = \boldsymbol{g}_{2:M}\right]\middle|\boldsymbol{g}^\star_{1:M}, \Gamma^{(k-1)}\right]}{E\left[E\left[\sum_{m=2}^{M}\sum_{r=1}^{R_{m-1}} I\{W(\tau^{m-1}_{r-1}) = 0\}|W_{2:M} = \boldsymbol{w}_{2:M}, \boldsymbol{G}_{2:M} = \boldsymbol{g}_{2:M}\right]\middle|\boldsymbol{g}^\star_{1:M}, \Gamma^{(k-1)}\right]} \tag{1.9}$$

$$\hat{q} = \frac{E\left[E\left[\sum_{m=2}^{M}\sum_{r=1}^{R_{m-1}} I\{W(\tau^{m-1}_{r-1}) = 1, W(\tau^{m-1}_{r}) = 0\}|W_{2:M} = \boldsymbol{w}_{2:M}, \boldsymbol{G}_{2:M} = \boldsymbol{g}_{2:M}\right]\middle|\boldsymbol{g}^\star_{1:M}, \Gamma^{(k-1)}\right]}{E\left[E\left[\sum_{m=2}^{M}\sum_{r=1}^{R_{m-1}} I\{W(\tau^{m-1}_{r-1}) = 1\}|W_{2:M} = \boldsymbol{w}_{2:M}, \boldsymbol{G}_{2:M} = \boldsymbol{g}_{2:M}\right]\middle|\boldsymbol{g}^\star_{1:M}, \Gamma^{(k-1)}\right]}. \tag{1.10}$$

where R_{m-1} is the total number of transition times between t_{m-1} and t_m in the augmented sample path and τ^{m-1}_r is the r-th transition time between t_{m-1} and t_m.

1.3 Particle Filtering

The expectation step of our E-M algorithm is difficult to calculate. Since the state space for latent variables is prohibitively large, it is not feasible to directly calculate the conditional expectations in the aforementioned MLEs by summing over all possible network sequences, binary variable sequences and sample paths in the hidden layer. Instead, we propose to estimate those conditional expectations in (1.5), (1.8), (1.9), (1.10) by sampling from the state space of latent variables.

Note that there are two levels of the expectation in (1.8), (1.9), and (1.10). The first is the outer expectation, which is taken with respect to the conditional distribution of $\boldsymbol{W}_{2:M}, \boldsymbol{G}_{2:M}$ in the hidden layer given the observed networks $\boldsymbol{g}^\star_{1:M}$, which we propose to sample from by a particle

filtering based sampling scheme described in [2] (i.e. a Sequential Monte Carlo method). This sampling scheme involves first approximating $f(w(t_m), g(t_m) \,|\, \boldsymbol{g}^\star_{1:m-1}, \boldsymbol{\Gamma}^{(k-1)})$ with B particles (i.e. samples for the true network and binary variable pair) at each observation time t_m, then tracing the ancestral lines, or particle paths, for the particles as samples from $f(\boldsymbol{w}_{2:M}, \boldsymbol{g}_{2:M} \,|\, \boldsymbol{g}^\star_{1:M}, \boldsymbol{\Gamma}^{(k-1)})$. The B particles at each observation time reduces the space from which we will sample from for the particle paths, thus allowing us to effectively sample some number Ψ network and binary variable sequences that are more likely to generate the observed networks in the error process. A visual representation for this procedure is provided in Fig 1.1.

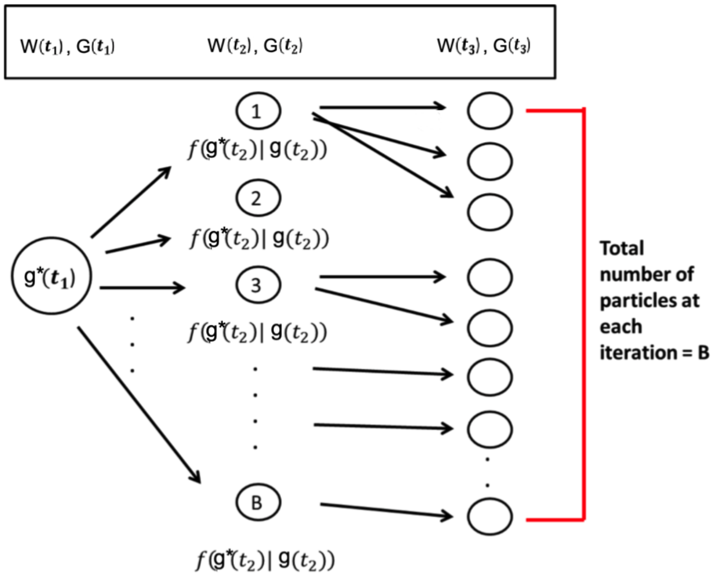

Figure 1.1: **Particle filtering sampling scheme.** B particles are sampled at each observation moment following a two-stage process: 1) particles are sampled at the previous moment with probability proportional to the conditional distribution of the true network given the observed network; 2) then a true network and binary variable at the current observation moment, i.e. a new particle, is simulated starting from one of the sampled particle in step 1, according to ER/PR process. Ψ ancestral lines are obtained by tracing back the ancestral lines for Ψ particles at time t_M, sampled with probability proportional to the conditional distribution of the observed network given the true network at time t_M.

The particle filtering procedure is as follows:

1. Start from the first observed network $g^\star(t_1)$. Set $G(t_1) = g^\star(t_1)$ and $W(t_1) = 1$.

2. To create B number of particles at observation time t_2, denoted by $\{w^b(t_2), g^b(t_2)\}_{b=1}^B$, repeat the following for $b = 1, \cdots, B$.

   (a) Start from $W(t_1) = 1, G(t_1) = g^\star(t_1)$ and simulate according to a continuous time ER/PR process up until time $t_2 - t_1$. Then set $w^b(t_2), g^b(t_2)$ equal to the current binary variable value and network in the simulated chain.

3. For $m = 2, \cdots, M-1$, repeat the following steps.

   (a) For each one of the simulated pairs in $\{(w^b(t_m), g^b(t_m))\}_{b=1}^B$, calculate the conditional probability $p_b := P(G^\star(t_m) = g^\star(t_m) \big| G(t_m) = g^b(t_m))$ with current parameter estimates.

   (b) To create B number of particles at time t_{m+1}, repeat the following for $b = 1, \cdots, B$.

      i. Sample an index η from $1, 2, ..., B$ with probability proportional to $p_1, p_2, ..., p_B$.

ii. Start from $W(t_m) = w^\eta(t_m), G(t_m) = g^\eta(t_m)$ and simulate according to a continuous time ER/PR process up until time $t_{m+1} - t_m$. Then set $w^b(t_{m+1}), g^b(t_{m+1})$ equal to the current binary variable value and network in the simulated chain.

4. To obtain Ψ samples from the conditional distribution of $\boldsymbol{W}_{2:M}, \boldsymbol{G}_{2:M}$ given the observed networks $\boldsymbol{g}^\star_{1:M}$, we sample Ψ ancestral lines, or particle paths, denoted by $\{\boldsymbol{w}^\psi_{2:M}, \boldsymbol{g}^\psi_{2:M}\}_{\psi=1}^{\Psi}$ with the following procedure. We first calculate the conditional probability at the last observation time $p_b := P(G^\star(t_M) = g^\star(t_M) \big| G(t_M) = g^b(t_M))$ with current parameter estimates, for $b = 1, \cdots, B$, then repeat the following for $\psi = 1, ..., \Psi$.

   (a) Sample an index η_M from $1, 2, ..., B$ with probability proportional to $p_1, p_2, ..., p_B$.

   (b) Trace back the ancestral line for the η_M-th particle, i.e. $(w^{\eta_M}(t_M), g^{\eta_M}(t_M))$, at time t_M. In other words, identify the ancestral particle index for the η_M-th particle at time t_M, and call it η_{M-1}, then identify the ancestral particle index for η_{M-1}-th particle at time t_{M-1}, and call it η_{M-2}, and continue to do this backward for η_m-th particle at time t_m, $m = M-2, \cdots, 2$. Note that at each observation moment, each particle has exactly one parent. Then set the ancestral line, i.e. $\{(w^{\eta_m}(t_m), g^{\eta_m}(t_m))\}_{m=2}^{M}$ as one sample from the conditional distribution of $\boldsymbol{W}_{2:M}, \boldsymbol{G}_{2:M}$, given $\boldsymbol{g}^\star_{2:M}$.

The particle filtering allows us to sample Ψ network and binary variable sequences that are more likely to generate the observed networks in the error process. It generates B possible latent variables at each observation time, thus effectively reducing the space from which we will sample from for the particle paths. It also allows us to sample true network and binary sequences without actually having the transition probabilities in closed form.

1.4 Simulating the Sample Path

The second level of the expectation in (1.8), (1.9), and (1.10) is the inner expectation, which is taken with respect to the conditional distribution of sample path $\boldsymbol{S}, \boldsymbol{V}$ that connects the given true sequences $\boldsymbol{w}_{2:M}, \boldsymbol{g}_{2:M}$ at the observation times in the hidden layer. We sample from this conditional distribution by simulating paths using an MCMC method. We draw upon the work of [1] where draws are generated by the Metropolis-Hastings algorithm, using a proposal distribution consisting of small changes in a sample path that brings one network to the next.

Specifically, for each $m = 2, \cdots, M$, we need to generate a sample path $\boldsymbol{s}_{m-1}, \boldsymbol{v}_{m-1}$ that brings $(w(t_{m-1}), g(t_{m-1}))$ to $(w(t_m), g(t_m))$. The probability function of the sample path, conditional on $(w(t_{m-1}), g(t_{m-1}))$ is given in (A.4). To simplify the notation, denote the sample path and its conditional probability function by $\boldsymbol{u}$ and $p(\boldsymbol{u})$, respectively. The acceptance ratio in the Metropolis-Hasting algorithm for a current state $\boldsymbol{u}$ and a proposed state $\tilde{\boldsymbol{u}}$ is

$$\min\{1, \frac{p(\tilde{\boldsymbol{u}})q(\boldsymbol{u}|\tilde{\boldsymbol{u}})}{p(\boldsymbol{u})q(\tilde{\boldsymbol{u}}|\boldsymbol{u})}\} \tag{1.11}$$

where $q(\tilde{\boldsymbol{u}}|\boldsymbol{u})$ is the proposal distribution under which a new candidate sample path $\tilde{\boldsymbol{u}}$ is proposed by making small changes in the current sample path $\boldsymbol{u}$. The small changes consist of three operations: paired deletion, paired insertion and permutation. Paired deletion involves removing a pair of same edge changes in sample path. For example, if sample path $\boldsymbol{u}$ consists of adding an edge at one time and deleting the same edge after, then a new sample path $\tilde{\boldsymbol{u}}$ can be obtained by removing such pair of same edge changes from $\boldsymbol{u}$ since the two edge changes offset each other producing no change on the network. Similarly, paired insertion involves adding a pair of same edge changes in a current

sample path, and permutation involves permuting all edge changes in a current sample path. All of these operations are done in such a way that the proposed new sample path $\tilde{\boldsymbol{u}}$ is still a feasible path with only one edge change at a time connecting the two networks at the consecutive observation times in the hidden layer. More discussion of this MCMC procedure can be found in [1].

1.5 Putting it Together - the EM Algorithm

The EM algorithm resulting from the combination of all elements described in the previous subsections, for maximum likelihood estimation of the parameters p, q, γ, α, β in our RG-HMM, is shown as Algorithm 1.

The convergence criteria we have used in practice is $\frac{||\hat{\Gamma}^{k+1}-\hat{\Gamma}^{k}||_2}{||\hat{\Gamma}^{k}||_2} < 0.10$. Based on our numerical experience, the proposed EM algorithm usually converges within 5 iterations when all parameters are initialized as 0.5. Less iterations will be needed if parameters are initialized somewhere close to the true values. The choice of B (i.e. the number of particles sampled at each observation moment), the choice of Ψ (i.e. the number of true network series to sample for the maximization of error rates α and β) and the choice of H (i.e. the number of true network series to sample for the maximization of p, q, γ) may vary depending on the size of network and the number of observation time points. Our recommended starting point is to set $B = 50,000$, $\Psi = 40,000$ and $H = 10$. It is important to keep in mind that the maximization of p, q, γ involves a computationally expensive MCMC procedure. Setting H to a small number helps efficiently reduce the computational burden without compromising much on the accuracy of parameter estimates, in our experience.

In our EM algorithm, time complexity is $\mathcal{O}(BM)$ for the particle filtering step, $\mathcal{O}(HM)$ for the MCMC step, and $\mathcal{O}(\Psi M)$ for sampling Ψ number of ancestral lines. Although the estimation procedure scales linearly in B, M and H, it is still a computationally expensive procedure for large networks because simulating the true networks in the hidden layer involves sampling from edges or non-edges of a N-node network, which scales quadratically in N using standard techniques.

Our experience in the reported simulations and in working with empirical data sets is that the algorithm converges well. However, it is time-consuming, especially for larger networks requiring more particles for approximation. For example, when working with a 30 node network with 30 observation time points and 50,000 particles, one testing procedure, including ML estimations under two hypotheses and the calculation of Bayes factor, takes approximately 1 hour to run on a high performance Linux computing cluster using 4 cores with appropriate use of parallelization techniques for speedups. If we multiply the number of particles by 10, i.e. $B = 500,000$, the algorithm will take approximately 10 hours. In principle, the larger B is, the more accurate estimates will be. However, it comes with the trade-off between computational time and accuracy. While it is feasible to run our algorithm on even larger networks under manageable time with a relatively small particle size, we must interpret the results cautiously. In such cases, we recommend running multiple testing trials and taking an average of the approximated Bayes factor to reduce the high variance caused by a relatively small number of particles.

In the E-step of our EM algorithm, we apply particle filtering to approximate the expected log-likelihood of the complete-data. Although a central limit theorem [3] exists for the particle estimate of the likelihood, which assures the asymptotic unbiasedness of the approximation in E-step as $B \to \infty$, it is still difficult to theoretically investigate the convergence property of our EM algorithm with the rather complicated likelihood function in the RG-HMM. Therefore, we resort to simulation to evaluate the empirical performance of our EM in the main text.

Algorithm 1: EM Algorithm for MLEs

Input: Network snapshots: $\boldsymbol{g}^\star = \big(g^\star(t_1), g^\star(t_2), ..., g^\star(t_M)\big)$, B, H, Ψ

Output: $\hat{\Gamma} = [\hat{\alpha}, \hat{\beta}, \hat{p}, \hat{q}, \hat{\gamma}]$

Initialization: Γ^0

while *! stopping condition for EM* **do**

▷ `At` $(k+1)^{th}$ `EM iteration with estimates` $\boldsymbol{\Gamma}^k = [p^k, q^k, \gamma^k, \alpha^k, \beta^k]$)

▷ `Sample` B `particles` $\{(g^{(b)}(t_m), w^{(b)}(t_m))\}_{b=1}^{B}$ `at each` t_m `(particle filtering)`

Set $g^{(b)}(t_1) = g^\star(t_1)$, $g^{(b)}(t_1) = 1$ for each b.

for $m = 1, 2, ..., M-1$ **do**

for $b = 1, ..., B$ **do**

Calculate the conditional probability $p_b^m := P(G^\star(t_m) = g^\star(t_m) | G(t_m) = g^{(b)}(t_m))$

end

Sample B index $\eta_1, \eta_2, ...\eta_B$ from set $(1, 2, ..., B)$ with replacement with probability proportional to $p_1^m, p_2^m, ..., p_B^m$.

for $b = 1, ..., B$ **do**

Start from $g^{(\eta_b)}(t_m), w^{(\eta_b)}(t_m)$, simulate according to ER/PR up until time $t_m - t_{m-1}$, then set $g^{(b)}(t_{m+1}), w^{(b)}(t_{m+1})$ to be the current simulated network and binary variable.

end

end

▷ `Sample ancestral lines and simulate sample paths`

for $h = 1, 2, .., H$ **do**

Sample an index η from $1, 2, ..., B$ with probability proportional to $p_1^M, p_2^M, ..., p_B^M$, trace back the ancestral line for the η-th particle at time t_M, then obtain one sequence of networks and binary variables, $\boldsymbol{g}_{2:M}^{(h)}, \boldsymbol{w}_{2:M}^{(h)}$

for $d = 1, 2, ..., D$ **do**

Sample one path $\boldsymbol{s}_h^{(d)}, \boldsymbol{v}_h^{(d)}$ from $\boldsymbol{S}, \boldsymbol{V} | \boldsymbol{W} = \boldsymbol{w}_{2:M}^{(h)}, \boldsymbol{G} = \boldsymbol{g}_{2:M}^{(h)}$ by Metropolis–Hastings.

Calculate and store the sufficient statistics.

end

end

▷ `Update parameters`

Update $\hat{p}^{k+1}, \hat{q}^{k+1}, \hat{\gamma}^{k+1}$ using formulas for $\hat{\gamma}, \hat{p}, \hat{q}$ in (9)-(11) in the main text with sampled paths $\{\boldsymbol{s}_h^{(d)}, \boldsymbol{v}_h^{(d)}\}_{d=1}^{D}$ that interpolate $\boldsymbol{w}_{2:M}^{(h)}, \boldsymbol{g}_{2:M}^{(h)}$, $h = 1, \cdots, H$.

Draw Ψ ancestral lines, then update $\hat{\alpha}^{k+1}, \hat{\beta}^{k+1}$ using formulas for $\hat{\alpha}$ in (6) in the main text with sampled network sequences $\{\boldsymbol{g}_{2:M}^{(\psi)}\}_{\psi=1}^{\Psi}$.

end

2 Details on Computing Marginal Probability of Observations

2.1 Derivation of Recurrence Relation in Equ (5) in the Main Text

Define $Z_m := P\Big(\boldsymbol{G}^\star_{2:m} = \boldsymbol{g}^\star_{2:m} | \boldsymbol{X}_1 = \big(1, g^\star(t_1)\big)\Big)$ for each $m = 2, \cdots M$, and $Z_1 := 1$. Also let $q_m(x) = P(\boldsymbol{G}^\star_m = \boldsymbol{g}^\star_m | \boldsymbol{X}_m = x)$, $\eta_m(x) = P(\boldsymbol{X}_m = x | \boldsymbol{G}^\star_{2:m-1} = \boldsymbol{g}^\star_{2:m-1}, \boldsymbol{X}_1 = (1, g^\star(t_1))$ for each $m = 2, \cdots M$, with conventions $\eta_2(x) = P(\boldsymbol{X}_2 = x | \boldsymbol{X}_1 = (1, g^\star(t_1))$. To alleviate the notational burden, we omit the condition at t_1 in the probability expression when no confusion is possible.

$$
\begin{aligned}
Z_m &= P(\boldsymbol{G}^\star_{2:m} = \boldsymbol{g}^\star_{2:m}) = P(\boldsymbol{G}^\star_{2:m-1} = \boldsymbol{g}^\star_{2:m-1}) P(\boldsymbol{G}^\star_m = \boldsymbol{g}^\star_m | \boldsymbol{G}^\star_{2:m-1} = \boldsymbol{g}^\star_{2:m-1}) \\
&= Z_{m-1} \sum_x P(\boldsymbol{G}^\star_m = \boldsymbol{g}^\star_m, \boldsymbol{X}_m = x | \boldsymbol{G}^\star_{2:m-1} = \boldsymbol{g}^\star_{2:m-1}) \\
&= Z_{m-1} \sum_x P(\boldsymbol{G}^\star_m = \boldsymbol{g}^\star_m | \boldsymbol{X}_m = x, \boldsymbol{G}^\star_{2:m-1} = \boldsymbol{g}^\star_{2:m-1}) P(\boldsymbol{X}_m = x | \boldsymbol{G}^\star_{2:m-1} = \boldsymbol{g}^\star_{2:m-1}) \\
&= Z_{m-1} \sum_x P(\boldsymbol{G}^\star_m = \boldsymbol{g}^\star_m | \boldsymbol{X}_m = x) P(\boldsymbol{X}_m = x | \boldsymbol{G}^\star_{2:m-1} = \boldsymbol{g}^\star_{2:m-1}) \\
&= Z_{m-1} \sum_x q_m(x) \eta_m(x) \\
&= Z_{m-1} E(q_m(\boldsymbol{X})), \boldsymbol{X} \sim \eta_m(\cdot)
\end{aligned}
\tag{2.1}
$$

2.2 A Forward Algorithm with Particle Filtering

We now present a forward algorithm with particle filtering to compute Z^B_M, i.e. the particle approximation of the marginal probability of observations. The time complexity is $\mathcal{O}(BM)$. Most of

Algorithm 2: Forward Algorithm with Particle Filtering

Input: $\boldsymbol{g}^\star = \big(g^\star(t_1), g^\star(t_2), ..., g^\star(t_M)\big)$, B
Output: Z^B_M

1 Set $Z^B_1 = 1$, sample $\xi^b_2 \overset{iid}{\sim} \eta_2(\cdot) = P\big(\boldsymbol{X}_2 = \cdot \,|\, \boldsymbol{X}_1 = (1, g(t_1))\big)$, $b = 1, \cdots, B$, and set $\eta^B_2(\cdot) = \frac{1}{B} \sum_{b=1}^B \mathbf{1}\{\xi^b_2 = \cdot\}$

2 For $m = 2, \cdots M$: set

$$Z^B_m \leftarrow Z^B_{m-1} \sum_x q_m(x) \eta^B_m(x) = Z^B_{m-1} \frac{1}{B} \sum_{b=1}^B q_m(\xi^b_m)$$

Sample

$$\xi^b_{m+1} \overset{iid}{\sim} \eta^B_{m+1}(\cdot) = \sum_{b=1}^B \frac{q_m(\xi^b_m) P(\boldsymbol{X}_{m+1} = \cdot \,|\, \boldsymbol{X}_m = \xi^b_m)}{\sum_{i=1}^B q_m(\xi^b_m)}, \, b = 1, \cdots, B \tag{$\star$}$$

and set $\eta^B_{m+1}(\cdot) = \sum_{b=1}^B \mathbf{1}\{\xi^b_{m+1} = \cdot\}$

the computation is in the simulation of ($\star$), which can be done as in step 3 of section 1.3.

The time complexity of this algorithm is $\mathcal{O}(BM)$. Thus the full complexity to compare the two percolation regimes is $\mathcal{O}\big((B + H + \Psi)M\big)$.

3 Details on Automatic Segment Finder

Before we apply our estimation and testing framework to the constructed networks, we need first choose a segment of the network time series consistent with percolation in connection with different stages of seizure, e.g. seizure onset and preceding termination. The choice of an appropriate time series segment from the overall non-stationary signal is nontrivial. We developed a simple, automatic procedure for the purpose, shown in Algorithm 3. This procedure consists of the following steps:

1. Subset the time series by windowing so that it only contains network evolution in the region of interest (ROI).
2. Split the time series into different low-high-low segments based on GCC/density of the network time series. Each low-high-low segment starts when the size of the GCC/density is below low level and ends when it reaches low level for the first time after reaching high level. The two levels are set to be the first and third quartiles of the size of the GCC/density over time.
3. For each low-high-low segment, trim off the tail with decreased GCC/density, which is from when it reaches high level for the last time to the end.
4. Use the identified low-high segments for testing.

The purpose of step 2-3 is to automatically identify all segments where both the size of GCC and density ramp up in the ROI. There are several ways proposed to choose the ROIs in a network time series. One way is to choose the segment from 50 s before seizure onset to 50 s after seizure onset. The second way is via the subjective determination of an expert. The third way is through dynamic community detection on network time series [4], and to choose the region where the large dynamic community appears. We used the third in our analysis since it is the most objective approach and yields reliable ROIs assessed by epileptologist.

Algorithm 3: Automatic Segment Finder

Input: Network time series $\boldsymbol{g}^\star_{1:M} = [g^*(t_1), \cdots, g^*(t_M)]$, ROI $= [t', t'']$, metric on networks: GCC or Density

Output: A network time series segment $\boldsymbol{g}^\star_{a:b} = [g^*(t_a), g^*(t_{a+1}) \cdots, g^*(t_b)], 1 \le a < b \le M$

Initialize: *set* $= \{\}$ an empty set to store segmenting indices
$\boldsymbol{c}_{1:M} \in \mathbb{R}^M \leftarrow \text{GCC}(\boldsymbol{g}^\star_{1:M})/\text{Density}(\boldsymbol{g}^\star_{1:M})$ ▷ `Calculate size of GCC/density over time`
$l, r \leftarrow \underset{l,r \in 1:M}{\arg\max}(t_r - t_l)$ s.t. $t_l, t_r \in$ ROI ▷ `Take a subsection of time series`
$Q_1 \leftarrow$ first quartile of $\boldsymbol{c}_{l:r}$
$Q_3 \leftarrow$ third quartile of $\boldsymbol{c}_{l:r}$
$anchor \leftarrow l$ ▷ `Start segmenting time series with sliding window`
while $\boldsymbol{c}_{anchor} > Q_1$ **do**
  $anchor \leftarrow anchor + 1$ ▷ `Anchor the left point of a potential segment`
end
$set = \{set, anchor\}$
while *not finished segmenting* $\boldsymbol{c}_{l:r}$ **do**
  $i \leftarrow 1$
  while $\boldsymbol{c}_{anchor+i} < Q_3$ & $anchor + i < r$ **do**
    $i \leftarrow i + 1$ ▷ `Grow the segment until it reaches` Q_3
  end
  while $\boldsymbol{c}_{anchor+i} > Q_1$ & $anchor + i < r$ **do**
    $i \leftarrow i + 1$ ▷ `Keep growing the segment until it reaches` Q_1
  end
  $anchor \leftarrow anchor + i$ ▷ `Anchor the right point of the grown segment`
  $set = \{set, anchor\}$
end
$a, b' \leftarrow$ a pair of consecutive indices in *set* s.t. $\tilde{t} \in [a, b']$
$b \leftarrow b'$
while $\boldsymbol{c}_b < Q_3$ **do**
  $b \leftarrow b - 1$ ▷ `Trim off the tail of segment`
end

Note that instead of using either GCC or density to segment the network time series, we can also use both of them to find the proper segment for testing. Specifically, we can first run the Automatic Segment Finder on the network time series with one metric. We can then run it again with the other metric, thus obtaining two segmented network time series. And then we can take, as input, the overlapping of the two segments for testing, which is what we do in this analysis.

4 Model Identifiability for RG-HMM

In this section, we address the identifiability of RG-HMM. The model is identifiable if distinct values of $\boldsymbol{\Gamma}$ correspond to distinct probability distributions, i.e. if $\boldsymbol{\Gamma}_1 \neq \boldsymbol{\Gamma}_2$, then also $P_{\boldsymbol{\Gamma}_1} \neq P_{\boldsymbol{\Gamma}_2}$. We show that $f(g^\star)$ is identifiable if type-I error $\alpha < 0.5$ and type-II error $\beta < 0.5$.

We will show that two sets of parameters $\boldsymbol{\Gamma}_1 = (p_1, q_1, \gamma_1, \alpha_1, \beta_1)$, and $\boldsymbol{\Gamma}_2 = (p_2, q_2, \gamma_2, \alpha_2, \beta_2)$ where $p_2 = q_1, q_2 = p_1, \alpha_2 = 1 - \beta_1, \beta_2 = 1 - \alpha_1$ will give the same probability distribution of the observed networks, written as $f\big(g^\star(t_1), \cdots, g^\star(t_M)\big)$. We will then assert that this is the only parameterization that will cause non-identifiability, thus we can make the model identifiable by

restricting the parameter space: $\alpha < 0.5, \beta < 0.5$.

We now show that $f_{\mathbf{\Gamma}_1}\big(g^\star(t_1), \cdots, g^\star(t_M)\big) = f_{\mathbf{\Gamma}_2}\big(g^\star(t_1), \cdots, g^\star(t_M)\big), \forall g^\star(t_1), \cdots, g^\star(t_M)$. Let $\boldsymbol{x}(t_m) = (g(t_m), w(t_m))$ denote the latent variables in the hidden layer, then conditional on $\boldsymbol{x}(t_1) = (g^\star(t_1), 1)$,

$$\begin{aligned}
& f_{\mathbf{\Gamma}_1}\big(g^\star(t_1), \cdots, g^\star(t_M)\big) \\
= & \sum_{\boldsymbol{x}(t_1),\cdots,\boldsymbol{x}(t_M)} f_{\mathbf{\Gamma}_1}\big(g^\star(t_1), \cdots, g^\star(t_M), \boldsymbol{x}(t_1), \cdots, \boldsymbol{x}(t_M)\big) \\
= & \sum_{\boldsymbol{x}(t_1),\cdots,\boldsymbol{x}(t_M)} f\big(\boldsymbol{x}(t_1)\big) f_{\alpha_1,\beta_1}\big(g^\star(t_1)|g(t_1)\big) \prod_{m=2}^{M} f_{\alpha_1,\beta_1}\big(g^\star(t_m)\big|g(t_m)\big) f_{p_1,q_1,\gamma_1}\big(\boldsymbol{x}(t_m)\big|\boldsymbol{x}(t_{m-1})\big) \\
= & \sum_{\boldsymbol{x}(t_2),\cdots,\boldsymbol{x}(t_M)} \prod_{m=2}^{M} f_{\alpha_1,\beta_1}\big(g^\star(t_m)\big|g(t_m)\big) f_{p_1,q_1,\gamma_1}\big(\boldsymbol{x}(t_m)\big|\boldsymbol{x}(t_{m-1})\big).
\end{aligned} \tag{4.1}$$

For any potential true network $g(t_m)$, there exists an 'opposite' network $g'(t_m)$ obtained by flipping all edges of $g(t_m)$ to non-edges and all non-edges to edges. Hence, if given the observed network $g^\star(t_m)$, $g(t_m)$ contains a true edges, b false non-edges, c false edges and d true non-edges, then given $g^\star(t_m)$, $g'(t_m)$ will contain c true edges, d false non-edges, a false edges and b true non-edges. Note that

$$\begin{aligned}
f_{\alpha_1,\beta_1}\big(g^\star(t_m)|g(t_m)\big) &= \alpha_1^c(1-\alpha_1)^d\beta_1^b(1-\beta_1)^a \\
f_{\alpha_2,\beta_2}\big(g^\star(t_m)|g'(t_m)\big) &= \alpha_2^a(1-\alpha_2)^b\beta_2^d(1-\beta_2)^c,
\end{aligned} \tag{4.2}$$

then we have $f_{\alpha_1,\beta_1}\big(g^\star(t_m)|g(t_m)\big) = f_{\alpha_2,\beta_2}\big(g^\star(t_m)|g'(t_m)\big)$ if $\alpha_2 = 1-\beta_1, \beta_2 = 1-\alpha_1$.

Define $\boldsymbol{x}'(t_m) = \big(g'(t_m), w'(t_m)\big)$, where $w'(t_m) = 1 - w(t_m)$. Next we show that $f_{p_1,q_1,\gamma_1}\big(\boldsymbol{x}(t_m)\big|\boldsymbol{x}(t_{m-1})\big) = f_{p_2,q_2,\gamma_2}\big(\boldsymbol{x}'(t_m)\big|\boldsymbol{x}'(t_{m-1})\big)$ if $p_1 = q_2$, $p_2 = q_1$, $\gamma_2 = \gamma_1$ for ER process. Then we have

$$\begin{aligned}
f_{p_1,q_1,\gamma_1}\big(\boldsymbol{x}(t_m)\big|\boldsymbol{x}(t_{m-1})\big) = \sum_{\boldsymbol{u}} \Big[& e^{-\gamma_1(t_m-t_{m-1})}\frac{(\gamma_1(t_m-t_{m-1})^R}{R!}\cdot \\
& \prod_{r=1}^{R} f\big(g(\tau_r)\big|w(\tau_r), g(\tau_{r-1})\big) f\big(w(\tau_r)\big|g(\tau_{r-1}), w(\tau_{r-1})\big)\Big],
\end{aligned} \tag{4.3}$$

where the sum is over all possible sample paths connecting $\boldsymbol{x}(t_{m-1})$ and $\boldsymbol{x}(t_m)$. Details for the pmf of a sample path are discussed in Appendix A. Let $\boldsymbol{u}'$ be the 'opposite' sample path of $\boldsymbol{u}$, which can be obtained by flipping all $(g(\tau_r), w(\tau_r))$ in $\boldsymbol{u}$ into $(g'(\tau_r), 1-w(\tau_r))$. Hence if $\boldsymbol{u}$ is a sample path connecting $\boldsymbol{x}(t_{m-1})$ and $\boldsymbol{x}(t_m)$, $\boldsymbol{u}'$ will then be a sample path connecting $\boldsymbol{x}'(t_{m-1})$ and $\boldsymbol{x}'(t_m)$. If we fix the rate parameter $\gamma_1 = \gamma_2$, then it is sufficient to show that $f\big(g(\tau_r)\big|w(\tau_r), g(\tau_{r-1})\big) f\big(w(\tau_r)\big|g(\tau_{r-1}), w(\tau_{r-1})\big) = f\big(g'(\tau_r)\big|w\prime(\tau_r), g\prime(\tau_{r-1})\big) f\big(w\prime(\tau_r)\big|g\prime(\tau_{r-1}), w\prime(\tau_{r-1})\big)$ for each τ_r. Assume that $g(\tau_{r-1})$ contains a edges and b non-edges, then $g'(\tau_{r-1})$ will contain b edges and a non-edges. Note that under ER process,

$$f\big(g(\tau_r)\big|w(\tau_r), g(\tau_{r-1})\big) f\big(w(\tau_r)\big|g(\tau_{r-1}), w(\tau_{r-1})\big) = \begin{cases} \frac{p_1}{b}, \text{ if } w(\tau_r) = 1, w(\tau_{r-1}) = 0 \\ \frac{1-q_1}{b}, \text{ if } w(\tau_r) = 1, w(\tau_{r-1}) = 1 \\ \frac{1-p_1}{a}, \text{ if } w(\tau_r) = 0, w(\tau_{r-1}) = 0 \\ \frac{q_1}{a}, \text{ if } w(\tau_r) = 0, w(\tau_{r-1}) = 1 \end{cases} \tag{4.4}$$

$$f\big(g'(\tau_r)\big|w\prime(\tau_r), g\prime(\tau_{r-1})\big) f\big(w\prime(\tau_r)\big|g\prime(\tau_{r-1}), w\prime(\tau_{r-1}\big) = \begin{cases} \frac{p_2}{a}, \text{ if } w\prime(\tau_r) = 1, w\prime(\tau_{r-1}) = 0 \\ \frac{1-q_2}{a}, \text{ if } w\prime(\tau_r) = 1, w\prime(\tau_{r-1}) = 1 \\ \frac{1-p_2}{b}, \text{ if } w\prime(\tau_r) = 0, w\prime(\tau_{r-1}) = 0 \\ \frac{q_2}{b}, \text{ if } w\prime(\tau_r) = 0, w\prime(\tau_{r-1}) = 1, \end{cases} \tag{4.5}$$

then (4.4) and (4.5) are equivalent if $p_2 = p_1$ and $p_1 = p_2$. For PR process, it's not necessarily true since $f\big(g(\tau_r)\big|w(\tau_r), g(\tau_{r-1})\big)$ depends on the sizes of connected components of $g(\tau_r)$ and the relation between the connected components of $g(\tau_r)$ and that of $g'(\tau_r)$ is intractable and hard to write into analytical forms.

Since each summand in (4.1) under paramerterization $\mathbf{\Gamma}_1 = (p_1, q_1, \gamma_1, \alpha_1, \beta_1)$ will have one and only one match to a summand under the paramerterization $\mathbf{\Gamma}_2 = (p_2 = q_1, q_2 = p_1, \gamma_1 = \gamma_2, \alpha_1 = 1 - \beta_2, \beta_1 = 1 - \alpha_2)$ by flipping the latent variables $\{\boldsymbol{x}(t_m)\}$ into $\{\boldsymbol{x}'(t_m)\}$, then $f_{\mathbf{\Gamma}_1}\big(g^\star(t_1), \cdots, g^\star(t_M)\big) = f_{\mathbf{\Gamma}_2}\big(g^\star(t_1), \cdots, g^\star(t_M)\big)$. Therefore, the probability distributions of observed networks are the same under the two parameterization explained above, causing non-identifiability. By restricting the parameter space $\alpha < 0.5, \beta < 0.5$, we can make the model identifiable.

5 Asymptotic Properties of MLE for RG-HMM

5.1 Asymptotic normality

We provide a convergence result for the MLE in our proposed RG-HMM in its stationary period as time progresses. Let $\mathbf{\Gamma}_0$ and $\hat{\mathbf{\Gamma}}$ denote the true values of parameters and the MLE of RG-HMM, respectively. To simplify the notation, let $\boldsymbol{X}(t_m)$ denote the pair $(W(t_m), G(t_m))$.

The asymptotic behavior of $\hat{\mathbf{\Gamma}}$ in our RG-HMM can be established using results from [5] on asymptotic normality of the MLE for a general stationary HMM.

Theorem 5.1. *Let $\pi_{\mathbf{\Gamma}}(\boldsymbol{x})$ be the stationary distribution of a Markov chain in RG-HMM $\{\boldsymbol{X}(t_m), G^\star(t_m)\}$ and $\mathcal{L}_0$ be the limiting covariance matrix of $m^{-1/2}\frac{\partial}{\partial \mathbf{\Gamma}} \log p_{\mathbf{\Gamma}}(\boldsymbol{G}^\star_{1:m})$. Assume (C1) that $t_m - t_{m-1} = t_{m+1} - t_m = \Delta t, \forall\, m \geq 2$, and stationarity is reached at t_1, (C2) that $\forall\, \boldsymbol{x} \in \mathcal{X}$, $\mathbf{\Gamma} \to \pi_{\mathbf{\Gamma}}(\boldsymbol{x})$ has two continuous derivatives in some small neighborhood of $\mathbf{\Gamma}_0$, and (C3) that $\mathcal{L}_0$ is nonsingular. Then $m^{1/2}(\hat{\mathbf{\Gamma}} - \mathbf{\Gamma_0}) \xrightarrow{p} N(0, \mathcal{L}_0^{-1})$ as $m \to \infty$.*

The proof of Theorem 5.1 is given in the following subsection. We establish the asymptotic property of the MLE for our RG-HMMs in its stationary period using asymptotic results for general HMMs with stationary Markov chains [5]. Unfortunately, no proof is yet available for our model in its nonstationary period when networks evolve through the percolation phase transition. The most relevant work on asymptotic theory for HMMs with nonstationary Markov chains is in [6]. This is an interesting subject for future research.

5.2 Proof of Theorem 5.1

Proof. [5] provide a central limit theorem for the MLE of a general HMM with stationary Markov chain under 6 regularity conditions (A1)-(A6). Our approach is to show that (A1)-A(6) hold for our RG-HMM under assumptions (C1)-(C3).

Let $P_{ab}(t)$ denote the probability that the Markov chain $\{\boldsymbol{X}(t), t \geq 0\}$ in our RG-HMM presently in state a will be in state b after an additional time t, P_{ab} denote the one-step transition probability

that the process will next enter state b when it leaves state a (details in Appendix B), P_{ab}^n denote the n-step transition probability.

Under (C1), the M observation moments $t_1 < t_2 < \cdots < t_M$ are evenly spaced at the stationary period of Markov chain, then the transition probability for the discrete-time Markov chain $\{\boldsymbol{X}(t_m), m \geq 1\}$ in our RG-HMM is $P(\boldsymbol{X}(t_m) = b|\boldsymbol{X}(t_{m-1}) = a) = P_{ab}(t_m - t_{m-1}) = P_{ab}(\Delta t), \forall\, m$, which is independent of m. Hence, (C1) ensures that $\{\boldsymbol{X}(t_m)\}$ is a stationary Markov chain with homogeneous transition probability matrix $\{\alpha_{\boldsymbol{\Gamma}_0}(a,b)\} = \{P_{ab}(\Delta t)\}$. We now restate (A1)-(A6) and show that they all hold for our RG-HMM $\{\boldsymbol{X}(t_m), G^\star(t_m)\}$.

(A1) The transition probability matrix $\{\alpha_{\boldsymbol{\Gamma}_0}(a,b)\}$ is ergodic, that is, irreducible and aperiodic.

(A2) For all a, $b \in \mathcal{X}$ and $g^\star \in \{1, 2, ..., 2^{\binom{N}{2}}\}$, the maps $\boldsymbol{\Gamma} \to \alpha_{\boldsymbol{\Gamma}}(a,b)$, $\boldsymbol{\Gamma} \to \pi_{\boldsymbol{\Gamma}}(a)$ and $\boldsymbol{\Gamma} \to g_{\boldsymbol{\Gamma}}(g^\star|a)$ have two continuous derivatives in some neighborhood of $\boldsymbol{\Gamma}_0$.

(A3) Write $\boldsymbol{\Gamma} = (\Gamma_1, \cdots, \Gamma_d) \equiv (p, q, \gamma, \alpha, \beta)$. There exists a $\delta > 0$ such that (i) for all $1 \leq i \leq d$ and all a,

$$E_{\boldsymbol{\Gamma}_0}\Big[\sup_{|\boldsymbol{\Gamma}-\boldsymbol{\Gamma}_0|<\delta} |\frac{\partial}{\partial \Gamma_i} \log g_{\boldsymbol{\Gamma}}(G^\star|a)|^2\Big] < \infty; \tag{5.1}$$

(ii) for all $a \leq i, j \leq d$ and all a,

$$E_{\boldsymbol{\Gamma}_0}\Big[\sup_{|\boldsymbol{\Gamma}-\boldsymbol{\Gamma}_0|<\delta} |\frac{\partial^2}{\partial \Gamma_i \partial \Gamma_j} \log g_{\boldsymbol{\Gamma}}(G^\star|a)|\Big] < \infty; \tag{5.2}$$

(iii) for $j = 1, 2$, all $1 \leq i_l \leq d$, $l = 1, \cdots, j$ and all a,

$$\int \sup_{|\boldsymbol{\Gamma}-\boldsymbol{\Gamma}_0|<\delta} |\frac{\partial^j}{\partial \Gamma_{i_1} \partial \Gamma_{i_j}} g_{\boldsymbol{\Gamma}}(g^\star|a)|\nu(dg^\star) < \infty. \tag{5.3}$$

(A4) There exists a $\delta > 0$ such that with

$$\rho(g^\star) = \sup_{|\boldsymbol{\Gamma}-\boldsymbol{\Gamma}_0|<\delta} \max_{a,b\in\mathcal{X}} \frac{g_{\boldsymbol{\Gamma}}(g^\star|a)}{g_{\boldsymbol{\Gamma}}(g^\star|b)}, \tag{5.4}$$

$P_{\boldsymbol{\Gamma}_0}(\rho(G^\star(t_m)) = \infty|\boldsymbol{X}(t_m) = a) < 1$ for all a.

(A5) $\boldsymbol{\Gamma}_0$ is an interior point of the set Θ, which is the set to which $\boldsymbol{\Gamma}$ belong.

(A6) The MLE is strongly consistent, which requires (i) (A1); (ii) for all a, b and $g^\star$, the map $\boldsymbol{\Gamma} \to \alpha_{\boldsymbol{\Gamma}}(a,b)$ and the map $\boldsymbol{\Gamma} \to g_{\boldsymbol{\Gamma}}(g^\star|a)$ are continuous on Θ; (iii) for each a, $E_{\boldsymbol{\Gamma}_0}|\log g_{\boldsymbol{\Gamma}_0}(G^\star|a)| < \infty$; (iv) for each a and $\boldsymbol{\Gamma}$ there is a $\delta > 0$ such that $E_{\boldsymbol{\Gamma}_0}\Big[\sup_{|\boldsymbol{\Gamma}'-\boldsymbol{\Gamma}|<\delta}(\log g_{\boldsymbol{\Gamma}'}(G^\star|a))^+\Big] < \infty$; (v) for each $\boldsymbol{\Gamma}$ such that the laws $P_{\boldsymbol{\Gamma}}$ and $P_{\boldsymbol{\Gamma}_0}$ agree, $\boldsymbol{\Gamma} = \boldsymbol{\Gamma}_0$.

In our RG-HMM, all states in the Markov chain $\{\boldsymbol{X}(t_m)\}$ communicate with each other as any two networks can be connected by a network path that leads the one to the other, hence irreducible. Note that $|\mathcal{X}| < \infty$, then $\{\boldsymbol{X}(t_m)\}$ is positive recurrent as every irreducible Markov chain with a finite state space is positive recurrent. Also note that the transition probability

$$\alpha_{\boldsymbol{\Gamma}_0}(a,b) = P_{ab}(\Delta t) = \sum_{n=0}^{\infty} P_{ab}^n \cdot \exp(-\gamma \Delta t) \frac{(\gamma \Delta t)^n}{n!} \in (0, +\infty), \tag{5.5}$$

for all $a, b \in \mathcal{X}$, $p, q \in (0,1)$ and $\gamma \in (0, +\infty)$. Let $\alpha_{\boldsymbol{\Gamma}_0}^{(k)}(a,b)$ denote the k-step transition probability of $\{\boldsymbol{X}(t_m)\}$, then the matrix $\{\alpha_{\boldsymbol{\Gamma}_0}^{(k)}(a,b)\}$ can be calculated by multiplying the matrix $\{\alpha_{\boldsymbol{\Gamma}_0}(a,b)\}$ by itself k times, yielding positive diagonal elements, which means that each state can visit back

itself in any number of transition steps, hence it is aperiodic. Therefore, (A1) holds, which also assures the existence of unique stationary distribution for $\{\boldsymbol{X}(t_m)\}$.

Note that P_{ab} is given in Appendix B (example with 3-node network is provided in the end of the proof), and the matrix $\{P_{ab}^n\}$ can be calculated by multiplying the matrix $\{P_{ab}\}$ by itself n times, thus we have

$$P_{ab}^n = \sum_{i=1}^{k(n)} c_{0i} p^{c_{1i}} q^{c_{2i}} (1-p)^{c_{3i}} (1-q)^{c_{4i}}, \tag{5.6}$$

where $\{c_{ji}\}_{j=0}^4$ and $k(n)$ are constant depending on the values of a, b and n. c_{0i} is the term corresponding to the product of probabilities of random graph process, thus $c_{0i} \leq 1$. $k(n)$ can be interpreted as the number of feasible paths from a to b with length n, and each path contains $c_{1i} + c_{4i}$ edge additions and $c_{2i} + c_{3i}$ edge deletions, thus $0 \leq c_{1i}, c_{2i}, c_{3i}, c_{4i} \leq n$. Also we have

$$g_{\boldsymbol{\Gamma}}(g^\star|a) = \alpha^c (1-\alpha)^d \beta^b (1-\beta)^a, \tag{5.7}$$

where a, b, c, d are constant depending on the true network a and observed network $g^\star$.

From the analytical form of $\alpha_{\boldsymbol{\Gamma}_0}(a,b)$ and $g_{\boldsymbol{\Gamma}}(g^\star|a)$ given in (5.5), (5.6) and (5.7), along with assumption (C2), identifiability addressed in Section 4 and finite state space $\mathcal{X}$, we can verify that when $p, q \in (0,1)$, $\gamma > 0$ and $\alpha, \beta \in (0, 0.5)$, (A2)-(A6) all hold. (A3) holds since the derivatives of $g_{\boldsymbol{\Gamma}}(g^\star|a)$ w.r.t α and β are bounded in some neighborhood of Γ_0, and the expectation and integral involve finite summation of finite terms, thus finite as well. (A4) holds since $g_{\boldsymbol{\Gamma}}(g^\star|a) \neq 0$ with $\alpha, \beta \in (0, 0.5)$, and $\rho(g^\star) < \infty$ for all $g\star$ and a. (A5) holds since Θ is an open set, $\Theta = \{(p, q, \gamma, \alpha, \beta) \,|\, p, q \in (0,1), \gamma \in (0, \infty), \alpha, \beta \in (0, 0.5)\}$. (A6)-(i)(iii)(iv)(v) all hold since $g_{\boldsymbol{\Gamma}}(g^\star|a)$ are bounded in some neighborhood of Γ_0, and the model is identifiable under set Θ.

As it is hard to verify analytically the continuity regarding $\boldsymbol{\Gamma} \to \pi_{\boldsymbol{\Gamma}}(a)$, we make it the assumption (C2). We can verify that (A2) and (A6)-(ii) also hold under (C2). To see this is so, we show, as an example, that $\alpha_{\boldsymbol{\Gamma}_0}(a,b)$ is continuous in $p \in (0,1)$ and the first and second derivatives of $\alpha_{\boldsymbol{\Gamma}_0}(a,b)$ w.r.t p are continuous in some neighborhood of p_0. The continuity w.r.t other parameters q, γ, α, β can be verified using a similar argument. Define

$$f_n(p) := P_{ab}^n \cdot \exp(-\gamma\Delta t) \frac{(\gamma\Delta t)^n}{n!} = \exp(-\gamma\Delta t) \frac{(\gamma\Delta t)^n}{n!} \cdot \sum_{i=1}^{k(n)} c_{0i} p^{c_{1i}} q^{c_{2i}} (1-p)^{c_{3i}} (1-q)^{c_{4i}}, \tag{5.8}$$

$$f(p) := \alpha_{\boldsymbol{\Gamma}_0}(a,b) = \sum_{n=0}^{\infty} f_n(p). \tag{5.9}$$

Note that for any γ, p and n, $|f_n(p)| \leq M_n := \exp(-\gamma\Delta t)\frac{(\gamma\Delta t)^n}{n!}$, and $\sum_{n=1}^{\infty} M_n = 1 < \infty$, then by Weierstrass M-test $\sum_{n=1}^{\infty} f_n(p)$ converges uniformly to $f(p)$ in $p \in (0,1)$. Note that $f_n(p)$ is continuous, then $f(p)$ is continuous in $(0,1)$. Under uniform convergence, we can interchange the order of summation and differentiation, which gives

$$f'(p) = \sum_{n=0}^{\infty} f_n'(p) = \sum_{n=0}^{\infty} \exp(-\gamma\Delta t) \frac{(\gamma\Delta t)^n}{n!} \cdot \sum_{i=1}^{k(n)} c_{0i} p^{c_{1i}} q^{c_{2i}} (1-p)^{c_{3i}} (1-q)^{c_{4i}} (\frac{c_{1i}}{p} - \frac{c_{3i}}{1-p}). \tag{5.10}$$

For $p \in (p_0 - \epsilon, p_0 + \epsilon)$, we have

$$\begin{aligned}\sum_{n=0}^{\infty} |f_n'(p)| &\leq \sum_{n=0}^{\infty} \exp(-\gamma\Delta t)\frac{(\gamma\Delta t)^n}{n!} \cdot \sum_{i=1}^{k(n)} c_{0i} p^{c_{1i}} q^{c_{2i}} (1-p)^{c_{3i}} (1-q)^{c_{4i}} (\left|\frac{c_{1i}}{p}\right| + \left|\frac{c_{3i}}{1-p}\right|) \\ &\leq \sum_{n=0}^{\infty} \exp(-\gamma\Delta t)\frac{(\gamma\Delta t)^n}{n!} \cdot \sum_{i=1}^{k(n)} c_{0i} p^{c_{1i}} q^{c_{2i}} (1-p)^{c_{3i}} (1-q)^{c_{4i}} (\left|\frac{n}{p}\right| + \left|\frac{n}{1-p}\right|) \\ &\leq \sum_{n=0}^{\infty} \exp(-\gamma\Delta t)\frac{(\gamma\Delta t)^n}{n!} \cdot (|\frac{n}{p}| + |\frac{n}{1-p}|) P_{ab}^n \\ &\leq (\frac{1}{|p|} + \frac{1}{|1-p|}) \sum_{n=0}^{\infty} \exp(-\gamma\Delta t)\frac{(\gamma\Delta t)^n}{n!} \cdot n \cdot 1 < \infty,\end{aligned} \tag{5.11}$$

so again by Weierstrass M-test $\sum_{n=1}^{\infty} f_n'(p)$ converges uniformly to $f'(p)$ in some neighborhood of p_0. Then we have for $p \in (p_0 - \epsilon, p_0 + \epsilon)$

$$\begin{aligned}f''(p) = \sum_{n=0}^{\infty} f_n''(p) = \sum_{n=0}^{\infty} \exp(-\gamma\Delta t)\frac{(\gamma\Delta t)^n}{n!} \cdot \sum_{i=1}^{k(n)} c_{0i} p^{c_{1i}} q^{c_{2i}} (1-p)^{c_{3i}} (1-q)^{c_{4i}} \Bigg[(\frac{c_{1i}}{p} - \frac{c_{3i}}{1-p})^2 \\ - \frac{c_{1i}}{p^2} - \frac{c_{3i}}{(1-p)^2} \Bigg],\end{aligned} \tag{5.12}$$

$$\begin{aligned}\sum_{n=0}^{\infty} |f_n''(p)| &\leq \sum_{n=0}^{\infty} \exp(-\gamma\Delta t)\frac{(\gamma\Delta t)^n}{n!} \cdot \sum_{i=1}^{k(n)} c_{0i} p^{c_{1i}} q^{c_{2i}} (1-p)^{c_{3i}} (1-q)^{c_{4i}} |(\frac{c_{1i}}{p} - \frac{c_{3i}}{1-p})^2 \\ &\qquad - \frac{c_{1i}}{p^2} - \frac{c_{3i}}{(1-p)^2} | \\ &\leq (\frac{1}{p^2} + \frac{1}{(1-p)^2} + \frac{2}{p(1-p)}) \sum_{n=0}^{\infty} \exp(-\gamma\Delta t)\frac{(\gamma\Delta t)^n}{n!} \cdot n^2 + \\ &\qquad (\frac{1}{p^2} + \frac{1}{(1-p)^2}) \sum_{n=0}^{\infty} \exp(-\gamma\Delta t)\frac{(\gamma\Delta t)^n}{n!} \cdot n < \infty,\end{aligned} \tag{5.13}$$

hence $\sum_{n=1}^{\infty} f_n''(p)$ converges uniformly to $f''(p)$ in some neighborhood of p_0. Since $f_n'(p)$ and $f_n''(p)$ are continuous in some neighborhood of p_0, and they are all uniformly convergent, then $f'(p)$ and $f''(p)$ are continuous in some neighborhood of p_0.

■

Example. The one-step transition matrix $\{P_{ab}\}$ for networks with 3 nodes under ER model is shown in (5.14), where the state space is $\mathcal{X} = \{0, 1\} \times \{1, 2, \cdots, 8\}) \setminus \{(0, 8), (1, 1)\}$ as complete network cannot be obtained by deleting an edge and empty network cannot be obtained by adding an edge. And the mapping between labels and networks is given in Figure 5.1.

$$
\{P_{ab}\} = \begin{array}{c|cccccccccccccc}
 & (0,1) & (0,2) & (0,3) & (0,4) & (0,5) & (0,6) & (0,7) & (1,2) & (1,3) & (1,4) & (1,5) & (1,6) & (1,7) & (1,8) \\
(0,1) & 0 & 0 & 0 & 0 & 0 & 0 & 0 & \frac{1}{3} & \frac{1}{3} & \frac{1}{3} & 0 & 0 & 0 & 0 \\
\hline
(0,2) & 1-p & 0 & 0 & 0 & 0 & 0 & 0 & 0 & 0 & 0 & \frac{1}{2}p & 0 & \frac{1}{2}p & 0 \\
(0,3) & 1-p & 0 & 0 & 0 & 0 & 0 & 0 & 0 & 0 & 0 & \frac{1}{2}p & \frac{1}{2}p & 0 & 0 \\
(0,4) & 1-p & 0 & 0 & 0 & 0 & 0 & 0 & 0 & 0 & 0 & 0 & \frac{1}{2}p & \frac{1}{2}p & 0 \\
\hline
(0,5) & 0 & \frac{1}{2}(1-p) & \frac{1}{2}(1-p) & 0 & 0 & 0 & 0 & 0 & 0 & 0 & 0 & 0 & 0 & p \\
(0,6) & 0 & 0 & \frac{1}{2}(1-p) & \frac{1}{2}(1-p) & 0 & 0 & 0 & 0 & 0 & 0 & 0 & 0 & 0 & p \\
(0,7) & 0 & \frac{1}{2}(1-p) & 0 & \frac{1}{2}(1-p) & 0 & 0 & 0 & 0 & 0 & 0 & 0 & 0 & 0 & p \\
\hline
(1,2) & q & 0 & 0 & 0 & 0 & 0 & 0 & 0 & 0 & 0 & \frac{1}{2}(1-q) & 0 & \frac{1}{2}(1-q) & 0 \\
(1,3) & q & 0 & 0 & 0 & 0 & 0 & 0 & 0 & 0 & 0 & \frac{1}{2}(1-q) & \frac{1}{2}(1-q) & 0 & 0 \\
(1,4) & q & 0 & 0 & 0 & 0 & 0 & 0 & 0 & 0 & 0 & 0 & \frac{1}{2}(1-q) & \frac{1}{2}(1-q) & 0 \\
\hline
(1,5) & 0 & \frac{1}{2}q & \frac{1}{2}q & 0 & 0 & 0 & 0 & 0 & 0 & 0 & 0 & 0 & 0 & 1-q \\
(1,6) & 0 & 0 & \frac{1}{2}q & \frac{1}{2}q & 0 & 0 & 0 & 0 & 0 & 0 & 0 & 0 & 0 & 1-q \\
(1,7) & 0 & \frac{1}{2}q & 0 & \frac{1}{2}q & 0 & 0 & 0 & 0 & 0 & 0 & 0 & 0 & 0 & 1-q \\
\hline
(1,8) & 0 & 0 & 0 & 0 & \frac{1}{3} & \frac{1}{3} & \frac{1}{3} & 0 & 0 & 0 & 0 & 0 & 0 & 0
\end{array} \tag{5.14}
$$

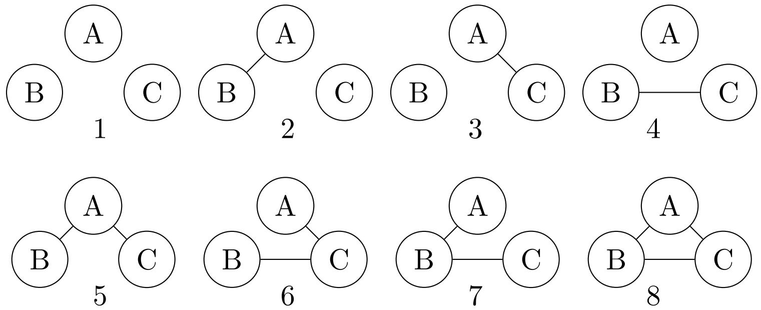

Figure 5.1: All possible network values for $G(t)$ with $N = 3$ nodes indexed from 1 to 8 in state space.

6 Supplementary Simulation Results

This section provides numerical results for Figure 6 and Figure 7 in the main text, and the testing results for the second simulation study designed to evaluate the effect of observation rate κ on the rate of detection for ER and PR using Bayes factor.

6.1 Numerical Simulation Results for Estimation

B	$\hat{p}$	$\hat{q}$	$\hat{\gamma}$	$\hat{\alpha}$	$\hat{\beta}$
50,000	0.675 (0.029)	0.289 (0.057)	1.739 (0.111)	0.112 (0.019)	0.037 (0.011)
250,000	0.681 (0.033)	0.278 (0.058)	1.768 (0.107)	0.092 (0.015)	0.030 (0.007)
500,000	0.698 (0.041)	0.286 (0.057)	1.779 (0.100)	0.083 (0.013)	0.029 (0.010)
1,000,000	0.698 (0.042)	0.291 (0.054)	1.794 (0.140)	0.079 (0.015)	0.026 (0.007)

Table 6.1: Numerical results for Figure 6 in the paper. Mean and standard deviation of parameter estimates based on 20 simulations from the ER process with varying B, $N = 20$, $M = 50$.

N	M	$\hat{p}$	$\hat{q}$	$\hat{\gamma}$	$\hat{\alpha}$	$\hat{\beta}$
30	50	0.637 (0.042)	0.335 (0.053)	1.647 (0.111)	0.089 (0.013)	0.034 (0.011)
	100	0.669 (0.029)	0.310 (0.044)	1.728 (0.066)	0.121 (0.017)	0.032 (0.008)
	150	0.684 (0.027)	0.307 (0.046)	1.802 (0.084)	0.143 (0.015)	0.029 (0.007)
	200	0.684 (0.024)	0.311 (0.034)	1.920 (0.096)	0.163 (0.023)	0.026 (0.005)
50	50	0.572 (0.038)	0.431 (0.078)	1.518 (0.046)	0.063 (0.005)	0.029 (0.008)
	100	0.613 (0.029)	0.374 (0.053)	1.581 (0.054)	0.082 (0.007)	0.034 (0.007)
	150	0.644 (0.026)	0.371 (0.027)	1.656 (0.062)	0.096 (0.009)	0.034 (0.006)
	200	0.653 (0.023)	0.337 (0.026)	1.691 (0.046)	0.107 (0.010)	0.038 (0.005)
70	50	0.526 (0.035)	0.537 (0.061)	1.526 (0.055)	0.050 (0.003)	0.024 (0.004)
	100	0.572 (0.030)	0.481 (0.060)	1.533 (0.051)	0.064 (0.004)	0.030 (0.007)
	150	0.602 (0.020)	0.444 (0.047)	1.535 (0.038)	0.075 (0.004)	0.033 (0.005)
	200	0.611 (0.025)	0.395 (0.037)	1.580 (0.037)	0.083 (0.005)	0.036 (0.006)

Table 6.2: Numerical results for Figure 7 in the paper. Mean and standard deviation of parameter estimates based on 20 simulations from ER process with $B = 50000$, $(N, M) \in \{30, 50, 70\} \times \{50, 100, 150, 200\}$.

6.2 Testing Results

A second simulation study is designed to evaluate the effect of observation rate κ on the rate of detection for ER and PR using Bayes factor. We set $B = 500,000$, while keeping the rate of change fixed at 2 ($\gamma = 2$). We let κ take on values in $\{0.5, 1, 1.5\}$ and N take on values in $\{10, 20, 30\}$. For each combination of settings, we generate 100 ER sequences and 100 PR sequences. We then conduct hypothesis testing on each sequence. We also fit a GLMM to analyze the testing results. The simulation and GLMM results are shown in Figures 6.1, 6.2 and Table 6.3. We can see that the observation rate significantly affect the rate of detection. The rate of detection significantly increases by increasing κ from 0.5 to 1, but stops to significantly increase as κ further increases.

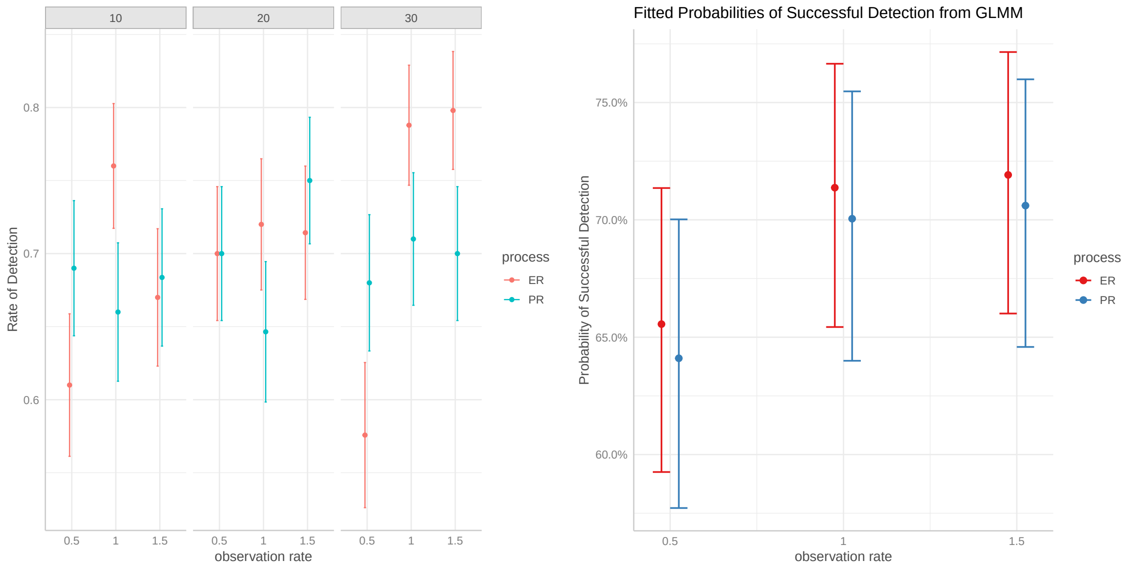

Figure 6.1: Results for the second simulation study. Left: rates of detection for ER and PR based on 100 replicates. Right: fitted probability of successful detection in GLMM.

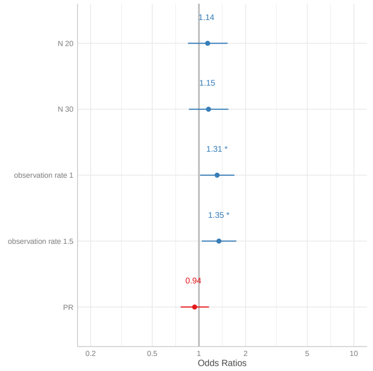

Figure 6.2: Estimates of the coefficients from GLMM in the second simulation study.

	Chisq	Df	Pr(>Chisq)
N	1.14	2	0.5668
process	0.36	1	0.5458
observation_rate	6.61	2	0.0368

Table 6.3: ANOVA table for the second simulation study

7 Supplementary Application Results

This section provides a full set of results for seizure 1, 2 and 3 on the left Hemisphere. For each seizure,we report the mean and standard deviation of parameter estimates under the two percolation models, the likelihood evaluated at those estimates, and the estimated Bayes factors in log scale. The results for all chosen segments (bold) in all three seizure are given in Tables in this section. Figure 7.1, Table 7.1, 7.2, 7.3 are for seizure 1; Figure 7.2, Table 7.4, 7.5, 7.6 are for seizure 2; Figure 7.3, Table 7.7, 7.8, 7.9, 7.10 are for seizure 3.

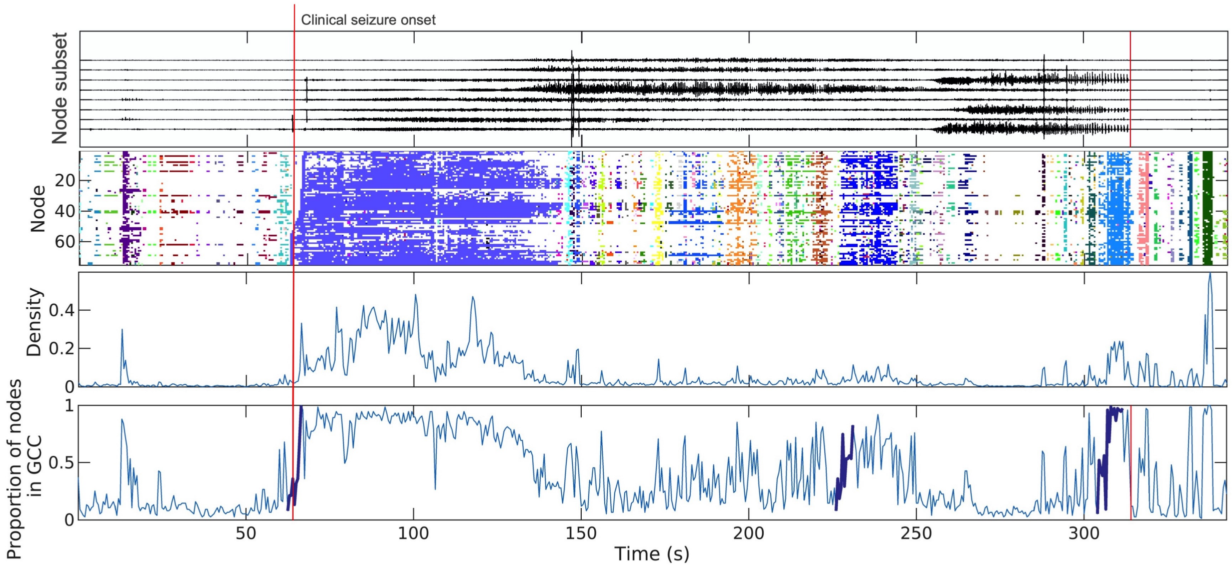

Figure 7.1: Seizure 1: left hemisphere. Two vertical lines represent seizure onset and terminations times, respectively. Top: Voltage time series recorded at eight electrodes on left brain hemisphere. Second row: Community membership (indicated by colors) for each node over time; the large dynamic communities manifest ROIs. Third row: Network density over time. Bottom: Proportion of nodes in the GCC over time; the bold curves in ROIs are chosen by automatic segment finder for testing.

	$\hat{p}$	$\hat{q}$	$\hat{\gamma}$	$\hat{\alpha}$	$\hat{\beta}$	$loglik$
ER	0.404 (0.112)	0.248 (0.075)	10.075 (1.423)	0.068 (0.0005)	0.435 (0.035)	-5636.822 (5.942)
PR	0.468 (0.156)	0.163 (0.072)	9.819 (1.512)	0.068 (0.0004)	0.464 (0.032)	-5647.120 (6.850)
log(BF)	10.297 (9.468))					

Table 7.1: Mean and standard deviation of parameter estimates for ER/PR process, log-likelihood estimates and Bayes factor (log scale) estimate based off of 10 trials for seizure 1 on the left brain hemisphere (first bold segment).

	$\hat{p}$	$\hat{q}$	$\hat{\gamma}$	$\hat{\alpha}$	$\hat{\beta}$	*loglik*
ER	0.580 (0.111)	0.279 (0.110)	6.480 (0.823)	0.033 (0.0004)	0.273 (0.047)	-4222.710 (15.563)
PR	0.625 (0.092)	0.250 (0.134)	6.010 (0.348)	0.033 (0.0004)	0.336 (0.040)	-4254.414 (11.118)
log(BF)	31.704 (15.141)					

Table 7.2: Mean and standard deviation of parameter estimates for ER/PR process, log-likelihood estimates and Bayes factor (log scale) estimate based off of 10 trials for seizure 1 on the left brain hemisphere (second bold segment).

	$\hat{p}$	$\hat{q}$	$\hat{\gamma}$	$\hat{\alpha}$	$\hat{\beta}$	*loglik*
ER	0.647 (0.051)	0.138 (0.055)	10.037 (0.631)	0.128 (0.0007)	0.214 (0.024)	-16058.532 (24.298)
PR	0.642 (0.037)	0.141 (0.078)	7.567 (1.209)	0.129 (0.0004)	0.228 (0.026)	-16120.504 (26.966)
log(BF)	61.971 (33.687)					

Table 7.3: Mean and standard deviation of parameter estimates for ER/PR process, log-likelihood estimates and Bayes factor (log scale) estimate based off of 10 trials for seizure 1 on the left brain hemisphere (third bold segment).

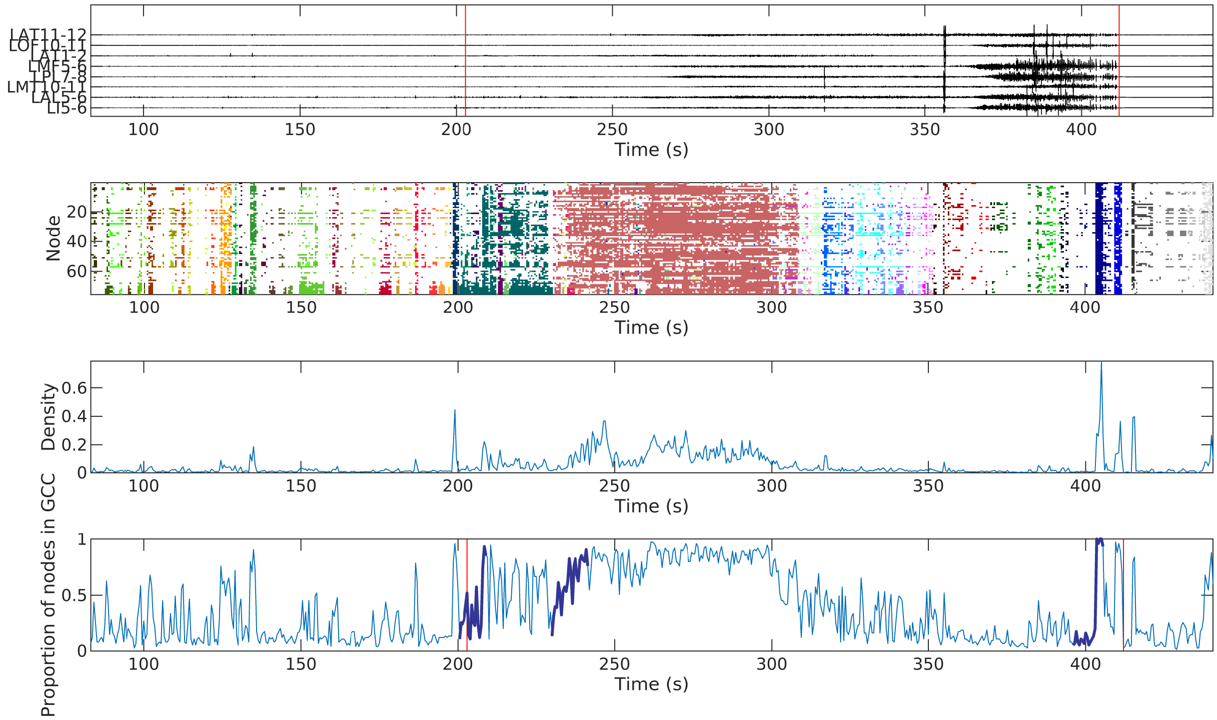

Figure 7.2: Seizure 2: left hemisphere. Two vertical lines represent seizure onset and terminations times, respectively. Top: Voltage time series recorded at eight electrodes on left brain hemisphere. Second row: Community membership (indicated by colors) for each node over time; the large dynamic communities manifest ROIs. Third row: Network density over time. Bottom: Proportion of nodes in the GCC over time; the bold curves in ROIs are chosen by automatic segment finder for testing.

	$\hat{p}$	$\hat{q}$	$\hat{\gamma}$	$\hat{\alpha}$	$\hat{\beta}$	*loglik*
ER	0.573 (0.051)	0.269 (0.075)	6.041 (0.223)	0.047 (0.0004)	0.262 (0.019)	-9226.559 (25.313)
PR	0.587 (0.057)	0.288 (0.103)	5.732 (0.242)	0.047 (0.0001)	0.279 (0.020)	-9277.567 (21.869)
log(BF)	51.008 (31.198)					

Table 7.4: Mean and standard deviation of parameter estimates for ER/PR process, log-likelihood estimates and Bayes factor (log scale) estimate based off of 10 trials for seizure 2 on the left brain hemisphere (first bold segment).

	$\hat{p}$	$\hat{q}$	$\hat{\gamma}$	$\hat{\alpha}$	$\hat{\beta}$	*loglik*
ER	0.522 (0.049)	0.258 (0.067)	8.846 (0.596)	0.081 (0.0007)	0.354 (0.031)	-18375.956 (52.389)
PR	0.545 (0.068)	0.277 (0.088)	7.159 (0.852)	0.081 (0.0009)	0.410 (0.037)	-18481.718 (40.650)
log(BF)	105.762 (73.922)					

Table 7.5: Mean and standard deviation of parameter estimates for ER/PR process, log-likelihood estimates and Bayes factor (log scale) estimate based off of 10 trials for seizure 2 on the left brain hemisphere (second bold segment).

	$\hat{p}$	$\hat{q}$	$\hat{\gamma}$	$\hat{\alpha}$	$\hat{\beta}$	*loglik*
ER	0.533 (0.026)	0.141(0.038)	8.292(0.548)	0.099 (0.0001)	0.150 (0.021)	-16234.315 (13.354)
PR	0.575 (0.079)	0.174 (0.082)	7.783 (0.568)	0.099 (0.0001)	0.177 (0.025)	-16241.436 (15.226)
log(BF)	7.119 (19.931)					

Table 7.6: Mean and standard deviation of parameter estimates for ER/PR process, log-likelihood estimates and Bayes factor (log scale) estimate based off of 10 trials for seizure 2 on the left brain hemisphere (third bold segment).

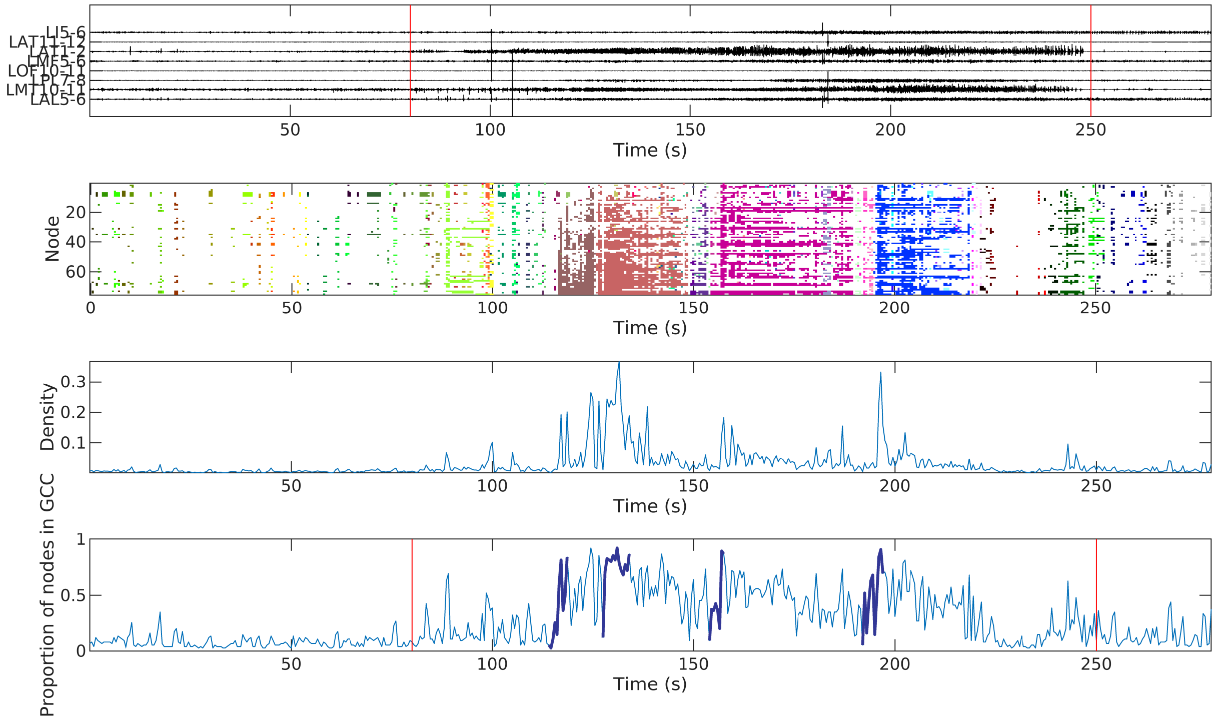

Figure 7.3: Seizure 3: left hemisphere. Two vertical lines represent seizure onset and terminations times, respectively. Top: Voltage time series recorded at eight electrodes on left brain hemisphere. Second row: Community membership (indicated by colors) for each node over time; the large dynamic communities manifest ROIs. Third row: Network density over time. Bottom: Proportion of nodes in the GCC over time; the bold curves in ROIs are chosen by automatic segment finder for testing.

	$\hat{p}$	$\hat{q}$	$\hat{\gamma}$	$\hat{\alpha}$	$\hat{\beta}$	*loglik*
ER	0.651 (0.042)	0.166 (0.084)	6.367 (0.625)	0.055 (0.0003)	0.200 (0.027)	-5388.566 (9.834)
PR	0.665 (0.040)	0.128 (0.079)	5.722 (0.341)	0.055 (0.0002)	0.225 (0.036)	-5399.512 (7.227)
log(BF)	10.945 (12.786)					

Table 7.7: Mean and standard deviation of parameter estimates for ER/PR process, log-likelihood estimates and Bayes factor (log scale) estimate based off of 10 trials for seizure 3 on the left brain hemisphere (first bold segment).

	$\hat{p}$	$\hat{q}$	$\hat{\gamma}$	$\hat{\alpha}$	$\hat{\beta}$	*loglik*
ER	0.824 (0.064)	0.091 (0.036)	12.746 (1.522)	0.198 (0.0006)	0.268 (0.023)	-18120.573 (25.416)
PR	0.682 (0.112)	0.134 (0.077)	7.442 (2.783)	0.201 (0.0007)	0.219 (0.071)	-18182.074 (22.634)
log(BF)	61.503 (25.172)					

Table 7.8: Mean and standard deviation of parameter estimates for ER/PR process, log-likelihood estimates and Bayes factor (log scale) estimate based off of 10 trials for seizure 3 on the left brain hemisphere (second bold segment).

	$\hat{p}$	$\hat{q}$	$\hat{\gamma}$	$\hat{\alpha}$	$\hat{\beta}$	*loglik*
ER	0.462 (0.078)	0.217(0.125	7.629 (0.643)	0.053 (0.0003)	0.185 (0.027)	-4102.365 (17.419)
PR	0.535 (0.126)	0.241(0.151)	6.961 (0.616)	0.053 (0.0004)	0.259 (0.070)	-4117.433 (5.860)
log(BF)	15.068 (20.097)					

Table 7.9: Mean and standard deviation of parameter estimates for ER/PR process, log-likelihood estimates and Bayes factor (log scale) estimate based off of 10 trials for seizure 3 on the left brain hemisphere (third bold segment).

	$\hat{p}$	$\hat{q}$	$\hat{\gamma}$	$\hat{\alpha}$	$\hat{\beta}$	*loglik*
ER	0.629 (0.091)	0.154 (0.070)	7.700 (0.586)	0.076 (0.0003)	0.179 (0.059)	-6836.452 (6.823)
PR	0.674 (0.035)	0.119 (0.051)	6.660 (0.569)	0.076 (0.0003)	0.248 (0.031)	-6831.450 (7.429)
log(BF)	-5.002 (7.224)					

Table 7.10: Mean and standard deviation of parameter estimates for ER/PR process, log-likelihood estimates and Bayes factor (log scale) estimate based off of 10 trials for seizure 3 on the left brain hemisphere (fourth bold segment).

8 Robustness of Results to Network Construction and Segment Identification

To explore the robustness of the testing results on percolation regimes to the functional connectivity network construction from the ECoG data and the subsequent segment identification, we construct the network time series using a different threshold value ($q = 0.05$) and rerun the testing procedure on the resulting networks for each of the three seizures. Recall that each snapshot in the network time series is inferred by measuring the coupling strength between pairs of nodes within a sliding window of time and then assigning edges where coupling is judged to be sufficiently strong. Following the network construction method in [7], we assess coupling strength on node pairs through multiple statistical hypothesis tests and declare edges between node pairs whose test is assessed to be significant under a pre-specified FDR level, denoted as q. The FDR level can be regarded as the threshold in our network construction. While all results in the main text are from the networks constructed under $q = 0.5$, in this section, we provide a counterpart of the results using networks constructed under $q = 0.05$. Figure 8.1, 8.2 and 8.3 show, respectively, for the three seizures, the resulting network density/GCC over time, the dynamic communities detected using the dynamic plex percolation method (DPPM) proposed in [4], and the segment chosen in ROIs where the large dynamic community appears. Table 8.1-8.6 show the estimation and testing results for each segment identified.

In contrast to the three plots in sections 7, we can spot several differences in the newly constructed networks: 1) the network density reduces in that fewer edges are claimed in lowering the level of FDR; 2) the network dynamics in topology changes, but not substantially as there are many similar patterns; 3) the dynamic communities detected from the newly constructed network time series remain similar for some time periods in the first seizure, but change prominently for the other two seizures. Given that the ROIs where segments are identified for percolation regime testing correspond with the dynamic communities detected, the prominent change in ROIs leads to the change in the segments identified for testing.

Specifically, for the first seizure, three segments are selected for testing. Two are at similar places as before, whereas one appears at a new place. Similarly, for the second seizure, one segment selected is at a similar place as before, while the other one is at a new place. Regardless of the change in segment identification, the Bayes factors in log scale are positive for all segments in seizure 1 and seizure 2, which are consistent with our conclusions drawn in the main text based off of networks constructed under $q = 0.5$. For seizure 3, all large dynamic communities detected in the previous networks are missing in the newly constructed networks. There seems to be only one legitimate community detected near time 130, but of which both size and duration shrink significantly compared with what is obtained in the previous networks. The Bayes factor in log scale for the segment identified in this ROI is positive, which is consistent with our finding in the main text at the similar place. However, under the newly constructed networks, the segment around time 190, where we find signal for PR percolation from the previous networks, is not identified as a qualified segment for testing, as the dynamic community detected around this time is small and transient.

In conclusion, from this robustness check of limited scope, our findings on percolation regimes for seizure 1 and 2 are quite robust to the network constructions. However, it seems that for seizure 3, the finding on the evidence of PR percolation at the late ictal stage is sensitive to the network construction, caused by the ROI and segment identification being sensitive to the network construction for this seizure.

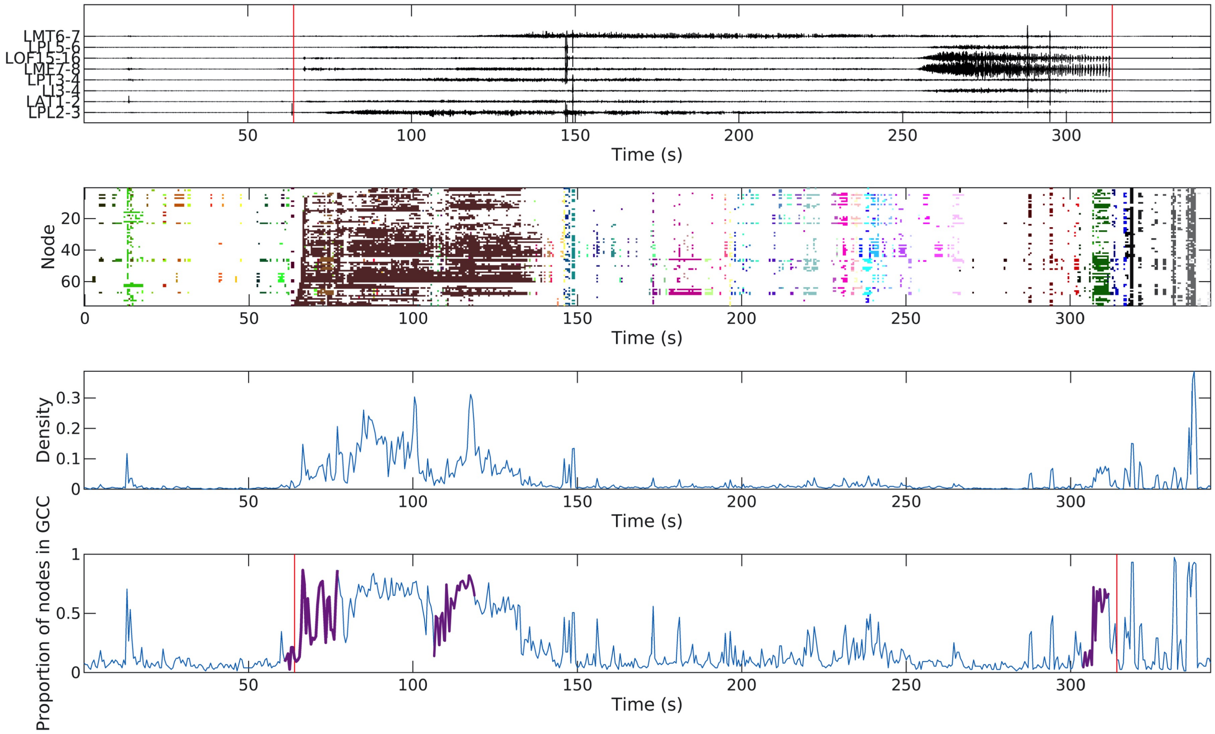

Figure 8.1: Robustness check on seizure 1: left hemisphere. Two vertical lines represent seizure onset and terminations times, respectively. Top: Voltage time series recorded at eight electrodes on left brain hemisphere. Second row: Community membership (indicated by colors) for each node over time; the large dynamic communities manifest ROIs. Third row: Network density over time. Bottom: Proportion of nodes in the GCC over time; the bold curves in ROIs are chosen by automatic segment finder for testing.

	$\hat{p}$	$\hat{q}$	$\hat{\gamma}$	$\hat{\alpha}$	$\hat{\beta}$	*loglik*
ER	0.580 (0.057)	0.285 (0.061)	7.212 (0.611)	0.051 (0.0005)	0.309 (0.065)	-16653.275 (46.676)
PR	0.620 (0.054)	0.289 (0.082)	6.507 (0.461)	0.052 (0.0003)	0.349 (0.068)	-16770.964 (59.729)
log(BF)	117.692 (88.266)					

Table 8.1: Robustness check on seizure 1. Mean and standard deviation of parameter estimates for ER/PR process, log-likelihood estimates and Bayes factor (log scale) estimate based off of 10 trials for seizure 1 on the left brain hemisphere (first bold segment).

	$\hat{p}$	$\hat{q}$	$\hat{\gamma}$	$\hat{\alpha}$	$\hat{\beta}$	*loglik*
ER	0.563 (0.039)	0.194 (0.062)	8.283 (0.487)	0.088 (0.0004)	0.206 (0.024)	-20270.652 (54.602)
PR	0.543 (0.033)	0.156 (0.103)	6.056 (0.390)	0.089 (0.0004)	0.227 (0.027)	-20341.156 (59.137)
log(BF)	70.505 (90.591)					

Table 8.2: Robustness check on seizure 1. Mean and standard deviation of parameter estimates for ER/PR process, log-likelihood estimates and Bayes factor (log scale) estimate based off of 10 trials for seizure 1 on the left brain hemisphere (second bold segment).

	$\hat{p}$	$\hat{q}$	$\hat{\gamma}$	$\hat{\alpha}$	$\hat{\beta}$	*loglik*
ER	0.719 (0.062)	0.171 (0.100)	5.762 (0.602)	0.033 (0.0002)	0.129 (0.074)	-5405.591 (22.349)
PR	0.663 (0.053)	0.185 (0.080)	5.593 (0.490)	0.034 (0.0002)	0.160 (0.053)	-5436.392 (13.467)
log(BF)	30.801 (17.153)					

Table 8.3: Robustness check on seizure 1. Mean and standard deviation of parameter estimates for ER/PR process, log-likelihood estimates and Bayes factor (log scale) estimate based off of 10 trials for seizure 1 on the left brain hemisphere (third bold segment).

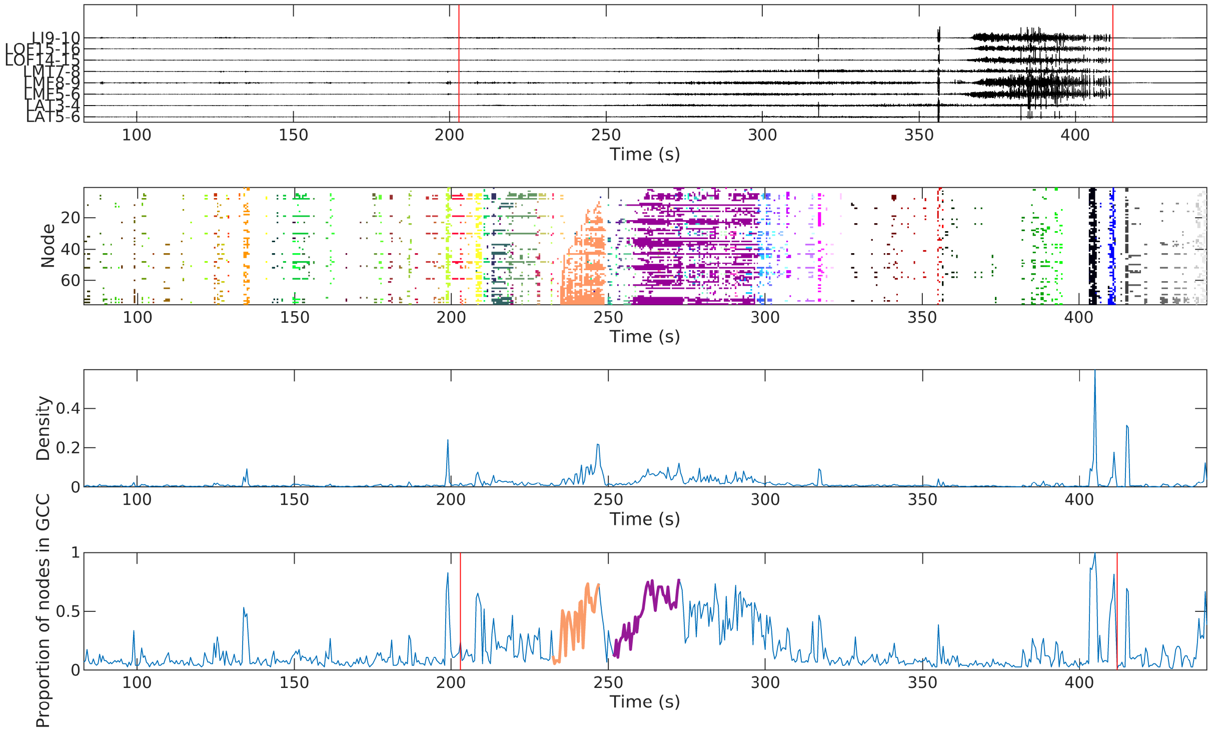

Figure 8.2: Robustness check on seizure 2. Seizure 2: left hemisphere. Two vertical lines represent seizure onset and terminations times, respectively. Top: Voltage time series recorded at eight electrodes on left brain hemisphere. Second row: Community membership (indicated by colors) for each node over time; the large dynamic communities manifest ROIs. Third row: Network density over time. Bottom: Proportion of nodes in the GCC over time; the bold curves in ROIs are chosen by automatic segment finder for testing.

	$\hat{p}$	$\hat{q}$	$\hat{\gamma}$	$\hat{\alpha}$	$\hat{\beta}$	*loglik*
ER	0.465 (0.037)	0.214 (0.047)	7.416 (0.447)	0.054 (0.0004)	0.311 (0.017)	-17321.240 (41.432)
PR	0.469 (0.046)	0.270 (0.081)	6.293 (0.429)	0.055 (0.0002)	0.368 (0.040)	-17544.988 (53.852)
log(BF)	223.747 (51.887)					

Table 8.4: Robustness check on seizure 2. Mean and standard deviation of parameter estimates for ER/PR process, log-likelihood estimates and Bayes factor (log scale) estimate based off of 10 trials for seizure 2 on the left brain hemisphere (first bold segment).

	$\hat{p}$	$\hat{q}$	$\hat{\gamma}$	$\hat{\alpha}$	$\hat{\beta}$	*loglik*
ER	0.614 (0.032)	0.324 (0.047)	6.907 (0.394)	0.039 (0.0004)	0.293 (0.025)	-20050.159 (145.434)
PR	0.581 (0.032)	0.300 (0.040)	5.962 (0.240)	0.041 (0.0004)	0.352 (0.036)	-20567.737 (89.088)
log(BF)	517.579 (185.938)					

Table 8.5: Robustness check on seizure 2. Mean and standard deviation of parameter estimates for ER/PR process, log-likelihood estimates and Bayes factor (log scale) estimate based off of 10 trials for seizure 2 on the left brain hemisphere (second bold segment).

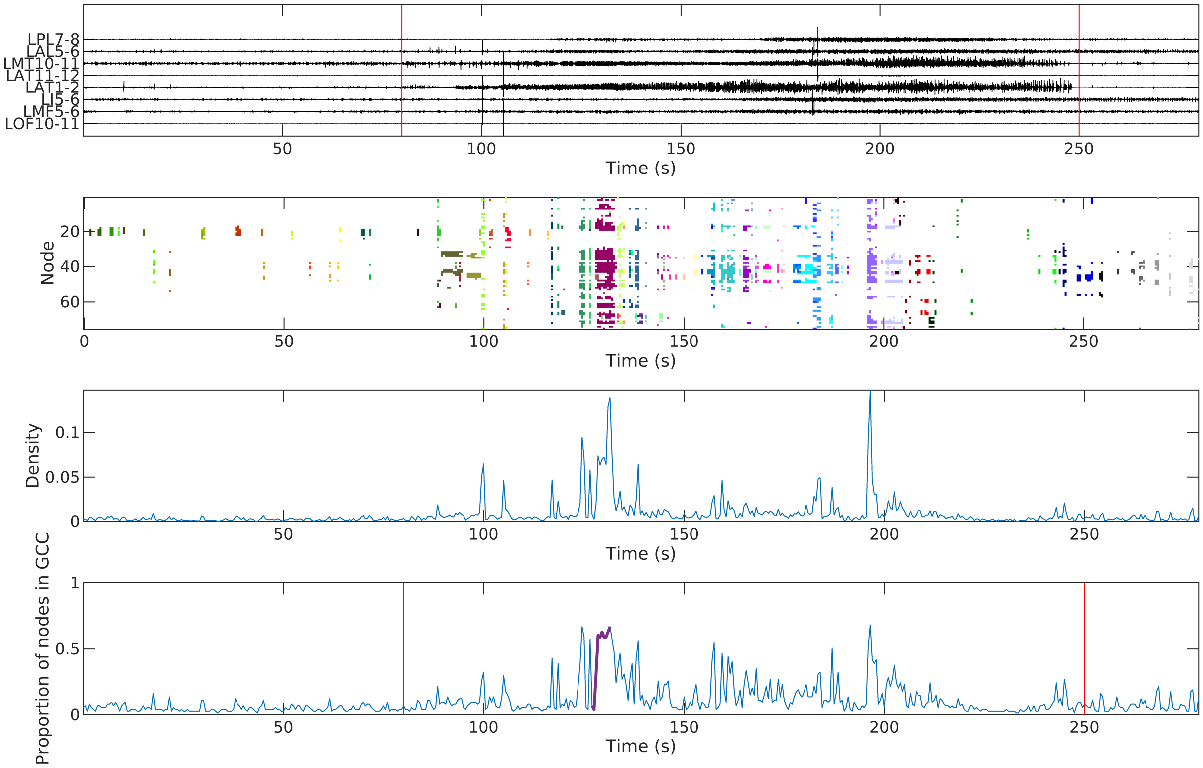

Figure 8.3: Robustness check on seizure 3. Seizure 3: left hemisphere. Two vertical lines represent seizure onset and terminations times, respectively. Top: Voltage time series recorded at eight electrodes on left brain hemisphere. Second row: Community membership (indicated by colors) for each node over time; the large dynamic communities manifest ROIs. Third row: Network density over time. Bottom: Proportion of nodes in the GCC over time; the bold curves in ROIs are chosen by automatic segment finder for testing.

	$\hat{p}$	$\hat{q}$	$\hat{\gamma}$	$\hat{\alpha}$	$\hat{\beta}$	*loglik*
ER	0.706 (0.147)	0.089 (0.052)	7.394 (1.144)	0.063 (0.0006)	0.209 (0.043)	-5990.761 (23.376)
PR	0.607 (0.058)	0.105 (0.093)	5.433 (0.365)	0.064 (0.0003)	0.214 (0.030)	-6050.294 (8.423)
log(BF)	59.533 (22.619)					

Table 8.6: Robustness check on seizure 3. Mean and standard deviation of parameter estimates for ER/PR process, log-likelihood estimates and Bayes factor (log scale) estimate based off of 10 trials for seizure 3 on the left brain hemisphere.

9 Goodness of Fit for RG-HMM

The most commonly used approach for assessing goodness-of-fit of network models is to (i) use Monte Carlo methods to randomly generate from the fitted model and then (ii) compute a particular set of statistical summaries on those networks generated for comparison with those same summaries on the observed networks. This type of numerical approach is heavily used for assessing goodness-of-fit in exponential random graph models (ERGMs) [8] and is referred to as a graphical test of goodness-of-fit. In this section, we adopt this graphical test approach to evaluate how well our models fit the observed networks constructed from the real seizure data. Specifically, for each fitted model on each segment identified from each seizure, we generate 500 network time series from the RG-HMM with the estimated parameters under the inferred percolation process. We compare these generated networks with the observed seizure networks on three structural aspects of networks: the size of giant connected component (GCC), the size of the second largest connected component (SLC), and the density.

One adjustment we made in generating networks from the fitted model is that we scale down the type-I error rate estimate ($\hat{\alpha}$) by a factor of 0.3. This is because our earlier simulation results (described in the Results section of the main text) indicate that our estimation algorithm (based on particle filtering) tends to overestimate the error rate parameters when a limited number of particles is used out of consideration for computational feasibility, and the percolation curve is sensitive to the type-I error rate. The factor value 0.3 is chosen based on our experience in the simulation study.

Figure 9.1 shows a comparison of the Monte Carlo results for one segment in seizure 1 – one with the type-I error adjusted to account for overestimation, the other without. We can see that without accounting for overestimation in α the size of GCC is completely off under the overestimated error rate, the size of SLC is also a bit off, and the density is less affected. This is as expected in that the false positive edges in the observed networks introduced by a small type-I error rate typically impact the connected components the most and the network density the least. Accounting for overestimation in α, the behavior of the generated networks and the actual seizure networks is qualitatively quite similar for much of the time period.

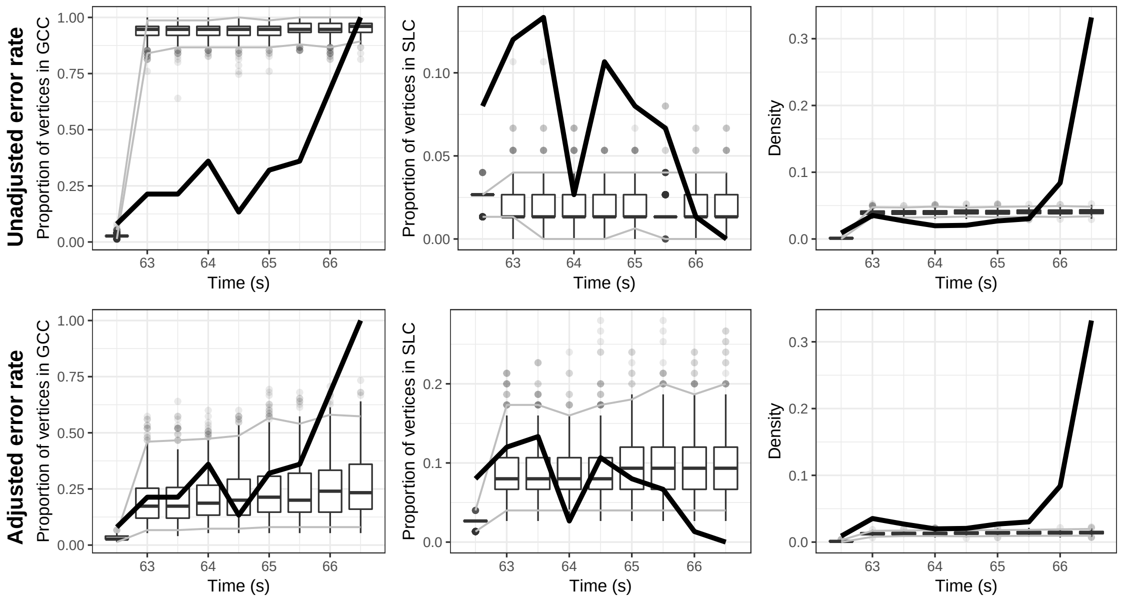

Figure 9.1: Comparison on the simulation results with and without adjusting type-I error rate to account for overestimation. The boxplot at each time point consisting of median and interquartile range represents the distribution of the size of GCC/SLC and density on simulated networks. The light gray lines represent the range in which 95% of the simulated networks fall. *Top*: Three goodness-of-fit plots with unadjusted error rate, for one segment in seizure 1 on GCC, SLC and density, respectively. *Bottom*: The same three goodness-of-fit plots with adjusted error rate.

Figures 9.2, 9.3 and 9.4 are three goodness-of-fit plots, which depict comparison between Monte Carlo and observed networks for the three network characteristics, respectively. Boxplots are used to show empirical distributions of the summary statistics of the generated networks at each observation time point. The solid line represents the statistics for the observed networks from the seizure data, and the light gray lines represent the range in which 95% of the simulated networks fall.

We can see that the observed curve largely falls within the 95% range of the generated networks for the majority of the seizure segments, although there are a few plots where the seizure curve partially falls out of the range. Overall, the fitted ER/PR model does a respectable job of recreating the GCC/SLC/density dynamics in the selected seizure segments – particularly in light of how simple these models are compared to the complexity of the underlying physiological processes.

Meanwhile, we can observe that the quality of resulting fit differs when assessed under different network statistics. The model appears to fit the seizure data better from the perspective of the GCC and SLC compared to the density. Since connected components play a key role in percolation models, we think GCC and SLC are more important in assessing fit than density. Therefore, based on this graphical assessment of goodness-of-fit, the ER/PR percolation model looks to fit the seizure data decently well.

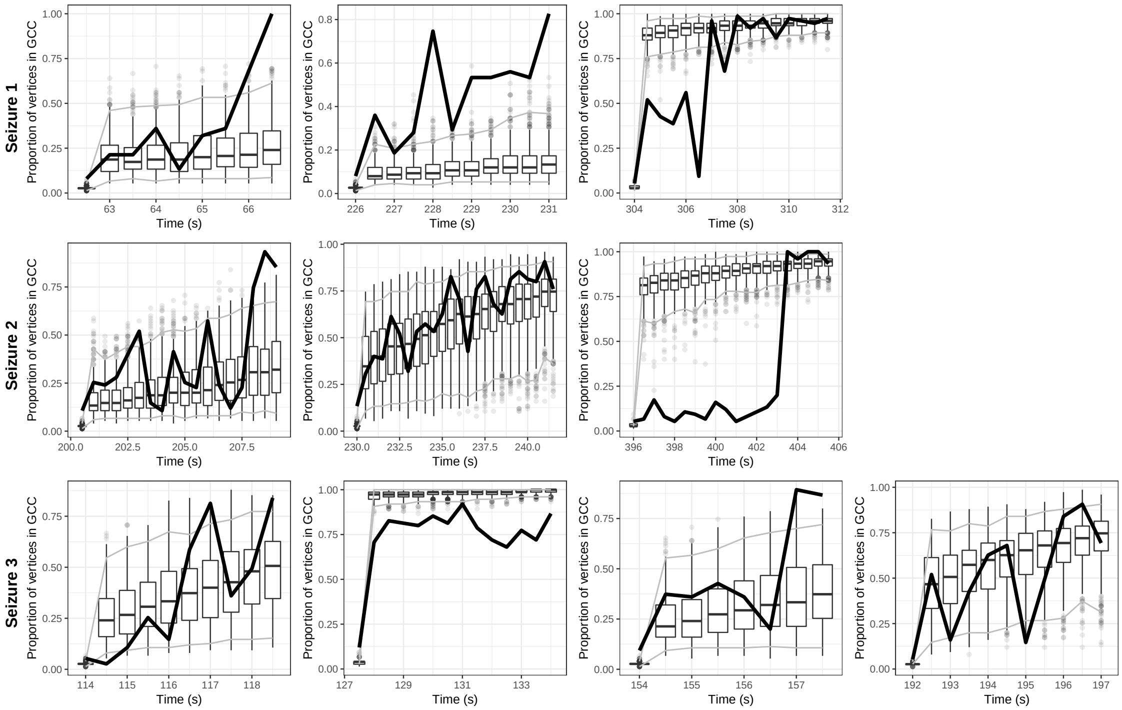

Figure 9.2: Goodness-of-fit plot: comparison of the simulated and observed networks on the size of GCC. The solid line represents the observed seizure networks. The boxplot at each time point consisting of median and interquartile range represents the distribution of the size of GCC on simulated networks. The light gray lines represent the range in which 95% of the simulated networks fall.

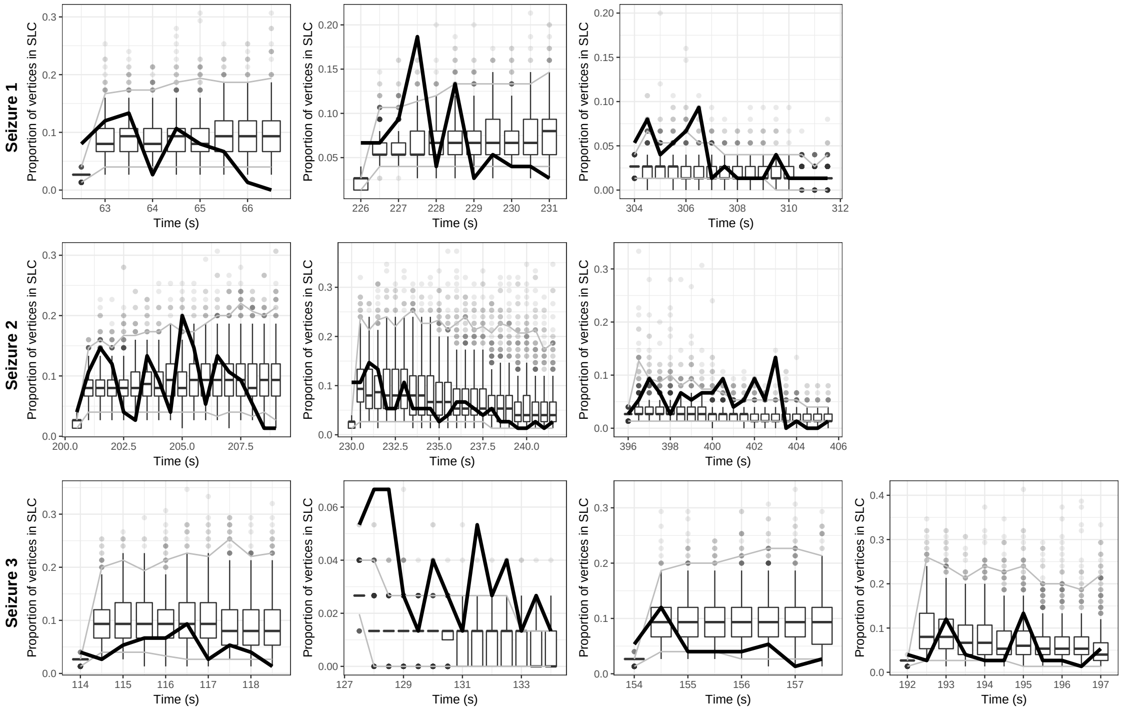

Figure 9.3: Goodness-of-fit plot: comparison of the simulated and observed networks on the size of SLC. The solid line represents the observed seizure networks. The boxplot at each time point consisting of median and interquartile range represents the distribution of the size of SLC on simulated networks. The light gray lines represent the range in which 95% of the simulated networks fall.

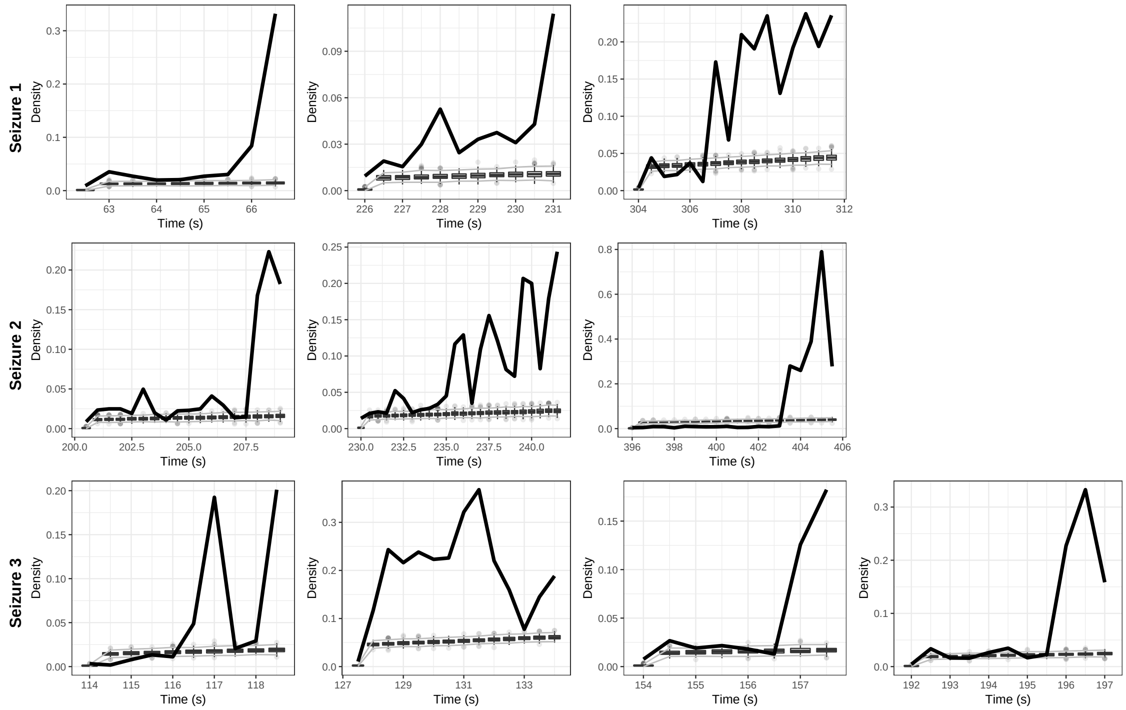

Figure 9.4: Goodness-of-fit plot: comparison of the simulated and observed networks on the size of density. The solid line represents the observed seizure networks. The boxplot at each time point consisting of median and interquartile range represents the distribution of the density on simulated networks. The light gray lines represent the range in which 95% of the simulated networks fall.

Appendices

A PMF of Feasible Sample Path

The probability function of feasible sample path $\boldsymbol{S}_1, \boldsymbol{V}_1$ conditional on $G(t_1), W(t_1)$ is

$$
\begin{aligned}
& p_{sp}\Big(\boldsymbol{s}_1, \boldsymbol{v}_1 \big| g(t_1), w(t_1)) \\
&= P\Big(\boldsymbol{S}_1 = \big(g(\tau_1), g(\tau 2), ..., g(\tau_{R_1})\big), \boldsymbol{V}_1 = \big(w(\tau_1), w(\tau 2), ..., w(\tau_{R_1})\big) \big| G(t_1) = g(t_1), \\
&\qquad W(t_1) = w(t_1)\Big) \cdot P(\tau_{R_1} \le t_2 < \tau_{R_1+1} \big| G(t_1) = g(t_1), W(t_1) = w(t_1), \boldsymbol{S}_1 = \boldsymbol{s}_1, \boldsymbol{V}_1 = \boldsymbol{v}_1) \\
&= \prod_{r=1}^{R_1} P\Big(G(\tau_r) = g(\tau_r), W(\tau_r) = w(\tau_r) \big| G(\tau_{r-1}) = g(\tau_{r-1}), W(\tau_{r-1}) = w(\tau_{r-1})\Big) \\
&\qquad \cdot P(\tau_{R_1} \le t_2 < \tau_{R_1+1} \big| G(t_1) = g(t_1), W(t_1) = w(t_1), \boldsymbol{S}_1 = \boldsymbol{s}_1, \boldsymbol{V}_1 = \boldsymbol{v}_1) \\
&= \prod_{r=1}^{R_1} f\Big(g(\tau_r), w(\tau_r) \big| g(\tau_{r-1}), w(\tau_{r-1})\Big) \\
&\qquad \cdot P(\tau_{R_1} \le t_2 < \tau_{R_1+1} \big| G(t_1) = g(t_1), W(t_1) = w(t_1), \boldsymbol{S}_1 = \boldsymbol{s}_1, \boldsymbol{V}_1 = \boldsymbol{v}_1) \\
&= \prod_{r=1}^{R_1} g\Big(g(\tau_r) \big| w(\tau_r), g(\tau_{r-1})\Big) h\Big(w(\tau_r) \big| g(\tau_{r-1}), w(\tau_{r-1})\Big) \\
&\qquad \cdot P(\tau_{R_1} \le t_2 < \tau_{R_1+1} \big| G(t_1) = g(t_1), W(t_1) = w(t_1), \boldsymbol{S}_1 = \boldsymbol{s}_1, \boldsymbol{V}_1 = \boldsymbol{v}_1),
\end{aligned}
\tag{A.1}
$$

where

$$
\begin{aligned}
& P(\tau_{R_1} \le t_2 < \tau_{R_1+1} \big| G(t_1) = g(t_1), W(t_1) = w(t_1), \boldsymbol{S}_1 = \boldsymbol{s}_1, \boldsymbol{V}_1 = \boldsymbol{v}_1) \\
&= P(R_1 \text{ transitions between } (t_1, t_2]) \\
&= \exp(-\gamma(t_2 - t_1)) \frac{(\gamma(t_2 - t_1))^{R_1}}{R_1!} \qquad \text{since } R_1 \sim poisson(\gamma(t_2 - t_1)).
\end{aligned}
\tag{A.2}
$$

Therefore,

$$
\begin{aligned}
& p_{sp}\Big(\boldsymbol{s}_1, \boldsymbol{v}_1 \big| g(t_1), w(t_1)) \\
&= \exp(-\gamma(t_2 - t_1)) \frac{(\gamma(t_2 - t_1))^{R_1}}{R_1!} \cdot \prod_{r=1}^{R_1} g\Big(g(\tau_r) \big| w(\tau_r), g(\tau_{r-1})\Big) h\Big(w(\tau_r) \big| g(\tau_{r-1}), w(\tau_{r-1})\Big),
\end{aligned}
\tag{A.3}
$$

where $\boldsymbol{s}_1 = (g(\tau_1), g(\tau_2), ..., g(\tau_R)), \boldsymbol{v}_1 = (w(\tau_1), w(\tau_2), ..., w(\tau_R)), \tau_0 = t_1$, $g(\cdot \big| \cdot)$ is the conditional pmf of r.v. $G(\tau_2)$ given $\big(W(\tau_2), G(\tau_1)\big)$, and $h(\cdot \big| \cdot)$ is the conditional pmf of r.v. $W(\tau_2)$ given $\big(G(\tau_1), W(\tau_1)\big)$. τ_1, τ_2 are two arbitrary consecutive transition time points, $\tau_1 < \tau_2$. More details on $g(\cdot \big| \cdot)$ and $h(\cdot \big| \cdot)$on are provided in Appendix B.

Generally, for $m = 2, 3, ..., M$, the pmf of feasible sample path $\boldsymbol{S}_{m-1}, \boldsymbol{V}_{m-1}$ that brings $g(t_{m-1})$

to $g(t_m)$ conditional on $G(t_{m-1}), W(t_{m-1})$ is

$$\begin{aligned} & p_{sp}\Big(\boldsymbol{s}_{m-1}, \boldsymbol{v}_{m-1}\big|g(t_{m-1}), w(t_{m-1})\big) \\ &= \exp(-\gamma(t_m - t_{m-1}))\frac{(\gamma(t_m - t_{m-1}))^{R_{m-1}}}{R_{m-1}!} \\ &\cdot \prod_{r=1}^{R_{m-1}} g\Big(g(\tau_r^{(m-1)})\big|w(\tau_r^{(m-1)}), g(\tau_{r-1}^{(m-1)})\Big)h\Big(w(\tau_r^{(m-1)})\big|g(\tau_{r-1}^{(m-1)}), w(\tau_{r-1}^{(m-1)})\Big), \end{aligned} \tag{A.4}$$

where $\boldsymbol{s}_{m-1} = (g(\tau_1^{(m-1)}), g(\tau_2^{(m-1)}), ..., g(\tau_{R_{m-1}}^{(m-1)})), \boldsymbol{v}_{m-1} = (w(\tau_1^{(m-1)}), w(\tau_2^{(m-1)}), ..., w(\tau_{R_{m-1}}^{(m-1)}))$ is the sample path from $g(t_{m-1})$ to $g(t_m)$ between t_{m-1}, and t_m; $(\tau_1^{(m-1)}, ..., \tau_{R_{m-1}}^{(m-1)})$ are the R_{m-1} transition time points between t_{m-1} and t_m; $\tau_0^{(m-1)} = t_{m-1}$.

B Transition Probability for Percolation Models

Recall that $\mathcal{X} = \{0,1\} \times \{1,2,3,\cdots,2^{\binom{N}{2}}\}$, where N is the number of nodes in networks. The one-step transition probability for the network-valued Markov chain, $\{\big(G(t), W(t)\big) \in \mathcal{X}, t_1 \le t \le t_M\}$, from (g_1, w_1) to (g_2, w_2) in a single step is

$$\begin{aligned} & P\Big(\big(G(\tau_2), W(\tau_2)\big) = \big(g_2, w_2\big)\big|\big(G(\tau_1), W(\tau_1)\big) = \big(g_1, w_1\big)\Big) \\ &= P\Big(G(\tau_2) = g_2, W(\tau_2) = w_2\big|G(\tau_1) = g_1, W(\tau_1) = w_1\Big) \\ &= P\Big(G(\tau_2) = g_2\big|W(\tau_2) = w_2, G(\tau_1) = g_1, W(\tau_1) = w_1\Big)\cdot \\ &\qquad\qquad P\Big(W(\tau_2) = w_2\big|G(\tau_1) = g_1, W(\tau_1) = w_1\Big) \\ &= P\Big(G(\tau_2) = g_2\big|W(\tau_2) = w_2, G(\tau_1) = g_1\Big)\cdot P\Big(W(\tau_2) = w_2\big|G(\tau_1) = g_1, W(\tau_1) = w_1\Big) \\ &\quad (\text{since } G(\tau_2) \perp\!\!\!\perp W(\tau_1)\big|G(\tau_1), W(\tau_2)), \end{aligned} \tag{B.1}$$

where τ_1, τ_2 are two arbitrary consecutive transition time points, $\tau_1 < \tau_2$. The first probability is specified by the percolation model, specifically by the way of choosing edge to add or delete, and the second probability is given in Table 1 in the main text.

We can rewrite the one-step transition probability as

$$f(g_2, w_2\big|g_1, w_1) = g(g_2\big|w_2, g_1)\cdot h(w_2\big|g_1, w_1), \tag{B.2}$$

where $f(\cdot\big|\cdot)$ is the conditional pmf of rvs $\big(G(\tau_2), W(\tau_2)\big)$ given $\big(G(\tau_1), W(\tau_1)\big)$, $g(\cdot\big|\cdot)$ is the conditional pmf of rv. $G(\tau_2)$ given $\big(W(\tau_2), G(\tau_1)\big)$, and $h(\cdot\big|\cdot)$ is the conditional pmf of rv. $W(\tau_2)$ given $\big(G(\tau_1), W(\tau_1)\big)$. τ_1, τ_2 are two arbitrary consecutive transition time points, $\tau_1 < \tau_2$.

Specifically, $g(g_2\big|w_2, g_1)$ is parameter-free, only depend on edge change rule in the percolation model.

$$\begin{aligned} h(w_2\big|g_1, w_1) &= h_{p,q}(w_2\big|g_1, w_1) \\ &= w_2\mathbf{1}_{\{g_1=\text{Empty}\}} + (1-w_2)\mathbf{1}_{\{g_1=\text{Complete}\}} + \\ &\big(p^{w_2}(1-p)^{1-w_2}\mathbf{1}_{\{w_1=0\}} + (1-q)^{w_2}q^{1-w_2}\mathbf{1}_{\{w_1=1\}}\big)\mathbf{1}_{\{g_1\neq\text{Empty or Complete}\}}. \end{aligned} \tag{B.3}$$

C Proof of Missing Information Principle

Recall that $\boldsymbol{\theta} = (p, q, \gamma)$, we prove below that

$$S(\boldsymbol{\theta}; \boldsymbol{w}_{2:M}, \boldsymbol{g}_{2:M}) = E\big[S(\boldsymbol{\theta}; \boldsymbol{w}_{2:M}, \boldsymbol{g}_{2:M}, \boldsymbol{S}, \boldsymbol{V})\big|\boldsymbol{W}_{2:M} = \boldsymbol{w}_{2:M}, \boldsymbol{G}_{2:M} = \boldsymbol{g}_{2:M}\big] \tag{C.1}$$

Proof.

$$\begin{aligned}
Right &= \sum_{\boldsymbol{s},\boldsymbol{v}} \frac{\partial}{\partial \boldsymbol{\theta}} \log f_{\boldsymbol{\theta}}(\boldsymbol{w}, \boldsymbol{g}, \boldsymbol{s}, \boldsymbol{v}) \cdot f(\boldsymbol{s}, \boldsymbol{v}|\boldsymbol{w}, \boldsymbol{g}) \\
&= \sum_{\boldsymbol{s},\boldsymbol{v}} \frac{\partial}{\partial \boldsymbol{\theta}} \left(\log f_{\boldsymbol{\theta}}(\boldsymbol{s}, \boldsymbol{v} \mid \boldsymbol{w}, \boldsymbol{g}) + \log f_{\boldsymbol{\theta}}(\boldsymbol{w}, \boldsymbol{g})\right) \cdot f(\boldsymbol{s}, \boldsymbol{v}|\boldsymbol{w}, \boldsymbol{g}) \\
&= \sum_{\boldsymbol{s},\boldsymbol{v}} \frac{\partial}{\partial \boldsymbol{\theta}} \log f_{\boldsymbol{\theta}}(\boldsymbol{s}, \boldsymbol{v} \mid \boldsymbol{w}, \boldsymbol{g}) \cdot f(\boldsymbol{s}, \boldsymbol{v}|\boldsymbol{w}, \boldsymbol{g}) + \sum_{\boldsymbol{s},\boldsymbol{v}} \frac{\partial}{\partial \boldsymbol{\theta}} \log f_{\boldsymbol{\theta}}(\boldsymbol{w}, \boldsymbol{g}) \cdot f(\boldsymbol{s}, \boldsymbol{v}|\boldsymbol{w}, \boldsymbol{g}) \qquad \text{(C.2)} \\
&= 0 + \frac{\partial}{\partial \boldsymbol{\theta}} \log f_{\boldsymbol{\theta}}(\boldsymbol{w}, \boldsymbol{g}) \sum_{\boldsymbol{s},\boldsymbol{v}} f(\boldsymbol{s}, \boldsymbol{v}|\boldsymbol{w}, \boldsymbol{g}) \\
&= 0 + \frac{\partial}{\partial \boldsymbol{\theta}} \log f_{\boldsymbol{\theta}}(\boldsymbol{w}, \boldsymbol{g}) \cdot 1 = Left
\end{aligned}$$

Notice that $\sum_{\boldsymbol{s},\boldsymbol{v}} \frac{\partial}{\partial \boldsymbol{\theta}} \log f_{\boldsymbol{\theta}}(\boldsymbol{s}, \boldsymbol{v} \mid \boldsymbol{w}, \boldsymbol{g}) \cdot f(\boldsymbol{s}, \boldsymbol{v}|\boldsymbol{w}, \boldsymbol{g}) = \sum_{\boldsymbol{s},\boldsymbol{v}} \frac{\frac{\partial}{\partial \boldsymbol{\theta}} f_{\boldsymbol{\theta}}(\boldsymbol{s},\boldsymbol{v}|\boldsymbol{w},\boldsymbol{g})}{f_{\boldsymbol{\theta}}(\boldsymbol{s},\boldsymbol{v}|\boldsymbol{w},\boldsymbol{g})} \cdot f(\boldsymbol{s}, \boldsymbol{v}|\boldsymbol{w}, \boldsymbol{g}) = \sum_{\boldsymbol{s},\boldsymbol{v}} \frac{\partial}{\partial \boldsymbol{\theta}} f_{\boldsymbol{\theta}}(\boldsymbol{s}, \boldsymbol{v}|\boldsymbol{w}, \boldsymbol{g}) = \frac{\partial}{\partial \boldsymbol{\theta}} \sum_{\boldsymbol{s},\boldsymbol{v}} f_{\boldsymbol{\theta}}(\boldsymbol{s}, \boldsymbol{v}|\boldsymbol{w}, \boldsymbol{g}) = \frac{\partial}{\partial \boldsymbol{\theta}} 1 = 0$

References

[1] T. A. Snijders, J. Koskinen, and M. Schweinberger, "Maximum likelihood estimation for social network dynamics," *The Annals of Applied Statistics*, vol. 4, no. 2, p. 567, 2010.

[2] A. Doucet, N. De Freitas, and N. Gordon, "An introduction to sequential monte carlo methods," in *Sequential Monte Carlo methods in practice*, pp. 3–14, Springer, 2001.

[3] N. Chopin *et al.*, "Central limit theorem for sequential monte carlo methods and its application to bayesian inference," *The Annals of Statistics*, vol. 32, no. 6, pp. 2385–2411, 2004.

[4] L.-E. Martinet, M. Kramer, W. Viles, L. Perkins, E. Spencer, C. Chu, S. Cash, and E. Kolaczyk, "Robust dynamic community detection with applications to human brain functional networks," *Nature Communications*, vol. 11, no. 1, pp. 1–13, 2020.

[5] P. J. Bickel, Y. Ritov, T. Ryden, *et al.*, "Asymptotic normality of the maximum-likelihood estimator for general hidden markov models," *The Annals of Statistics*, vol. 26, no. 4, pp. 1614–1635, 1998.

[6] R. Douc, C. Matias, *et al.*, "Asymptotics of the maximum likelihood estimator for general hidden markov models," *Bernoulli*, vol. 7, no. 3, pp. 381–420, 2001.

[7] M. A. Kramer, U. T. Eden, S. S. Cash, and E. D. Kolaczyk, "Network inference with confidence from multivariate time series," *Physical Review E*, vol. 79, no. 6, p. 061916, 2009.

[8] D. R. Hunter, S. M. Goodreau, and M. S. Handcock, "Goodness of fit of social network models," *Journal of the american statistical association*, vol. 103, no. 481, pp. 248–258, 2008.
